# CuI-catalyzed synthesis of multisubstituted pyrido[1,2-*a*]pyrimidin-4-ones through tandem Ullmann-type C–N cross-coupling and intramolecular amidation reaction†

**DOI:** 10.1039/d3ra04454h

**Published:** 2023-08-14

**Authors:** Baichuan Mo, Chunxia Chen, Jinsong Peng

**Affiliations:** a College of Chemistry, Chemical Engineering and Resource Utilization, Northeast Forestry University No. 26 Hexing Road Harbin 150040 P. R. China ccx1759@163.com jspeng1998@nefu.edu.cn; b Material Science and Engineering College, Northeast Forestry University No. 26 Hexing Road Harbin 150040 P. R. China

## Abstract

Various multi-substituted pyrido[1,2-*a*]pyrimidin-4-ones were synthesized *via* a one-pot tandem CuI-catalyzed C–N bond formation/intramolecular amidation reaction at 130 °C in DMF. This protocol features simple operation, broad substrate scope, good functional group tolerance and gram scale preparation, thus allowing practical and modular synthesis of pyrido[1,2-*a*]pyrimidin-4-ones from readily available 2-halopyridine and (*Z*)-3-amino-3-arylacrylate ester in good to excellent yields.

## Abstract 2

Starting from 2-halopyridines and ester (*Z*)-3-amino-3-arylacrylates, one-pot tandem syntheses of diverse pyrido[1,2-*a*]pyrimidin-4-ones have been established *via* CuI-catalyzed Ullmann-type C–N bond formation/intramolecular amidation reaction.

## Graphical abstract

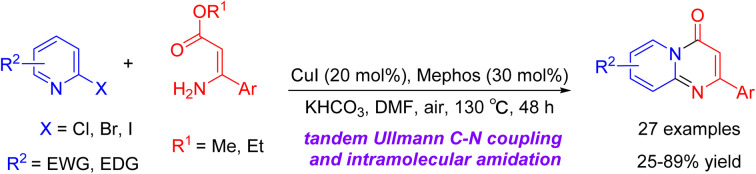

## 1. Introduction

**Fig. 1:**
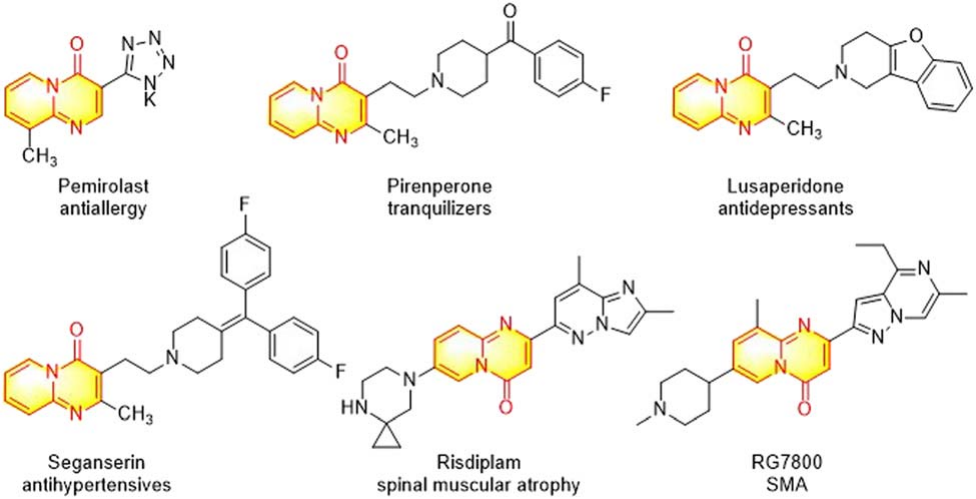
Selected drugs containing the pyrido[1,2-*a*]pyrimidin-4-one scaffold.

The nitrogen-containing bicyclic heterocycles have attracted considerable attention due to their wide applications in pharmaceuticals, agrochemicals and material sciences.^1–3^ In particular, pyrido[1,2-*a*]pyrimidine scaffolds have emerged as one of the most important N-fused bicyclic heterocycles for drug discovery and development,^4^ and numerous pyrido[1,2-*a*]pyrimidin-4-ones have exhibited a wide range of biological activities such as in antipsychotic agents,^5^ tranquilizers,^6^ antioxidants,^7^ anticancer agents,^8^ antiulcer agents,^9^ antihypertensives,^10^ antidepressants,^11^ antiallergics,^12^ antiplasmodial falcipain-2 inhibitors,^13^ and spinal muscular atrophy (SMA) drugs.^14^ Several pyrido[1,2-*a*]pyrimidin-4-one derivatives, such as pirenperone, seganserin, lusaperidone, and risdiplam have been applied in clinical trials for decades (Fig. 1).

Given the importance of pyrido[1,2-*a*]pyrimidin-4-ones in drug discovery, much efforts have been focused on the synthetic strategies and methods to construct and diversify these scaffolds efficiently,^15^ and the most frequently used approaches are based on acid-catalyzed condensation reaction or thermal cyclization.^16^ However, these conventional protocols suffer from some disadvantages such as harsh and corrosive conditions, high reaction temperatures, and limited substrate scope. Therefore, the design and development of more milder and sustainable processes for the synthesis of pyrido[1,2-*a*]pyrimidin-4-ones is imperative. Over the past few years, several transition metal-catalyzed protocols have been well established for the rapid buildup and modification of pyridopyrimidinone core, enclosing the following approaches (i) Pd-catalyzed regioselective C–H alkenylation at the 3 site,^17^ (ii) Pd-catalyzed enolic C–OH activation-arylation pathway,^18^ (iii) Pd-catalyzed carbonylative cycloamidation of ketoimines,^19^ (iv) Mn-catalyzed carbonylative alkyne annulations of 2-pyridyl hydrazone,^20^ (v) Ag-catalyzed one-pot cyclization of 2-aminopyridines and alkynoates,^21^ (vi) Pd-catalyzed Ag(i)-promoted C–H arylation at the 3 site with haloarenes as the substrates,^22^ (vii) Rh-catalyzed three-component coupling of aldehydes, 2-aminopyridines, and diazo esters,^23^ (viii) Pd-catalyzed pyridocarbonylation.^24^

**Scheme 1:**
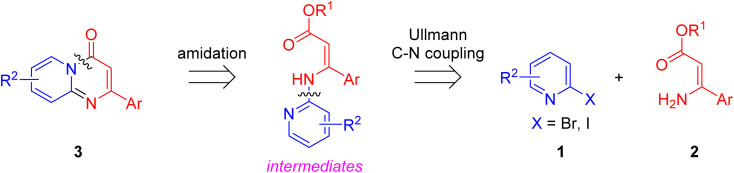
Retrosynthetic analysis of pyrido[1,2-*a*]pyrimidin-4-ones.

Although significant progress in the palladium-, rhodium- and silver-catalyzed synthetic methodologies have been made, the high cost of these catalysts led to a need to explore the use of more available and cheaper first-row metals for the synthesis of pyrido[1,2-*a*]pyrimidin-4-ones. As an alternative nonprecious metal, copper-mediated catalysis for the synthesis of pyridopyrimidine derivatives has become an important goal.^25,26^ With our continuous efforts on the Cu-catalyzed synthesis of nitrogen-containing bicyclic heterocycles,^27^ herein we design an efficient Cu-catalyzed synthesis of pyrido[1,2-*a*]pyrimidin-4-ones. Retrosynthetically, the formation of substituted pyrido[1,2-*a*]pyrimidin-4-ones 3 in a one-pot manner can be perceived through a tandem Cu-catalyzed Ullmann-type aromatic amination and intramolecular amide bond formation (Scheme 1).

## 2. Result and discussion

For the initial experiments, 2-iodopyridine 1a and ethyl (*Z*)-3-amino-3-phenylacrylate 2a as model substrates were selected for this copper-catalyzed tandem reaction to construct pyrido[1,2-*a*]pyrimidin-4-one 3aa. Our previous work revealed that the combined use of copper salts and phosphorus ligands is beneficial to the formation of Ullmann-type C–N bonds.^27*a*^ Based on the previous result, some new investigations were carried out and reaction optimization results were summarized in Table 1. First, a variety of copper catalysts such as CuO, Cu(OTf)_2_, CuBr_2_, CuSO_4_·5H_2_O, Cu(OAc)_2_, CuCl_2_, CuCl, and CuBr was examined with 2-(dicyclohexylphosphino)biphenyl as the ligand in DMA, the desired product 3aa was obtained from 9% to 23% yield (entries 1–8). However, we were pleased to find that CuI was superior to other copper catalysts and provided 3aa in 46% yield (entry 9), indicating that the nature of copper sources was essential to the transformation. We continued to optimize the effect of various bases on the reaction, mainly including NaHCO_3_, Li_2_CO_3_, KHCO_3_, K_3_PO_4_, NaO^*t*^Bu, KOH and KO^*t*^Bu (entries 10–16), potassium bicarbonate is the most suitable base for this transformation, giving 3aa in 58% yield (entry 12). Encouraged by this preliminary result, a wide range of solvents such as DMA, DMSO, DMF, toluene, CH_3_CN (entries 17–20) was then investigated, a polar solvent was crucial for this transformation and DMF was found to be better than others (entry 18). To increase the yield of 3aa, we further evaluated the effect of various ligands such as nitrogen-based bidentate ligands and phosphorus ligands (entries 18 and 21–26). In general, sterically hindered phosphine ligands gave a better result than nitrogen ligands, 3aa was afforded with 89% yield by using Mephos in DMF for 48 h (entry 23). Finally, a set of control experiments was conducted to reveal the influence of reaction temperature and time. 3aa was formed at 80 °C, 100 °C, 120 °C in significantly lower yield than 130 °C (entry 27); 12 h, 24 h, 36 h gave the corresponding product 3aa in 58%, 62% and 70% yield respectively (entry 28). The above results showed that the yield of 3aa can been improved with the increase of reaction temperature and time. Finally, we found that the amount of catalyst as well as ligand had a strong influence on the yield of the reaction. The yield of 3aa decreased continuously with the reduce of the amount of ligand or catalyst (entries 29 and 30).

**Table 1:** Optimization of one-pot tandem reaction conditions^a^

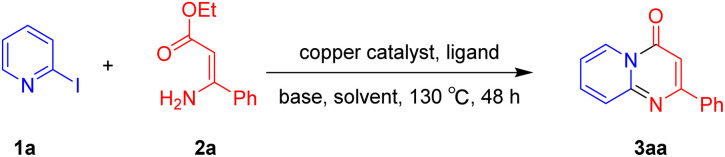				
Entry	Cu/ligand^b^	Base	Solvent	Yield^c^ (%)
1	CuO/L1	K_2_CO_3_	DMA	13
2	Cu(OTf)_2_/L1	K_2_CO_3_	DMA	15
3	CuBr_2_/L1	K_2_CO_3_	DMA	17
4	CuSO_4_·5H_2_O/L1	K_2_CO_3_	DMA	9
5	Cu(OAc)_2_/L1	K_2_CO_3_	DMA	23
6	CuCl_2_/L1	K_2_CO_3_	DMA	21
7	CuCl/L1	K_2_CO_3_	DMA	12
8	CuBr/L1	K_2_CO_3_	DMA	14
9	CuI/L1	K_2_CO_3_	DMA	46
10	CuI/L1	NaHCO_3_	DMA	26
11	CuI/L1	Li_2_CO_3_	DMA	23
12	CuI/L1	KHCO_3_	DMA	58
13	CuI/L1	K_3_PO_4_	DMA	28
14	CuI/L1	NaO^*t*^Bu	DMA	33
15	CuI/L1	KOH	DMA	18
16	CuI/L1	KO^*t*^Bu	DMA	32
17	CuI/L1	KHCO_3_	DMSO	65
18	CuI/L1	KHCO_3_	DMF	71
19	CuI/L1	KHCO_3_	Toluene	62
20	CuI/L1	KHCO_3_	CH_3_CN	60
21	CuI/L2	KHCO_3_	DMF	69
22	CuI/L3	KHCO_3_	DMF	61
23	CuI/L4	KHCO_3_	DMF	89
24	CuI/L5	KHCO_3_	DMF	80
25	CuI/L6	KHCO_3_	DMF	78
26	CuI/L7	KHCO_3_	DMF	75
27^d^	CuI/L4	KHCO_3_	DMF	55^e^/67^f^/72^g^
28^h^	CuI/L4	KHCO_3_	DMF	58^i^/62^j^/70^k^
29^l^	CuI/L4	KHCO_3_	DMF	36^m^/68^n^/75^o^
30^p^	CuI/L4	KHCO_3_	DMF	53^q^/60^r^/67^s^

a Reaction conditions: 1a (0.2 mmol), 2a (0.24 mmol), base (0.4 mmol), catalyst (20 mol%, 0.04 mmol), ligand (30 mol%, 0.06 mmol), air, 130 °C, 48 h, solvent (1.0 mL).

b L1 = 2-(dicyclohexylphosphino)biphenyl, L2 = 1,10-phenanthroline, L3 = 2,2′-bipyridine, L4 = Mephos, L5 = Davephos, L6 = triphenylphosphine, L7 = tricyclohexylphosphine.

c Isolated yield.

d 48 h.

e 80 °C.

f 100 °C.

g 120 °C.

h 130 °C.

i 12 h.

j 24 h.

k 36 h.

l L4 = 30 mol%.

m CuI = 5 mol%.

n CuI = 10 mol%.

o CuI = 15 mol%.

p CuI = 20 mol%.

q L4 = 10 mol%.

r L4 = 15 mol%.

s L4 = 20 mol%.

With the optimized reaction conditions in hand, the scope and generality of this synthetic method were then assessed (Tables 2 and 3). Using 2-iodopyridine 1a as the partner, the scope and limitations of different enamines 2 were firstly investigated in this Cu-catalyzed tandem Ullmann and amidation reaction. As shown in Table 2, a great variety of ester (*Z*)-3-aminoacrylates 2a–2p can be smoothly converted into the corresponding pyridopyrimidin-4-ones in moderate to good yields (29–89%). Several functional groups (such as Me, OMe, F, Cl and CF_3_) are tolerated under the optimized reaction conditions. The reaction of both ethyl and methyl (*Z*)-3-amino-3-phenylacrylate (2a and 2a′) gave the product 3aa in high yields (89% and 85%, respectively, entries 1 and 2). The nature of the ester motifs did not seem to affect the efficiency of this transformation. For ester (*Z*)-3-amino-3-arylacrylates, both electron-donating (Me and OMe) and electron-withdrawing substituents (F, Cl, Br and CF_3_) can be incorporated at the ortho (2b, 2i and 2l), meta (2c, 2f, 2h, 2k and 2o), and para (2d, 2e, 2g, 2j, 2m and 2n) position of 3-aryl moiety, providing pyridopyrimidine-4-ones. The electronic nature of the aromatic motifs seemed to affect the efficiency to some extent. In general, substrates containing electron-donating groups were more reactive than those bearing electron-withdrawing substituents and gave higher yields. However, when fluoro-substituted substrates 2j and 2k were used, the corresponding products can be obtained in good yields (77% and 71%, entries 11 and 12). When the scope of substrates was extended to aliphatic enamines, for example, ester (*Z*)-3-aminobut-2-enoate 2p and 2p′ (entries 17 and 18), the reaction can smoothly happen to give the corresponding products 3ap in moderate yields.

**Table 2:** Variation of the enamine substrates^a^

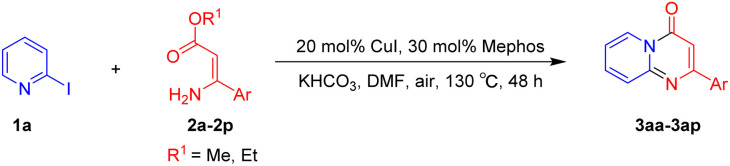							
Entry	S-2	P-3	Yield^b^	Entry	S-2	P-3	Yield^b^
1	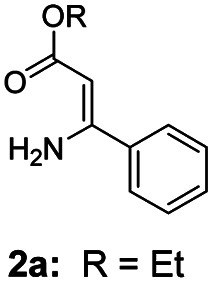	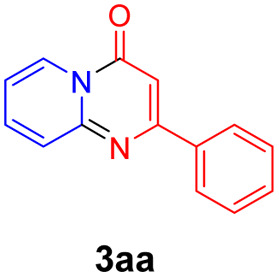	89	10	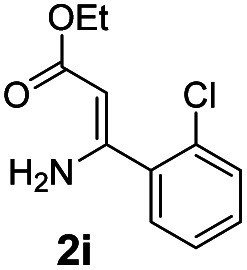	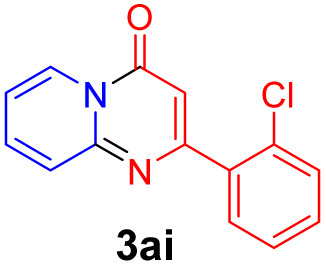	53
2	2a′: R = Me		85	11	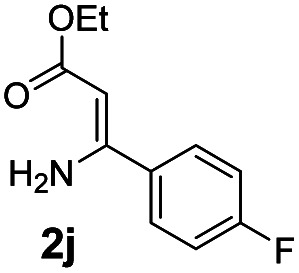	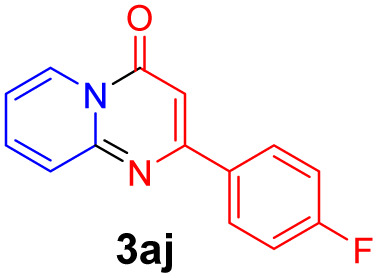	77
3	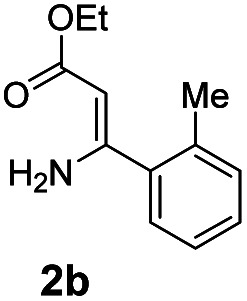	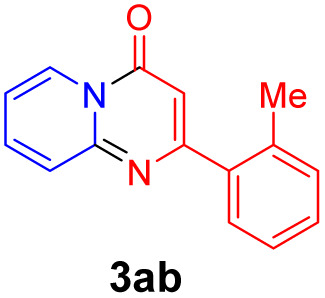	75	12	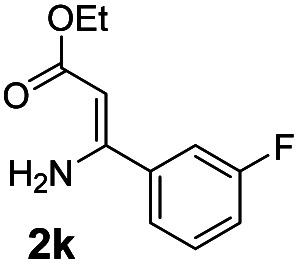	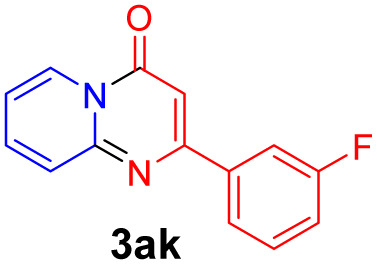	71
4	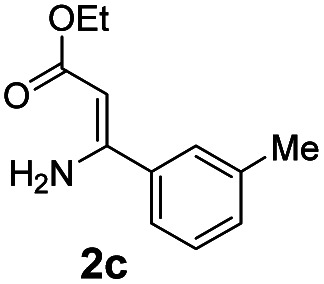	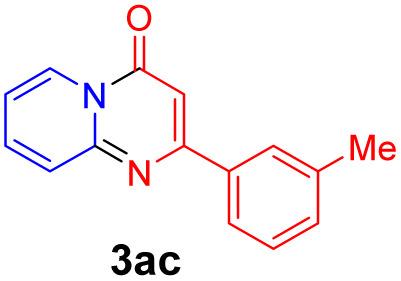	82	13	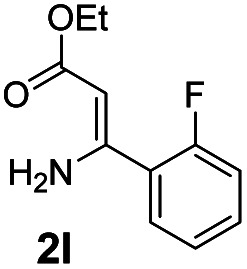	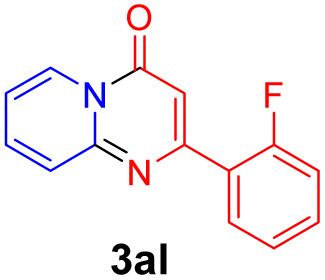	29
5	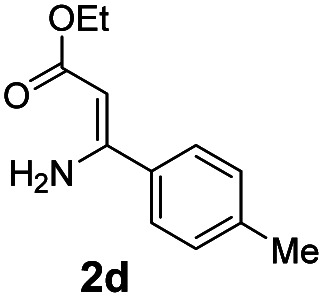	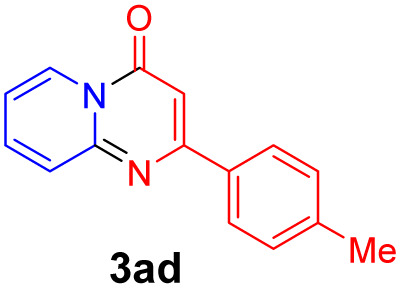	84	14	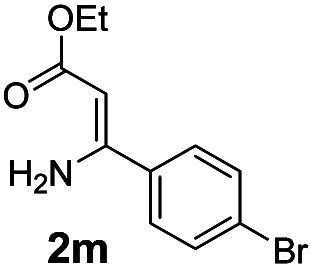	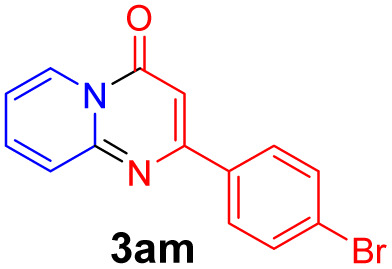	67
6	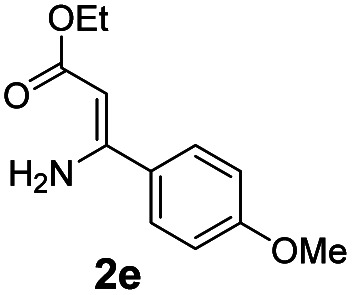	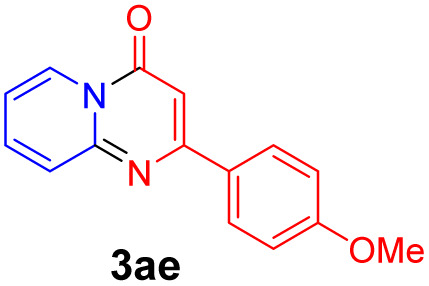	60	15	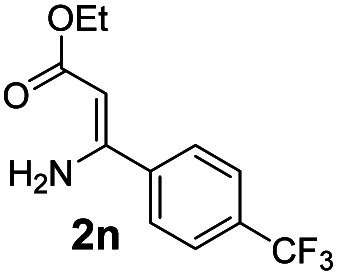	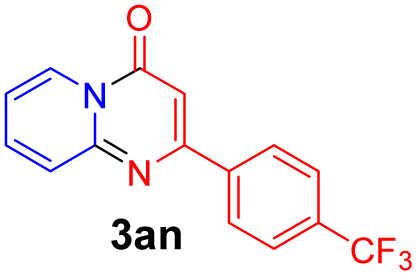	70
7	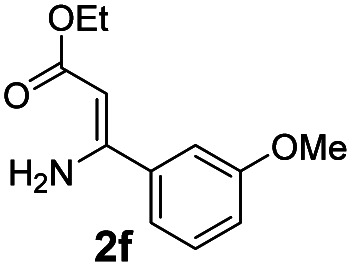	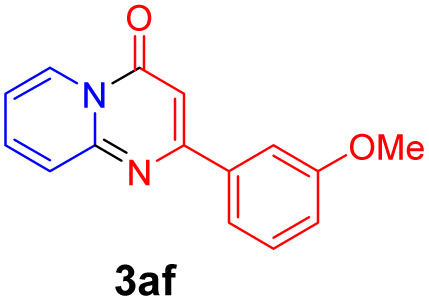	62	16	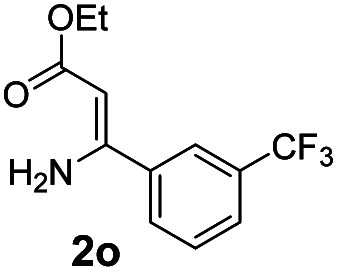	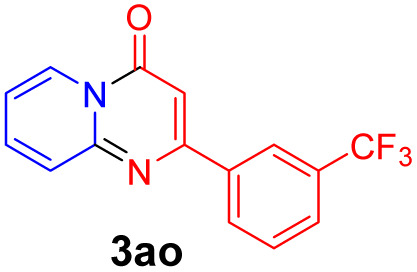	57
8	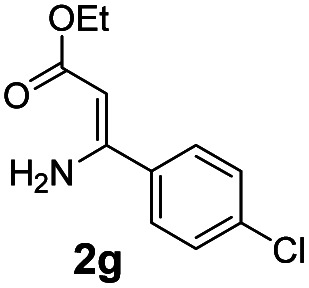	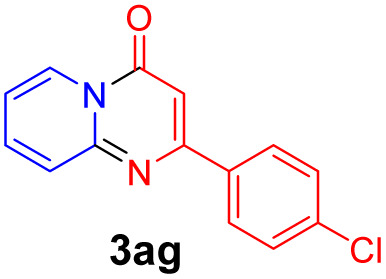	58	17	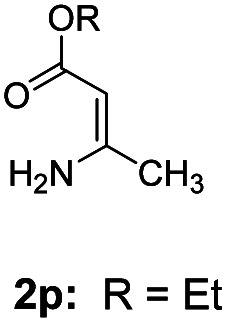	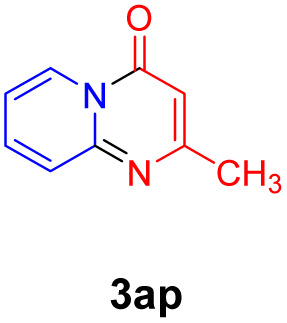	70
9	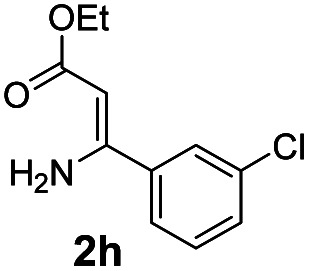	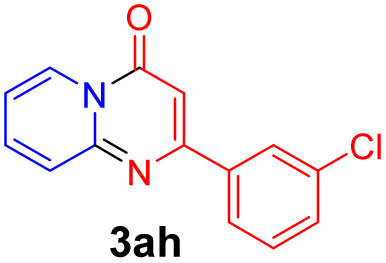	49	18	2p′: R = Me		57

a Reaction conditions: 2 (0.24 mmol), 1a (0.2 mmol), CuI (20 mol%), Mephos (30 mol%), KHCO_3_ (0.4 mmol), DMF (1.0 mL), air, 130 °C, 48 h.

b Yield of the isolated product.

**Table 3:** Variation of the 2-halopyridine substrates^a^

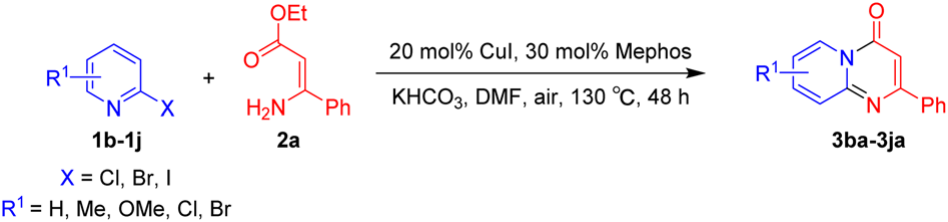							
Entry	S-1	P-3	Yield^b^	Entry	S-1	P-3	Yield^b^
1	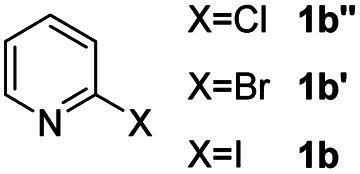	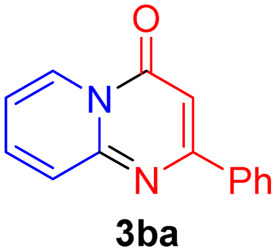	31	6	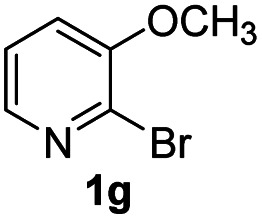	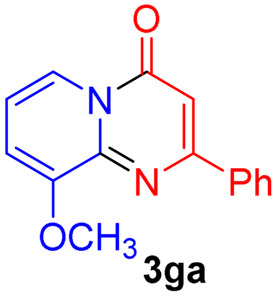	51
			64				
			89 (73)^c^				
2	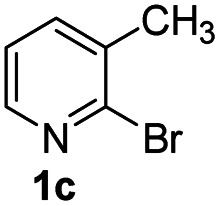	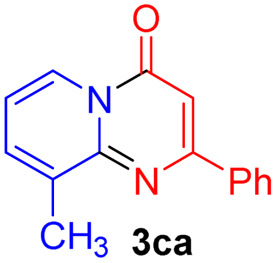	51	7	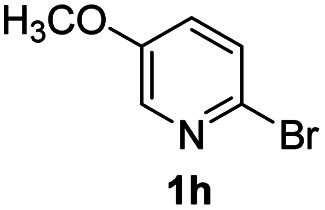	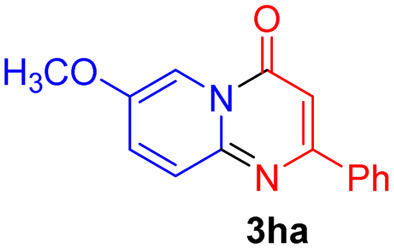	43
3	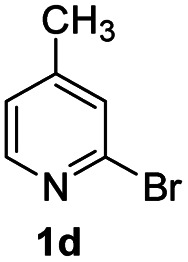	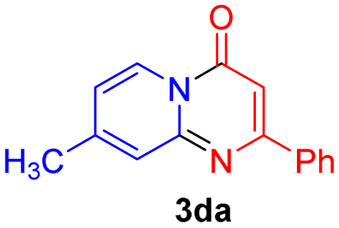	51	8	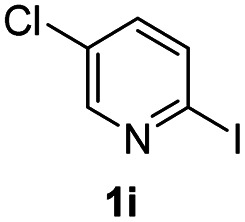	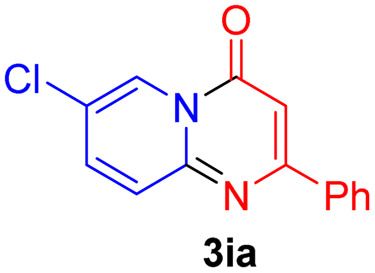	62
4	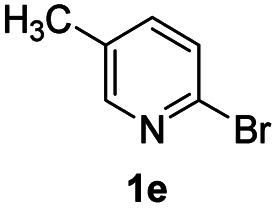	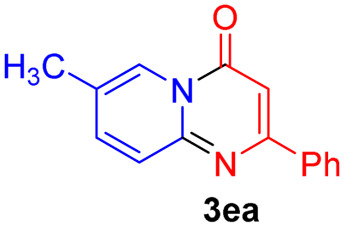	50	9	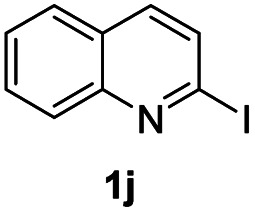	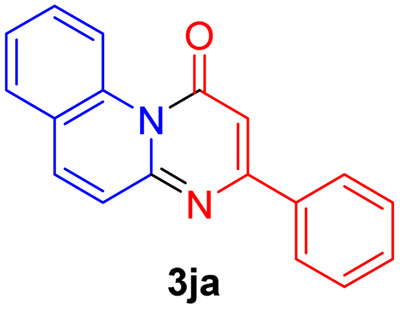	48
5	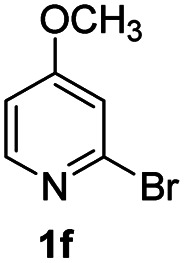	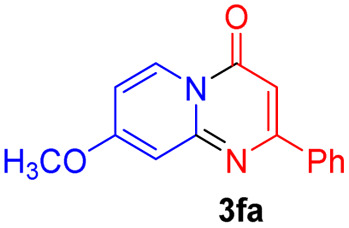	25				

a Reaction conditions: 2a (0.24 mmol), 1 (0.2 mmol), CuI (20 mol%), Mephos (30 mol%), KHCO_3_ (0.4 mmol), DMF (1.0 mL), air, 130 °C, 48 h.

b Yield of the isolated product.

c One millimole scale.

Using ethyl (*Z*)-3-amino-3-phenylacrylate 2a as the substrate, 2-halopyridines were then investigated in this one-pot sequential annulation reaction (Table 3). Firstly, the reaction activity of 2-halopyridine was examined, 2-iodopyridine 1b can smoothly be converted to the desired products 3ba in excellent yield, however, the use of bromo or chloro-substituted substrates (1b′ and 1b′′, entry 1) afforded inferior results than their iodo analogue, providing 64% and 31% yield respectively. The yield for 2-halopyridines follows the order pyridinyl iodide > bromide > chloride. The result was probably attributed to poorer tendency of C–Br and C–Cl to undergo oxidative addition to active copper species. Various 2-halopyridines with substituents such as Me, OMe and Cl on the pyridine moiety were next explored, and the corresponding products 3ba–3ia can be obtained in 31–64% yields. The electronic nature of these substituents seemed to have little effect on the reaction outcome, the similar yields were obtained for the selected 2-bromopyridine substrates except 1f with methoxy at the meta position (entries 2–7). The incorporation of the sterically hindering methyl or methoxy group in the *ortho* positions of halogen seemed not to affect the reaction, and the corresponding products can be obtained in the same yield (51%, entries 2 and 6). To our delight, the reaction conditions was also suitable for 2-iodoquinoline, which could smoothly convert into the corresponding product (entry 9). Finally, the reaction can be carried out on a 1.0 mmol scale to provide the target product 3ba in 73% yield (entry 1), demonstrating its utility in organic synthesis.

**Scheme 2:**
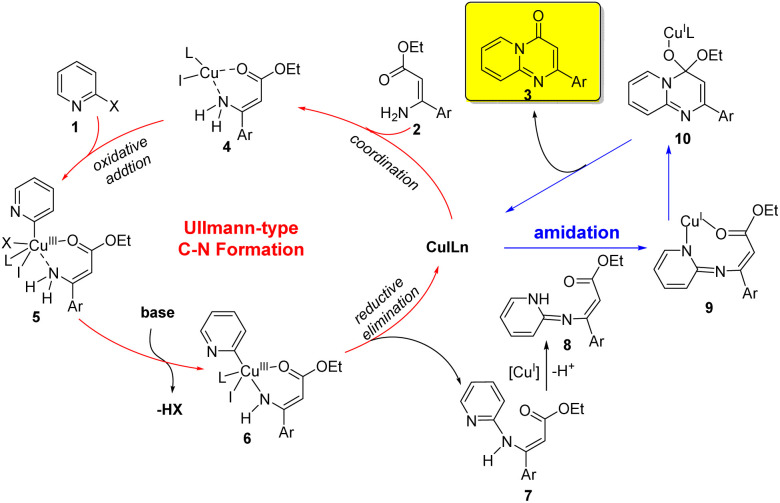
Proposed mechanism for the formation of pyrido[1,2-*a*]pyrimidinones.

Based on the above experimental results, a plausible mechanism for the copper-catalyzed sequential Ullmann-type C–N formation and amidation reaction was outlined in Scheme 2. The initial step involved the coordination of ester (*Z*)-3-amino-3-arylacrylate 2 to the Cu(i) species to give chelate 4, which underwent an intermolecular oxidative addition of 2-halopyridine 1 to form a Cu(iii) species 5. A base-promoted deprotonation of amino group led to form a Cu–N bond and provide a complex 6. The resulting complex 6 underwent reductive elimination to give an intermediate 7 and regenerate the active Cu(i) catalyst (Ullmann-type C–N coupling process). Subsequent, 7 was deprotonated to form intermediate 8, which could afford the product 3 through copper-catalyzed intramolecular amide bond formation. It's noteworthy that CuI played two critical roles in this reaction, which could not only catalyze the intermolecular C–N formation through Ullmann reaction, but also promote the subsequent intramolecular cyclization *via* amide bond formation.^26^

## 3. Conclusions

In summary, a novel and efficient approach to construct biologically relevant pyrido[1,2-*a*]pyrimidin-4-ones have been developed through Cu-catalyzed sequential Ullmann-type C–N formation and intramolecular amidation starting from 2-halopyridines and ester (*Z*)-3-amino-3-arylacrylates. The reaction has some advantages of broad functional group compatibility, facile scalability and easy product derivatization. At the same time, considering their potential biological activities, the method could be further employed to construct more complex biological molecules in synthetic and pharmaceutical chemistry.

## 4. Experimental section

### 4.1. General information

Chemicals were all purchased from commercial supplies and used without further purification unless otherwise stated. Solvents were dried and purified according to the standard procedures before use. Reactions were monitored by analytical thin-layer chromatography (TLC). All reactions were conducted in dried glassware. Reaction products was purified by flash chromatography with 230–400 mesh silica gel. Ester (*Z*)-3-aryl-3-aminoacrylate substrates were prepared according to the literature methods.^28^ Melting points were determined on a melting point apparatus in open capillaries and were uncorrected. Infrared spectra of samples were recorded from 4000 to 500 cm^−1^ in ATR (attenuated total reflectance) mode using an FT-IR instrument. ^1^H NMR spectra were recorded on a 400 or 500 MHz spectrometer. ^13^C NMR spectra were recorded at 126 or 151 MHz. Unless otherwise stated, deuterochloroform (CDCl_3_) was used as a solvent. Chemical shifts (*δ*) are given in parts per million downfield relative to tetramethylsilane (TMS). Chemical shifts for carbon resonances are reported in parts per million and are referenced to the carbon resonance of the solvent CHCl_3_ (*δ* = 77.16 ppm). The splitting patterns are reported as s (singlet), d (doublet), dd (double doublet), td (triplet of doublet), t (triplet), q (quartet), br (broad), and m (multiplet). Coupling constants are given in hertz. High-resolution mass spectra were recorded on a BIO TOF Q mass spectrometer equipped with an electrospray ion source (ESI), operated in the positive mode.

### 4.2. General procedures for the synthesis of pyridopyrimidinone derivatives

A 10 mL schlenk tube or standard vial equipped with a magnetic stirring bar was charged with 2-halopyridine (0.2 mmol, 1.0 equiv.), enamines (0.24 mmol, 1.2 equiv.), and KHCO_3_ (40 mg, 0.4 mmol, 2.0 equiv.), and then CuI (7.6 mg, 0.04 mmol, 0.2 equiv.) and Mephos (21.8 mg, 0.06 mmol, 0.3 equiv.) were added. Finally, DMF (1.0 mL) was added to the mixture *via* syringe at room temperature under air. The tube was sealed and put into a preheated oil bath at 130 °C for 48 h. The mixture was cooled to room temperature, quenched with water (3 mL), and diluted with ethyl acetate (5 mL). The layers were separated, and the aqueous layer was extracted with 3 × 5 mL of ethyl acetate. The combined organic extracts were dried over anhydrous sodium sulfate, filtered, and concentrated *in vacuo*. The crude product was then purified by flash chromatography on silica gel (H), eluting with the mixture of ethyl acetate and petroleum ether (from 1 : 4 to 1 : 3).

#### 4.2.1 2-Phenyl-4*H*-pyrido[1,2-*a*]pyrimidin-4-one (3aa)^18^

Yield, 89% (39.5 mg); white solid, mp 155–156 °C; ^1^H NMR (400 MHz, CDCl_3_) *δ* 9.07 (d, *J* = 7.1 Hz, 1H), 8.09 (dd, *J* = 6.5, 3.1 Hz, 2H), 7.73 (d, *J* = 3.6 Hz, 2H), 7.52–7.47 (m, 3H), 7.12 (dt, *J* = 7.5, 3.9 Hz, 1H), 6.91 (s, 1H). ^13^C NMR (151 MHz, CDCl_3_) *δ* 162.09, 158.66, 151.05, 137.29, 136.20, 130.67, 128.85, 127.44, 127.30, 126.81, 115.23, 100.14.

#### 4.2.2 2-(*o*-Tolyl)-4*H*-pyrido[1,2-*a*]pyrimidin-4-one (3ab)^19^

Yield, 75% (35.4 mg); white solid, mp 125–126 °C; ^1^H NMR (400 MHz, CDCl_3_) *δ* 9.13 (d, *J* = 7.1 Hz, 1H), 7.79–7.70 (m, 2H), 7.49 (d, *J* = 7.4 Hz, 1H), 7.34 (dt, *J* = 14.4, 6.6 Hz, 3H), 7.18 (t, *J* = 6.6 Hz, 1H), 6.59 (s, 1H), 2.47 (s, 3H). ^13^C NMR (151 MHz, CDCl_3_) *δ* 165.56, 158.14, 150.74, 138.68, 136.27, 135.85, 131.04, 129.27, 129.10, 127.25, 126.66, 126.07, 115.46, 104.40, 20.38.

#### 4.2.3 2-(*m*-Tolyl)-4*H*-pyrido[1,2-*a*]pyrimidin-4-one (3ac)^19^

Yield, 82% (38.7 mg); light yellow solid, mp 120–121 °C; ^1^H NMR (400 MHz, CDCl_3_) *δ* 9.04 (d, *J* = 7.1 Hz, 1H), 7.91 (s, 1H), 7.84 (d, *J* = 7.7 Hz, 1H), 7.71 (s, 1H), 7.37 (t, *J* = 7.6 Hz, 1H), 7.28 (d, *J* = 7.5 Hz, 1H), 7.14–7.08 (m, 2H), 6.89 (s, 1H), 2.44 (s, 3H). ^13^C NMR (151 MHz, CDCl_3_) *δ* 162.23, 158.61, 150.99, 138.51, 137.21, 136.14, 131.44, 128.73, 128.06, 127.25, 126.73, 124.57, 115.17, 100.12, 21.57.

#### 4.2.4 2-(*p*-Tolyl)-4*H*-pyrido[1,2-*a*]pyrimidin-4-one (3ad)^19^

Yield, 84% (39.6 mg); white solid, mp 167–168 °C; ^1^H NMR (400 MHz, CDCl_3_) *δ* 9.05 (d, *J* = 7.1 Hz, 1H), 7.99 (d, *J* = 7.7 Hz, 2H), 7.71 (s, 2H), 7.30 (d, *J* = 7.8 Hz, 2H), 7.10 (s, 1H), 6.89 (s, 1H), 2.42 (s, 3H). ^13^C NMR (151 MHz, CDCl_3_) *δ* 162.02, 158.68, 150.99, 141.06, 136.08, 134.40, 129.57, 127.36, 127.27, 126.73, 115.07, 99.60, 21.47.

#### 4.2.5 2-(4-Methoxyphenyl)-4*H*-pyrido[1,2-*a*]pyrimidin-4-one (3ae)^19^

Yield, 60% (30.2 mg); white solid, mp 174–176 °C; ^1^H NMR (400 MHz, CDCl_3_) *δ* 9.04 (d, *J* = 7.1 Hz, 1H), 8.07 (d, *J* = 8.8 Hz, 2H), 7.70 (d, *J* = 6.0 Hz, 2H), 7.08 (td, *J* = 6.9, 6.1, 2.2 Hz, 1H), 7.00 (d, *J* = 8.8 Hz, 2H), 6.85 (s, 1H), 3.87 (s, 3H). ^13^C NMR (151 MHz, CDCl_3_) *δ* 161.84, 161.57, 158.65, 150.96, 136.07, 129.57, 129.01, 127.28, 126.62, 114.93, 114.18, 98.84, 55.44.

#### 4.2.6 2-(3-Methoxyphenyl)-4*H*-pyrido[1,2-*a*]pyrimidin-4-one (3af)^8^

Yield, 62% (31.2 mg); white solid, mp 157–158 °C; ^1^H NMR (400 MHz, CDCl_3_) *δ* 9.05 (d, *J* = 7.0 Hz, 1H), 7.72 (s, 2H), 7.63 (d, *J* = 9.8 Hz, 2H), 7.39 (t, *J* = 7.9 Hz, 1H), 7.14–7.08 (m, 1H), 7.02 (d, *J* = 8.1 Hz, 1H), 6.89 (s, 1H), 3.89 (s, 3H). ^13^C NMR (151 MHz, CDCl_3_) *δ* 161.83, 159.98, 158.61, 150.95, 138.71, 136.20, 129.84, 127.25, 126.78, 119.81, 116.60, 115.26, 112.55, 100.25, 55.42.

#### 4.2.7 2-(4-Chlorophenyl)-4*H*-pyrido[1,2-*a*]pyrimidin-4-one (3ag)^19^

Yield, 58% (29.7 mg); white solid, mp 154–155 °C; ^1^H NMR (400 MHz, CDCl_3_) *δ* 9.06 (d, *J* = 7.1 Hz, 1H), 8.04 (d, *J* = 7.9 Hz, 2H), 7.78–7.69 (m, 2H), 7.47 (d, *J* = 7.9 Hz, 2H), 7.14 (t, *J* = 6.6 Hz, 1H), 6.87 (s, 1H). ^13^C NMR (151 MHz, CDCl_3_) *δ* 160.76, 158.56, 151.07, 136.91, 136.41, 135.67, 129.05, 128.74, 127.33, 126.75, 115.38, 99.88.

#### 4.2.8 2-(3-Chlorophenyl)-4*H*-pyrido[1,2-*a*]pyrimidin-4-one (3ah)^19^

Yield, 49% (25.1 mg); white solid, mp 165–166 °C; ^1^H NMR (400 MHz, CDCl_3_) *δ* 9.05 (d, *J* = 7.1 Hz, 1H), 8.11 (s, 1H), 7.91 (d, *J* = 7.2 Hz, 1H), 7.74 (q, *J* = 8.8 Hz, 2H), 7.42 (q, *J* = 8.3, 7.8 Hz, 2H), 7.14 (t, *J* = 6.6 Hz, 1H), 6.86 (s, 1H). ^13^C NMR (151 MHz, CDCl_3_) *δ* 160.44, 158.49, 151.04, 139.06, 136.47, 134.95, 130.56, 130.03, 127.64, 127.30, 126.78, 125.41, 115.48, 100.22.

#### 4.2.9 2-(2-Chlorophenyl)-4*H*-pyrido[1,2-*a*]pyrimidin-4-one (3ai)^19^

Yield, 53% (27.1 mg); white solid, mp 187–188 °C; ^1^H NMR (400 MHz, CDCl_3_) *δ* 9.12 (d, *J* = 7.1 Hz, 1H), 7.80–7.72 (m, 2H), 7.66–7.61 (m, 1H), 7.53–7.47 (m, 1H), 7.41–7.35 (m, 2H), 7.19 (t, *J* = 6.6 Hz, 1H), 6.78 (s, 1H). ^13^C NMR (151 MHz, CDCl_3_) *δ* 161.14, 156.92, 150.01, 136.61, 135.34, 131.15, 129.77, 129.44, 129.42, 126.32, 126.03, 125.72, 114.63, 104.27.

#### 4.2.10 2-(4-Fluorophenyl)-4*H*-pyrido[1,2-*a*]pyrimidin-4-one (3aj)^19^

Yield, 77% (36.9 mg); white solid, mp 214–215 °C; ^1^H NMR (400 MHz, CDCl_3_) *δ* 9.07 (d, *J* = 7.1 Hz, 1H), 8.13–8.07 (m, 2H), 7.74 (q, *J* = 8.5 Hz, 2H), 7.16 (dt, *J* = 15.0, 7.4 Hz, 3H), 6.86 (s, 1H). ^13^C NMR (151 MHz, CDCl_3_) *δ* 164.50 (d, ^1^*J*_C–F_ = 251.2 Hz), 160.95, 158.58, 151.05, 136.36, 133.36, 129.51 (d, ^3^*J*_C–F_ = 8.7 Hz), 127.32, 126.71, 115.86 (d, ^2^*J*_C–F_ = 21.7 Hz), 115.29, 99.69.

#### 4.2.11 2-(3-Fluorophenyl)-4*H*-pyrido[1,2-*a*]pyrimidin-4-one (3ak)

Yield, 71% (34 mg); white solid, mp 159–160 °C; IR (KBr, cm^−1^): 3434, 1707, 1638, 1526, 1493, 1464, 1446, 1231, 779, 756; ^1^H NMR (500 MHz, CDCl_3_) *δ* 9.07 (d, *J* = 7.1 Hz, 1H), 7.86–7.82 (m, 2H), 7.79–7.73 (m, 2H), 7.46 (td, *J* = 8.1, 5.9 Hz, 1H), 7.21–7.13 (m, 2H), 6.88 (s, 1H). ^13^C NMR (126 MHz, CDCl_3_) *δ* 162.13 (d, ^1^*J*_C–F_ = 246.1 Hz), 159.53 (d, ^4^*J*_C–F_ = 2.6 Hz), 157.50, 150.01, 138.56 (d, ^3^*J*_C–F_ = 7.5 Hz), 135.41, 129.31 (d, ^3^*J*_C–F_ = 8.1 Hz), 126.29, 125.76, 121.92 (d, ^4^*J*_C–F_ = 2.8 Hz), 116.46 (d, ^2^*J*_C–F_ = 21.4 Hz), 114.43, 113.44 (d, ^2^*J*_C–F_ = 23.1 Hz), 99.25. HRMS (ESI) *m*/*z* calcd for C_14_H_10_FN_2_O^+^ (M + H)^+^ 241.07717, found 241.07657.

#### 4.2.12 2-(2-Fluorophenyl)-4*H*-pyrido[1,2-*a*]pyrimidin-4-one (3al)

Yield, 29% (13.9 mg); white solid, mp 151–152 °C; IR (KBr, cm^−1^): 3434, 1672, 1533, 1475, 1217, 1138, 841, 755; ^1^H NMR (500 MHz, CDCl_3_) *δ* 9.00 (d, *J* = 7.1 Hz, 1H), 8.07–8.01 (m, 1H), 7.70–7.64 (m, 2H), 7.40–7.35 (m, 1H), 7.24–7.20 (m, 1H), 7.14–7.06 (m, 2H), 6.96 (d, *J* = 1.1 Hz, 1H). ^13^C NMR (126 MHz, CDCl_3_) *δ* 159.97 (d, ^1^*J*_C–F_ = 253.0 Hz), 157.29, 156.98 (d, ^4^*J*_C–F_ = 2.2 Hz), 149.86, 135.13, 130.83 (d, ^3^*J*_C–F_ = 8.8 Hz), 129.87 (d, ^4^*J*_C–F_ = 2.2 Hz), 126.22, 125.68, 124.57 (d, ^2^*J*_C–F_ = 10.7 Hz), 123.47 (d, ^4^*J*_C–F_ = 3.6 Hz), 115.52 (d, ^2^*J*_C–F_ = 22.9 Hz), 114.33, 103.74 (d, ^3^*J*_C–F_ = 10.8 Hz). HRMS (ESI) *m*/*z* calcd for C_14_H_10_FN_2_O^+^ (M + H)^+^ 241.07717, found 241.07717.

#### 4.2.13 2-(4-Bromophenyl)-4*H*-pyrido[1,2-*a*]pyrimidin-4-one (3am)^16*f*,20^

Yield, 67% (40.2 mg); white solid, mp 175–176 °C; ^1^H NMR (400 MHz, CDCl_3_) *δ* 9.06 (d, *J* = 7.1 Hz, 1H), 7.97 (d, *J* = 8.5 Hz, 1H), 7.83 (s, 2H), 7.78–7.70 (m, 2H), 7.63 (d, *J* = 8.5 Hz, 1H), 7.14 (t, *J* = 6.3 Hz, 1H), 6.87 (s, 1H). ^13^C NMR (151 MHz, CDCl_3_) *δ* 160.98, 158.56, 151.01, 138.02, 136.75, 136.43, 136.14, 132.03, 129.05, 127.34, 126.77, 125.38, 115.40, 99.88.

#### 4.2.14 2-(4-(Trifluoromethyl)phenyl)-4*H*-pyrido[1,2-*a*]pyrimidin-4-one (3an)^16*f*,20^

Yield, 70% (40.6 mg); light yellow solid, mp 172–173 °C; ^1^H NMR (400 MHz, CDCl_3_) *δ* 9.07 (d, *J* = 7.1 Hz, 1H), 8.19 (d, *J* = 8.0 Hz, 2H), 7.75 (t, *J* = 9.0 Hz, 4H), 7.16 (t, *J* = 6.6 Hz, 1H), 6.92 (s, 1H). ^13^C NMR (151 MHz, CDCl_3_) *δ* 159.4, 157.5, 150.1, 139.6, 135.5, 131.2 (q, ^2^*J*_C–F_ = 31.7 Hz, 131.5, 131.3, 131.1, 130.9), 126.7, 126.3, 125.8, 124.7 (q, ^3^*J*_C–F_ = 3.0 Hz, 124.71, 124.69, 124.67, 124.65), 122.9 (q, ^1^*J*_C–F_ = 271.8 Hz, 125.6, 123.8, 122.0, 120.2), 114.6, 99.6.

#### 4.2.15 2-(3-(Trifluoromethyl)phenyl)-4*H*-pyrido[1,2-*a*]pyrimidin-4-one (3ao)

Yield, 57% (33.1 mg); light yellow solid, mp 150–152 °C; IR (KBr, cm^−1^): 3434, 1704, 1690, 1636, 1534, 1498, 1457, 1434, 1409, 1319, 1231, 1182, 1168, 1128, 1095, 1080, 802, 765, 689; ^1^H NMR (400 MHz, CDCl_3_) *δ* 9.07 (d, *J* = 7.1 Hz, 1H), 8.41 (s, 1H), 8.22 (d, *J* = 7.8 Hz, 1H), 7.80–7.71 (m, 3H), 7.61 (t, *J* = 7.8 Hz, 1H), 7.16 (ddd, *J* = 7.7, 6.1, 2.1 Hz, 1H), 6.92 (s, 1H). ^13^C NMR (151 MHz, CDCl_3_) *δ* 159.3, 157.5, 150.1, 137.0, 135.5, 130.3 (q, ^2^*J*_C–F_ = 31.7 Hz, 130.6, 130.4, 130.2, 129.9), 129.4, 128.3, 126.3, 126.1 (q, ^3^*J*_C–F_ = 3.0 Hz, 126.14, 126.12, 126.10, 126.07), 125.8, 123.4 (q, ^3^*J*_C–F_ = 3.0 Hz, 123.43, 123.41, 123.38, 123.36), 123.0 (q, ^1^*J*_C–F_ = 273.3 Hz, 125.7, 123.9, 122.1, 120.3), 114.6, 99.2. HRMS (ESI) *m*/*z* calcd for C_15_H_10_F_3_N_2_O^+^ (M + H)^+^ 291.07397, found 291.07373.

#### 4.2.16 2-Methyl-4*H*-pyrido[1,2-*a*]pyrimidin-4-one (3ap)^16*d*^

Yield, 70% (22.4 mg); light yellow solid, mp 118–120 °C; ^1^H NMR (400 MHz, CDCl_3_) *δ* 9.02 (d, *J* = 7.1 Hz, 1H), 7.72 (t, *J* = 7.7 Hz, 1H), 7.60 (s, 1H), 7.10 (t, *J* = 6.9 Hz, 1H), 6.34 (s, 1H), 2.46 (s, 3H). ^13^C NMR (151 MHz, CDCl_3_) *δ* 165.32, 157.89, 150.74, 136.26, 127.27, 125.84, 115.03, 103.37, 24.72.

#### 4.2.17 2-Phenyl-4*H*-pyrido[1,2-*a*]pyrimidin-4-one (3ba)^19,20^

Yield, 64% (28.4 mg); white solid, mp 155–156 °C; ^1^H NMR (400 MHz, CDCl_3_) *δ* 9.08 (d, *J* = 7.0 Hz, 1H), 8.12–8.07 (m, 2H), 7.74 (s, 2H), 7.50 (s, 3H), 7.13 (s, 1H), 6.92 (s, 1H). ^13^C NMR (151 MHz, CDCl_3_) *δ* 161.07, 157.63, 150.02, 136.25, 135.15, 129.63, 127.81, 126.40, 126.26, 125.76, 114.19, 99.10.

#### 4.2.18 9-Methyl-2-phenyl-4*H*-pyrido[1,2-*a*]pyrimidin-4-one (3ca)^20^

Yield, 51% (24 mg); light yellow solid, mp 176–177 °C; ^1^H NMR (500 MHz, CDCl_3_) *δ* 8.94 (s, 1H), 8.18–8.11 (m, 2H), 7.57 (d, *J* = 6.6 Hz, 1H), 7.49 (d, *J* = 3.7 Hz, 3H), 7.01 (t, *J* = 6.9 Hz, 1H), 6.93 (s, 1H), 2.67 (s, 3H). ^13^C NMR (126 MHz, CDCl_3_) *δ* 159.48, 158.18, 149.39, 136.30, 134.29, 133.80, 129.56, 127.69, 126.37, 124.22, 113.67, 98.48, 17.27.

#### 4.2.19 8-Methyl-2-phenyl-4*H*-pyrido[1,2-*a*]pyrimidin-4-one (3da)^20^

Yield, 51% (24 mg); light yellow solid, mp 167–168 °C; ^1^H NMR (500 MHz, CDCl_3_) *δ* 8.95 (d, *J* = 7.2 Hz, 1H), 8.08–8.05 (m, 2H), 7.52–7.47 (m, 4H), 6.95 (dd, *J* = 7.2, 1.6 Hz, 1H), 6.84 (s, 1H), 2.48 (s, 3H). ^13^C NMR (126 MHz, CDCl_3_) *δ* 161.26, 157.66, 150.00, 147.24, 136.45, 129.49, 127.74, 126.35, 125.52, 123.81, 116.88, 98.21, 20.40.

#### 4.2.20 7-Methyl-2-phenyl-4*H*-pyrido[1,2-*a*]pyrimidin-4-one (3ea)^19,20^

Yield, 50% (23.6 mg); light yellow solid, mp 167–168 °C; ^1^H NMR (500 MHz, CDCl_3_) *δ* 8.86 (s, 1H), 8.07 (d, *J* = 2.4 Hz, 2H), 7.64 (d, *J* = 9.0 Hz, 1H), 7.58 (d, *J* = 10.2 Hz, 1H), 7.50–7.45 (m, 3H), 6.88 (s, 1H), 2.41 (s, 3H). ^13^C NMR (126 MHz, CDCl_3_) *δ* 160.57, 157.49, 148.92, 138.08, 136.33, 129.46, 127.75, 126.30, 125.16, 124.49, 123.66, 98.79, 17.32.

#### 4.2.21 8-Methoxy-2-phenyl-4*H*-pyrido[1,2-*a*]pyrimidin-4-one (3fa)

Yield, 25% (12.6 mg); white solid, mp 157–158 °C; IR (KBr, cm^−1^): 3438, 1673, 1645, 1500, 1470, 1457, 1403, 1354, 1223, 1162, 1019, 837, 779; ^1^H NMR (500 MHz, CDCl_3_) *δ* 8.90 (d, *J* = 7.8 Hz, 1H), 8.02 (dd, *J* = 6.5, 3.1 Hz, 2H), 7.46 (dd, *J* = 5.0, 1.8 Hz, 3H), 6.92 (d, *J* = 2.7 Hz, 1H), 6.77–6.69 (m, 2H), 3.93 (s, 3H). ^13^C NMR (126 MHz, CDCl_3_) *δ* 164.23, 161.61, 157.62, 152.07, 136.49, 129.44, 127.71, 127.32, 126.26, 109.27, 101.31, 96.98, 55.35. HRMS (ESI) *m*/*z* calcd for C_15_H_13_N_2_O_2_^+^ (M + H)^+^ 253.09715, found 253.09712.

#### 4.2.22 9-Methoxy-2-phenyl-4*H*-pyrido[1,2-*a*]pyrimidin-4-one (3ga)

Yield, 51% (25.7 mg); light yellow solid, mp 169–170 °C; IR (KBr, cm^−1^): 3435, 1677, 1634, 1496, 1472, 1415, 1277, 1162, 1042, 753; ^1^H NMR (500 MHz, CDCl_3_) *δ* 8.73–8.68 (m, 1H), 8.13–8.08 (m, 2H), 7.48 (d, *J* = 6.6 Hz, 3H), 7.07–7.00 (m, 2H), 6.94 (s, 1H), 4.06 (s, 3H). ^13^C NMR (126 MHz, CDCl_3_) *δ* 159.97, 157.70, 151.47, 144.38, 136.33, 129.54, 127.78, 126.49, 118.06, 113.17, 110.08, 99.93, 55.82. HRMS (ESI) *m*/*z* calcd for C_15_H_13_N_2_O_2_^+^ (M + H)^+^ 253.09715, found 253.09732.

#### 4.2.23 7-Methoxy-2-phenyl-4*H*-pyrido[1,2-*a*]pyrimidin-4-one (3ha)

Yield, 43% (21.6 mg); white solid, mp 167–168 °C; IR (KBr, cm^−1^): 3435, 1678, 1637, 1533, 1504, 1458, 1255, 1016, 822; ^1^H NMR (500 MHz, CDCl_3_) *δ* 8.57 (d, *J* = 2.8 Hz, 1H), 8.09–8.06 (m, 2H), 7.69 (d, *J* = 9.6 Hz, 1H), 7.53 (dd, *J* = 9.6, 2.9 Hz, 1H), 7.49 (ddt, *J* = 6.8, 5.3, 2.6 Hz, 3H), 6.92 (s, 1H), 3.96 (s, 3H). ^13^C NMR (126 MHz, CDCl_3_) *δ* 159.86, 157.52, 149.72, 147.12, 136.33, 130.57, 129.41, 127.79, 126.46, 126.26, 105.74, 98.49, 55.46. HRMS (ESI) *m*/*z* calcd for C_15_H_13_N_2_O_2_^+^ (M + H)^+^ 253.09715, found 253.09709.

#### 4.2.24 7-Chloro-2-phenyl-4*H*-pyrido[1,2-*a*]pyrimidin-4-one (3ia)^19^

Yield, 62% (31.8 mg); white solid, mp 169–170 °C; ^1^H NMR (500 MHz, CDCl_3_) *δ* 8.99 (s, 1H), 8.01–7.96 (m, 2H), 7.59 (s, 2H), 7.45–7.41 (m, 3H), 6.85 (s, 1H). ^13^C NMR (126 MHz, CDCl_3_) *δ* 160.86, 156.62, 148.32, 136.40, 135.75, 129.86, 127.85, 126.68, 126.37, 124.05, 122.86, 99.44.

#### 4.2.25 3-Phenyl-1*H*-pyrimido[1,2-*a*]quinolin-1-one (3ja)^29^

Yield, 48% (26.1 mg); white solid, mp 159–161 °C; ^1^H NMR (500 MHz, CDCl_3_) *δ* 9.85 (d, *J* = 8.8 Hz, 1H), 8.05–8.01 (m, 2H), 7.71 (d, *J* = 9.1 Hz, 1H), 7.64–7.56 (m, 2H), 7.50–7.41 (m, 4H), 7.36 (dd, *J* = 10.0, 3.6 Hz, 1H), 6.92–6.90 (m, 1H). ^13^C NMR (126 MHz, CDCl_3_) *δ* 162.99, 157.63, 150.33, 135.42, 135.19, 134.44, 129.61, 128.68, 127.79, 127.07, 126.19, 125.86, 124.00, 123.91, 121.30, 104.62.

## Conflicts of interest

There are no conflicts to declare.

## Supplementary Material

RA-013-D3RA04454H-s001
