# Supplementary material for: CuI-catalyzed synthesis of multisubstituted pyrido[1,2-a]pyrimidin-4-ones through tandem Ullmann-type C–N cross-coupling and intramolecular amidation reaction
Source: RSC Adv. 2023 Aug 14;13(35):24264–71. doi: 10.1039/d3ra04454h (PMC10424054; doi:10.1039/d3ra04454h)

Electronic Supplementary Material (ESI) for RSC Advances.
This journal is © The Royal Society of Chemistry 2023

Supporting Information

CuI-Catalyzed Synthesis of Multisubstituted Pyrido[1,2-*a*]pyrimidin-4-ones Through Tandem Ullmann-type C–N Cross-Coupling and Intramolecular Amidation Reaction

Baichuan Mo,[a,b] Chunxia Chen,*[a,b] and Jinsong Peng,*[a]

[a] College of Chemistry, Chemical Engineering and Resource Utilization, Northeast Forestry University, No. 26 Hexing Road, Harbin, 150040, P. R. China.

[b] Material Science and Engineering College, Northeast Forestry University, No. 26 Hexing Road, Harbin, 150040, P. R. China.

*E-mail: ccx1759@163.com, jspeng1998@nefu.edu.cn

Content

1. Copies of ^{1}H NMR and ^{13}C NMR spectra of the products S-2

2. High-resolution mass spectra of new compounds.....................................S-27

3. IR spectra of new compounds.. S-29

1. Copies of ^{1}H NMR and ^{13}C NMR spectra of the products

2-Phenyl-4H-pyrido[1,2-a]pyrimidin-4-one(**3aa**)

^{1}H spectrum of **3aa**

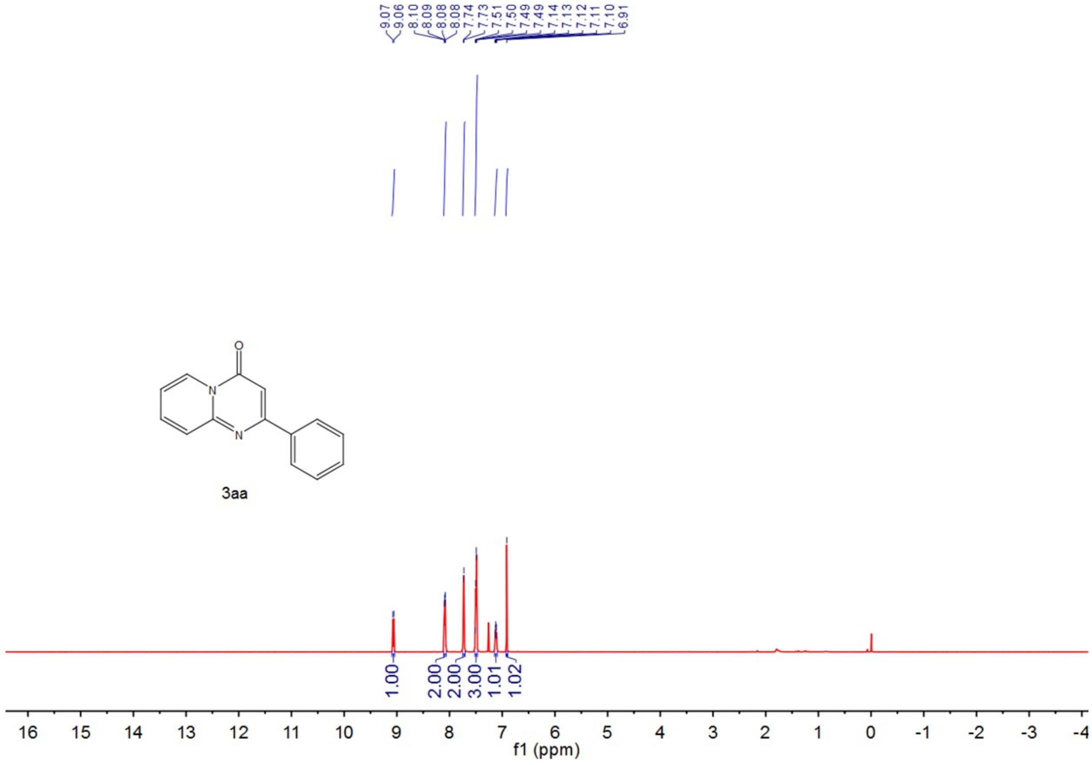

^{13}C spectrum of **3aa**

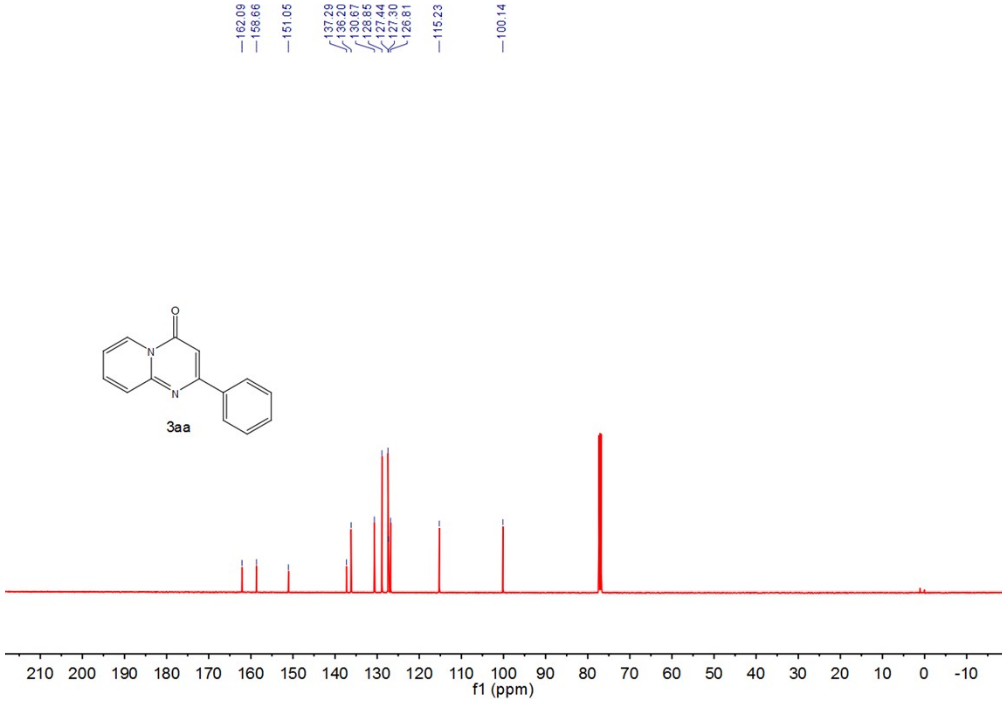

2-(o-tolyl)-4H-pyrido[1,2-a]pyrimidin-4-one(**3ab**)

^{1}H spectrum of **3ab**

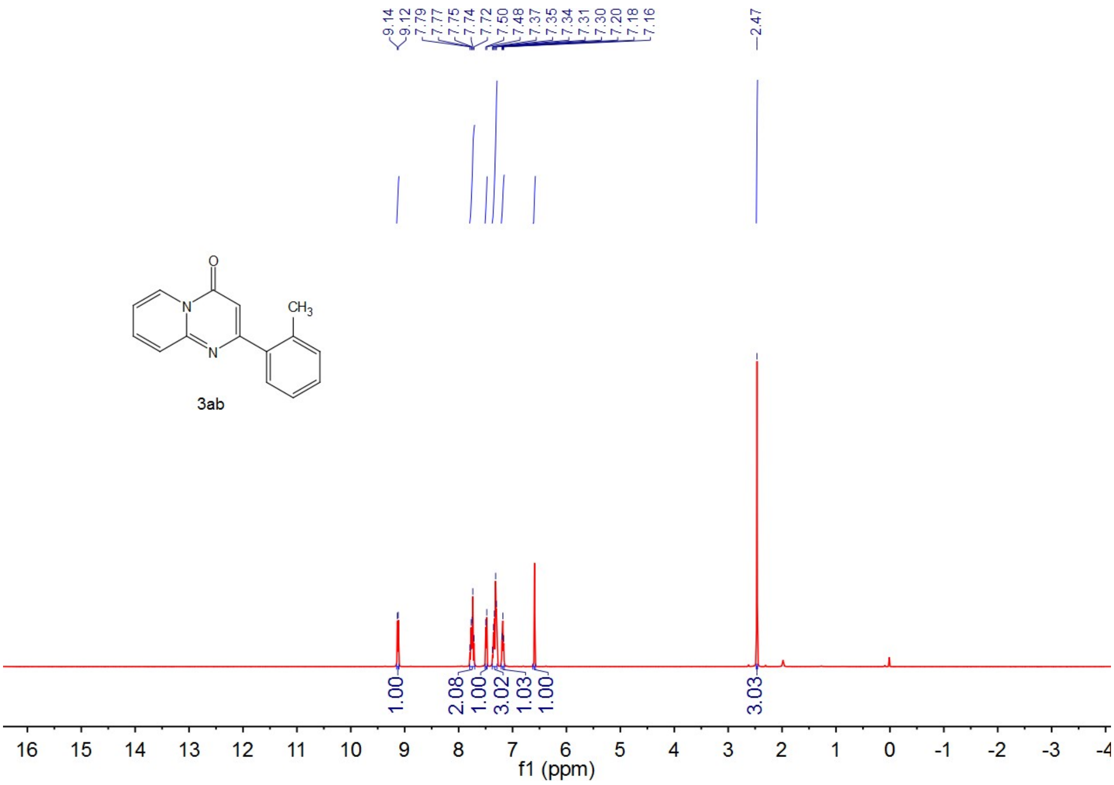

^{13}C spectrum of **3ab**

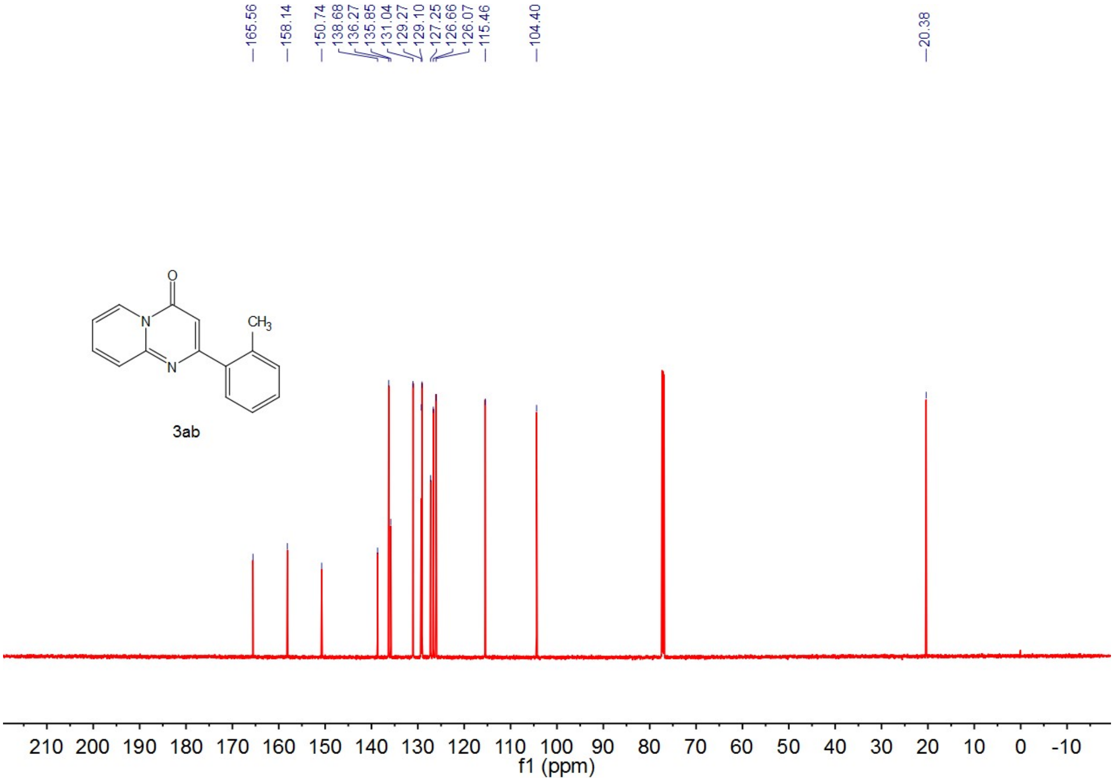

*2-(m-tolyl)-4H-pyrido[1,2-a]pyrimidin-4-one***(3ac)**

^{1}H spectrum of **3ac**

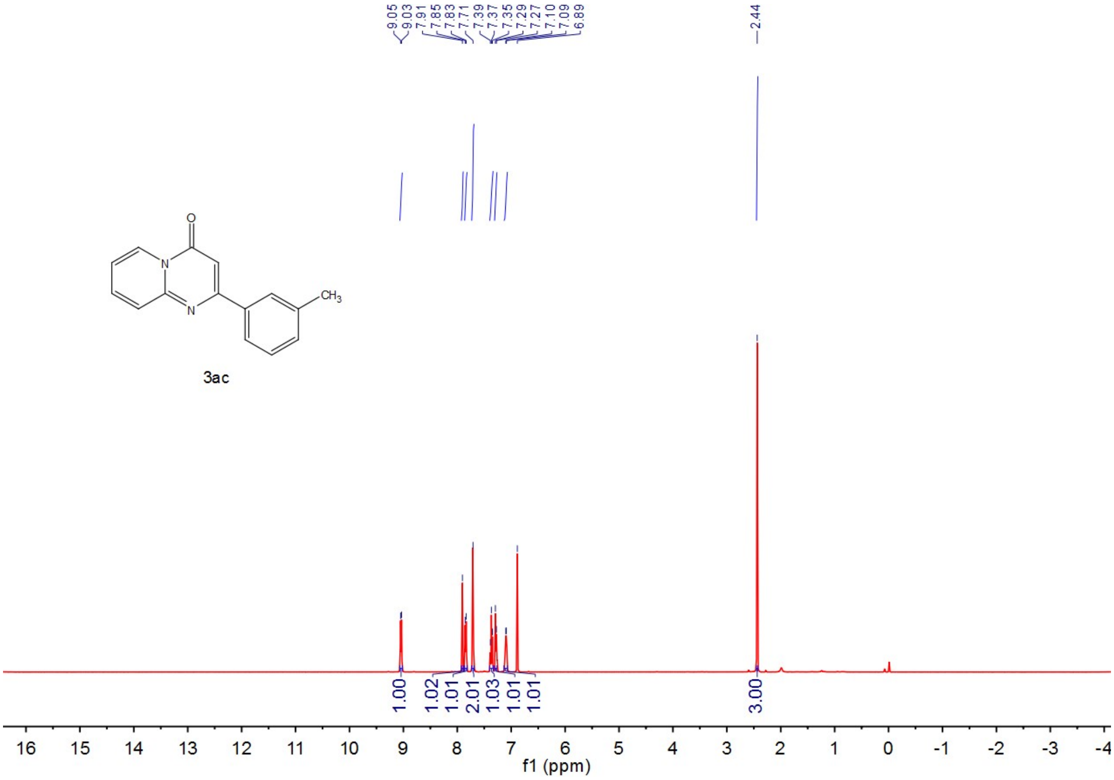

^{13}C spectrum of **3ac**

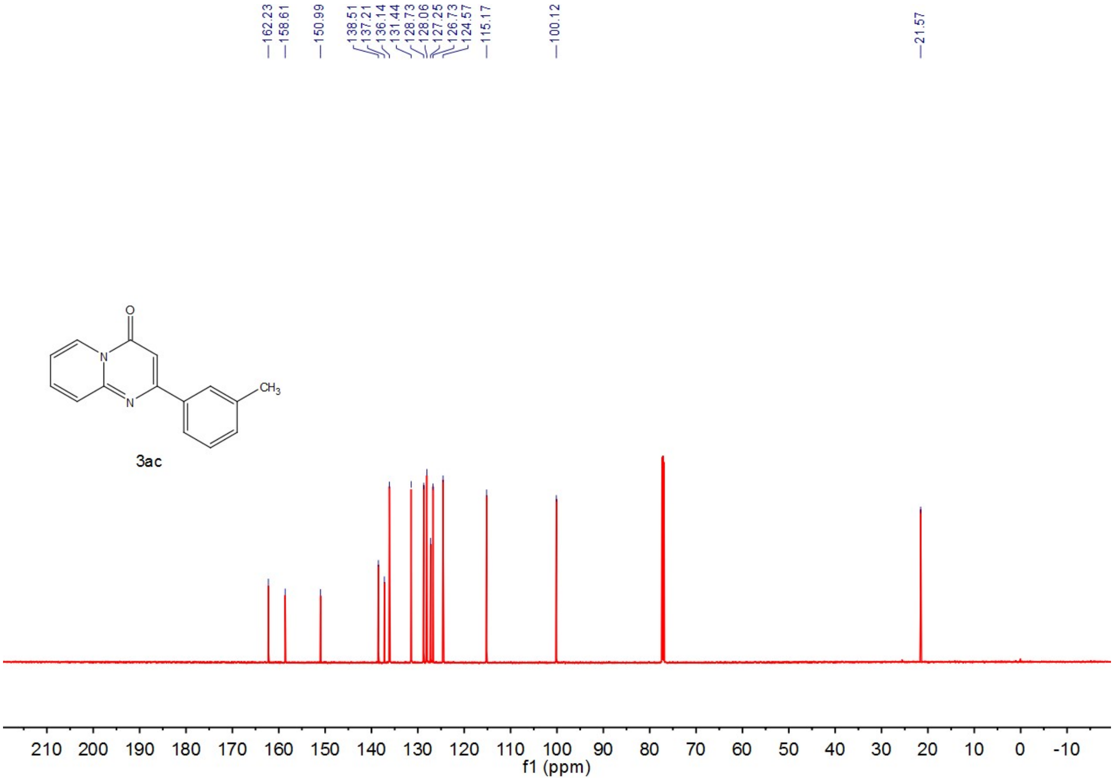

*2-(p-tolyl)-4H-pyrido[1,2-a]pyrimidin-4-one***(3ad)**

^{1}H spectrum of **3ad**

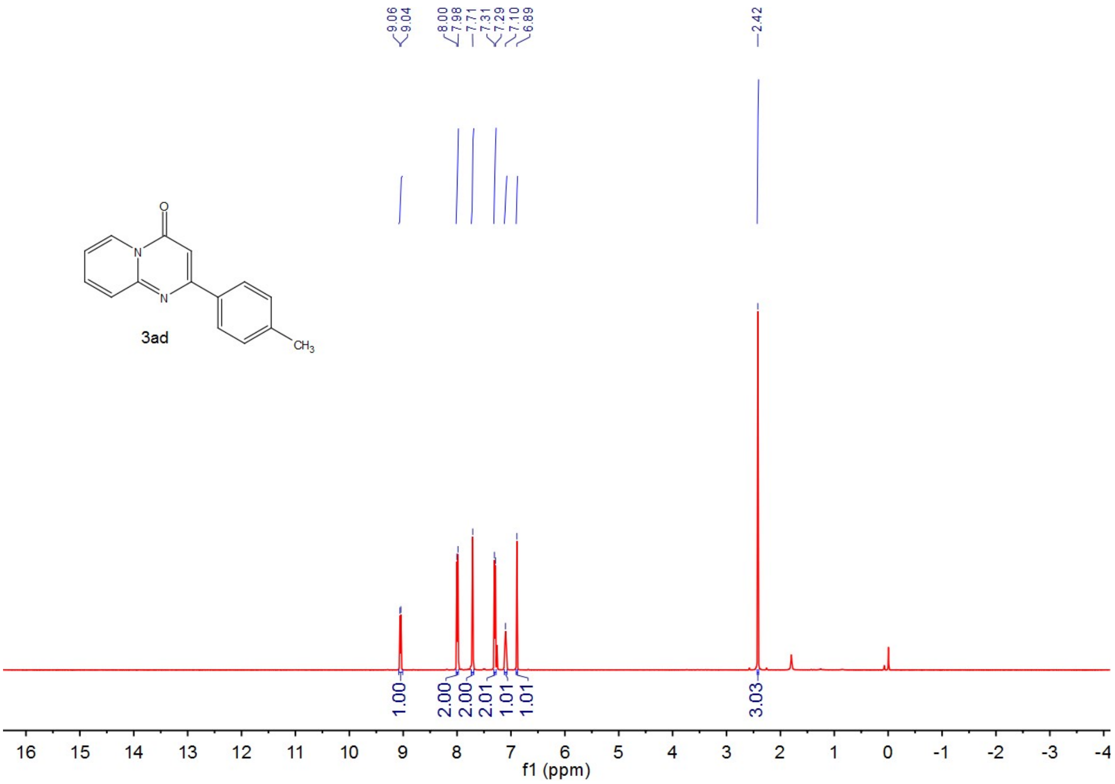

^{13}C spectrum of **3ad**

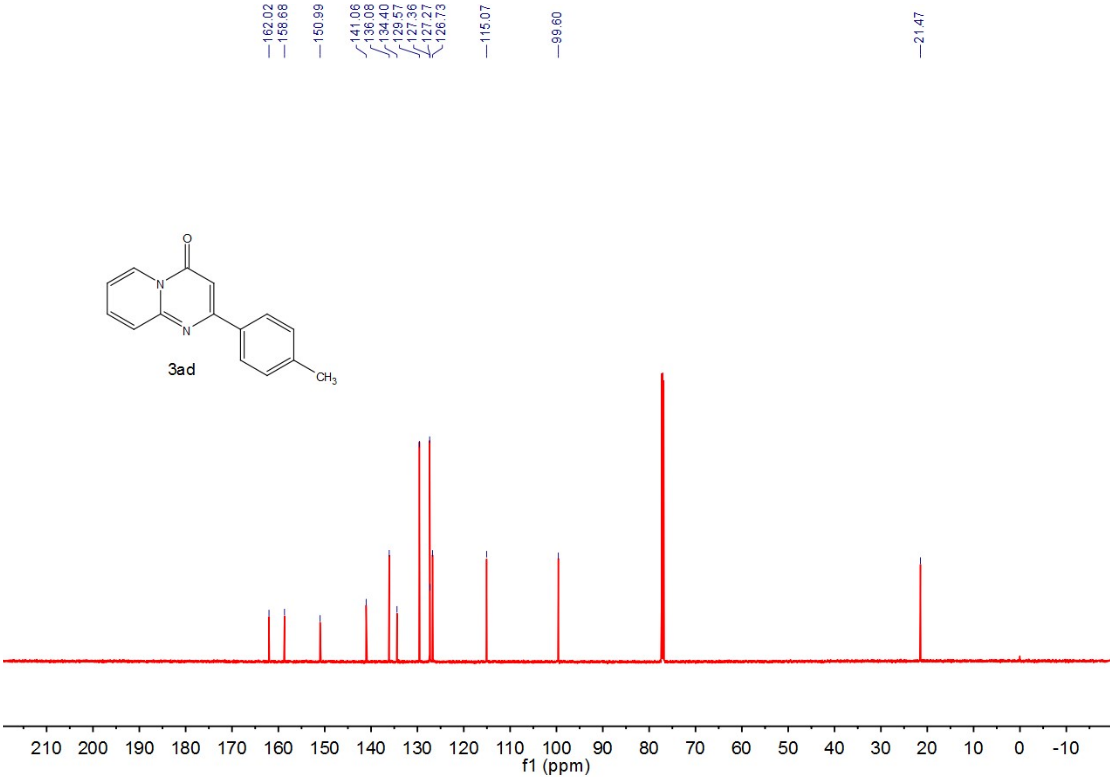

*2-(4-Methoxyphenyl)-4H-pyrido[1,2-a]pyrimidin-4-one***(3ae)**

^{1}H spectrum of **3ae**

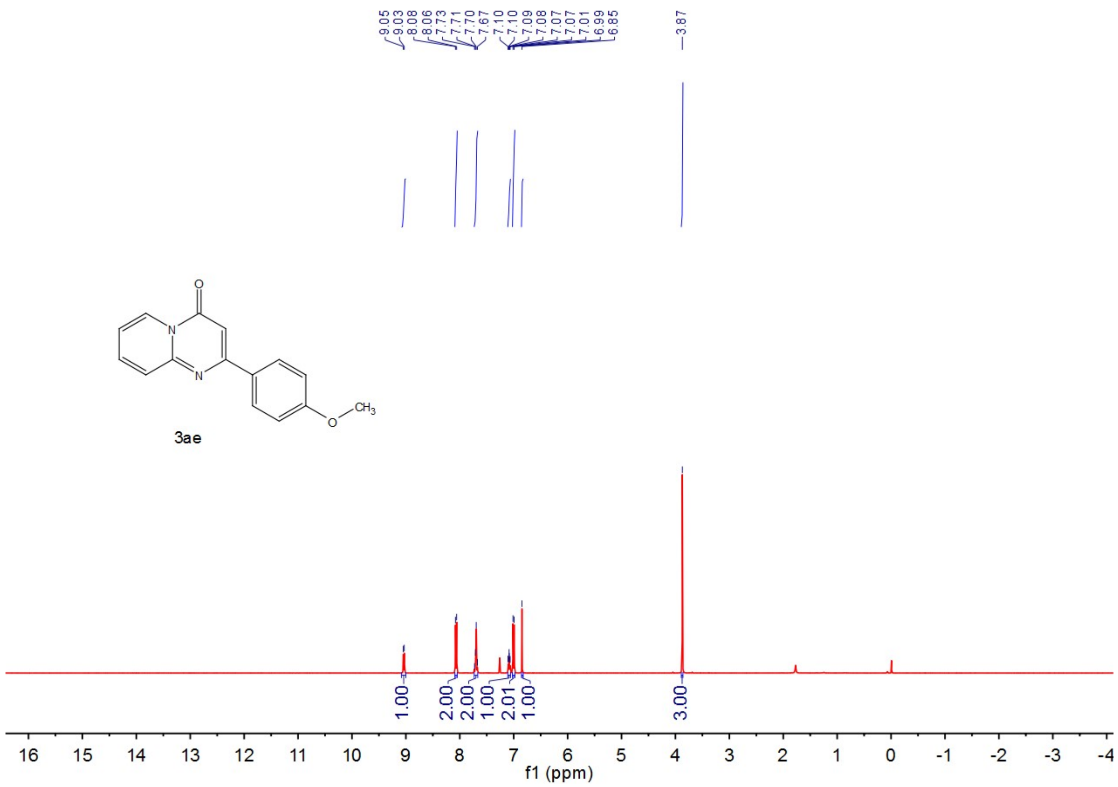

^{13}C spectrum of 3ae

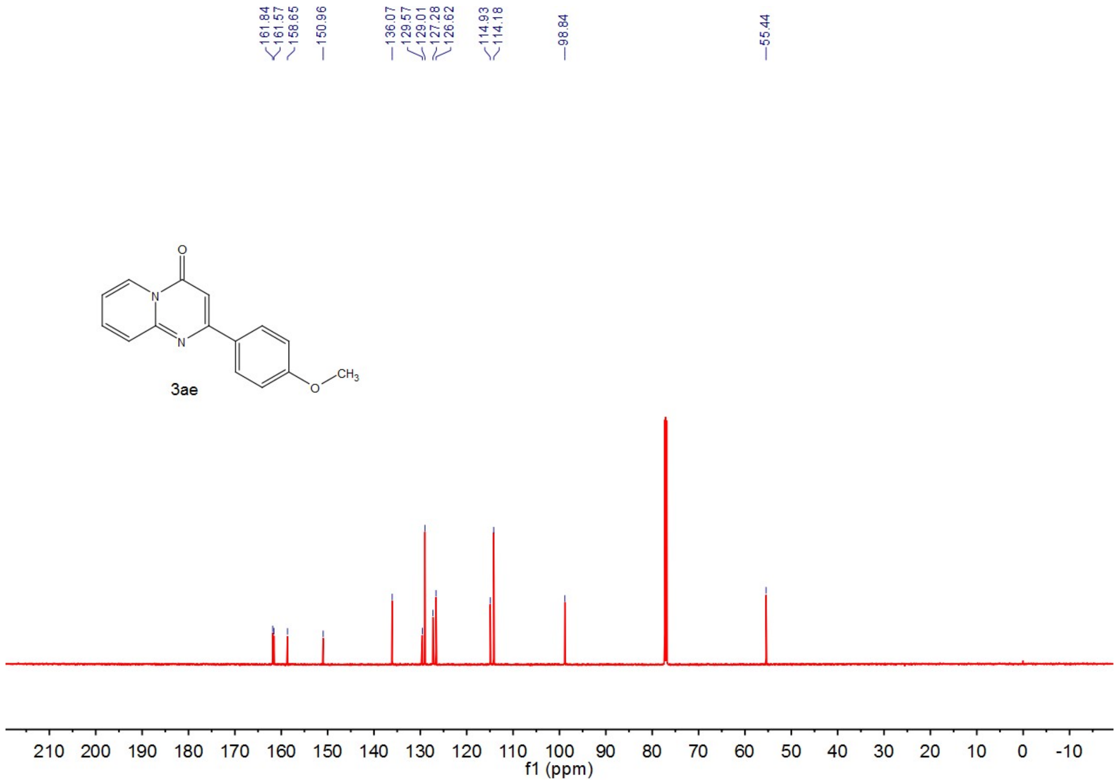

*2-(3-Methoxyphenyl)-4H-pyrido[1,2-a]pyrimidin-4-one***(3af)**

^{1}H spectrum of **3af**

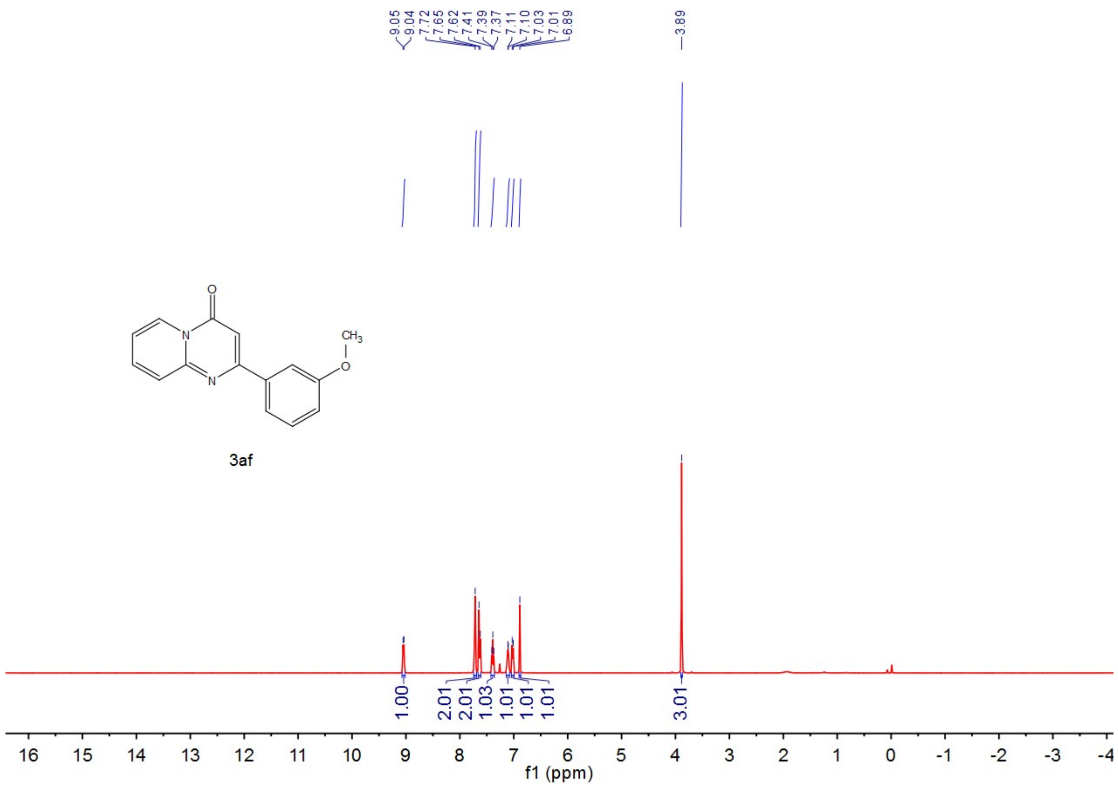

^{13}C spectrum of **3af**

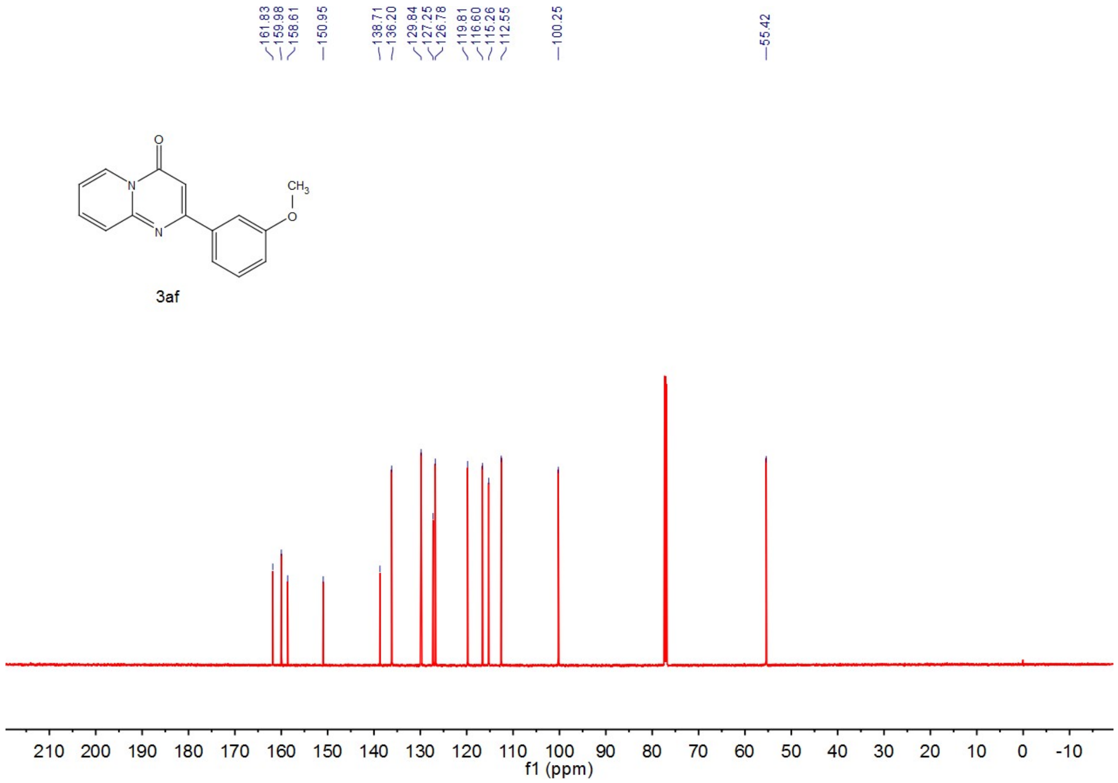

2-(4-Chlorophenyl)-4H-pyrido[1,2-a]pyrimidin-4-one(**3ag**)

^{1}H spectrum of **3ag**

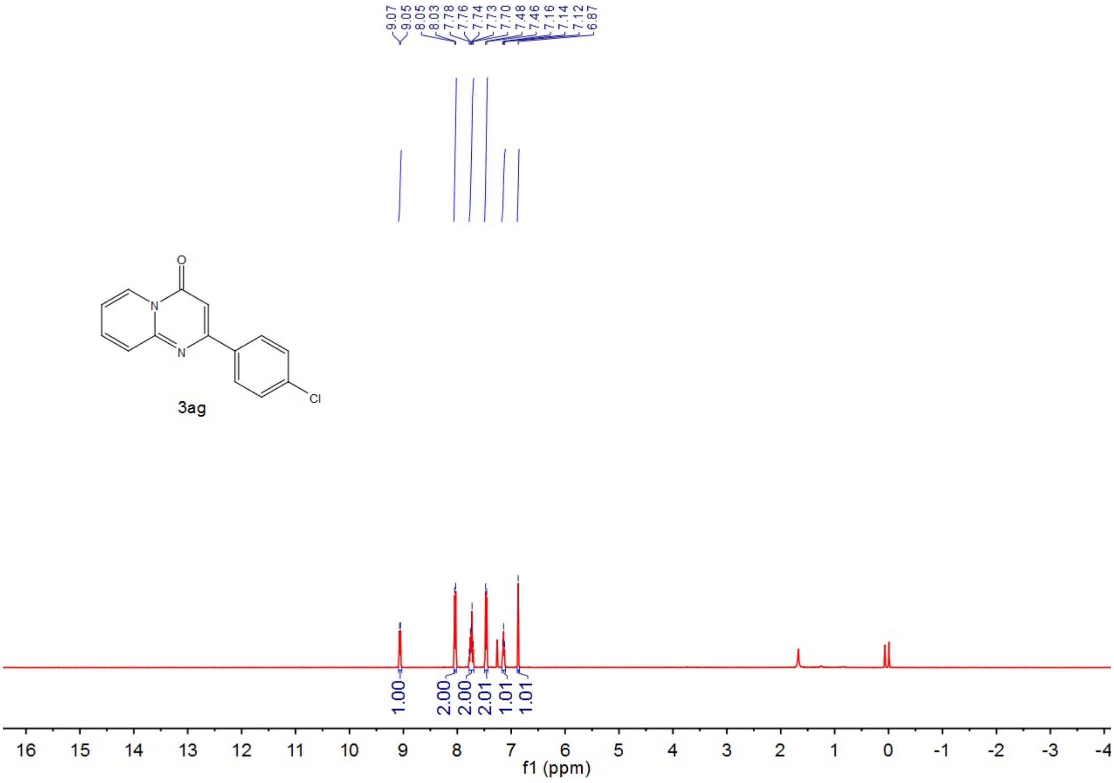

^{13}C spectrum of **3ag**

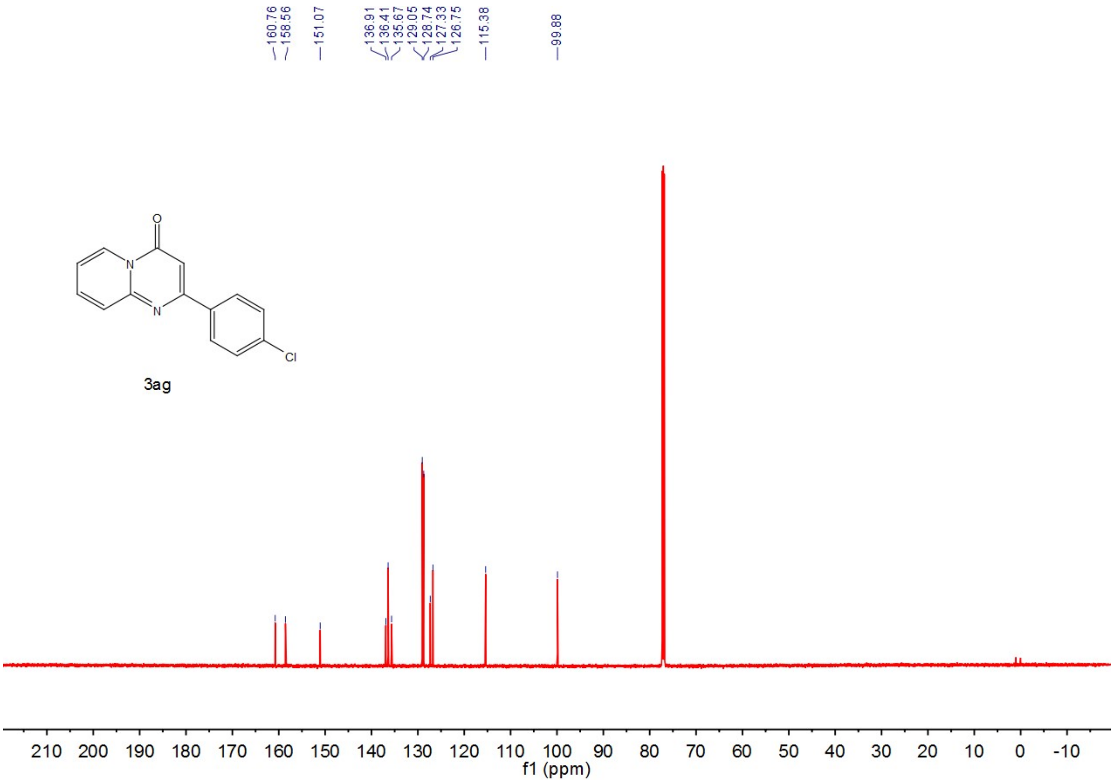

*2-(3-Chlorophenyl)-4H-pyrido[1,2-a]pyrimidin-4-one***(3ah)**

^{1}H spectrum of **3ah**

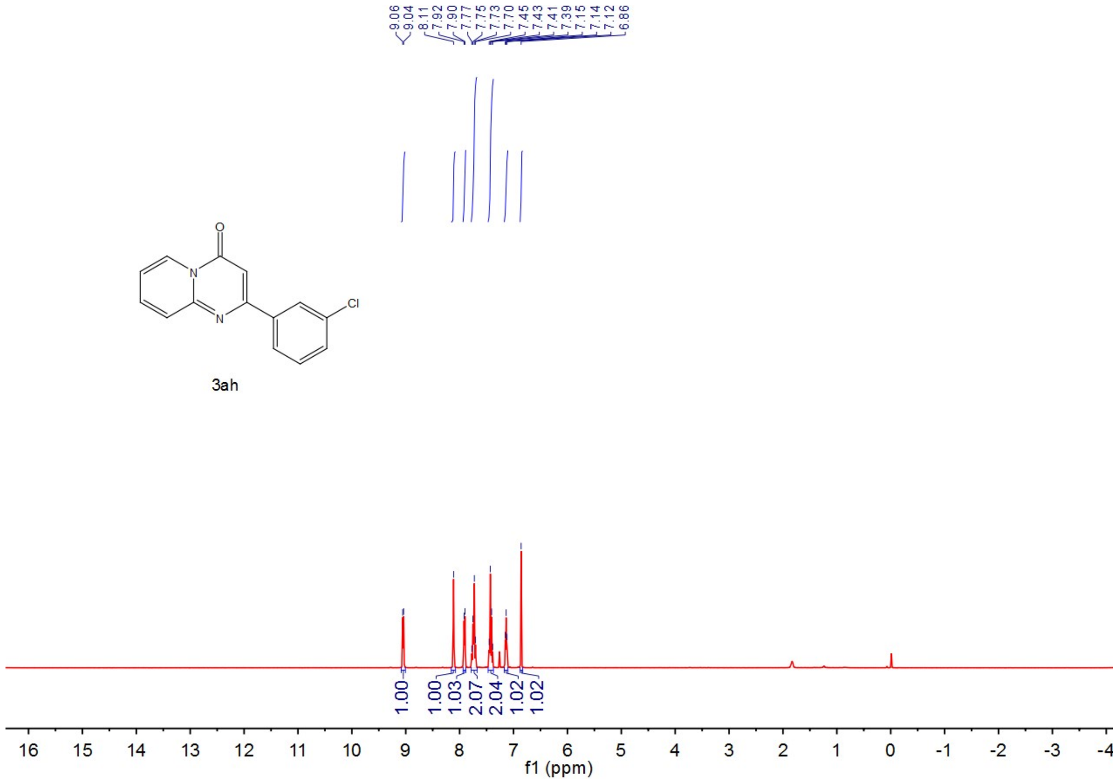

^{13}C spectrum of **3ah**

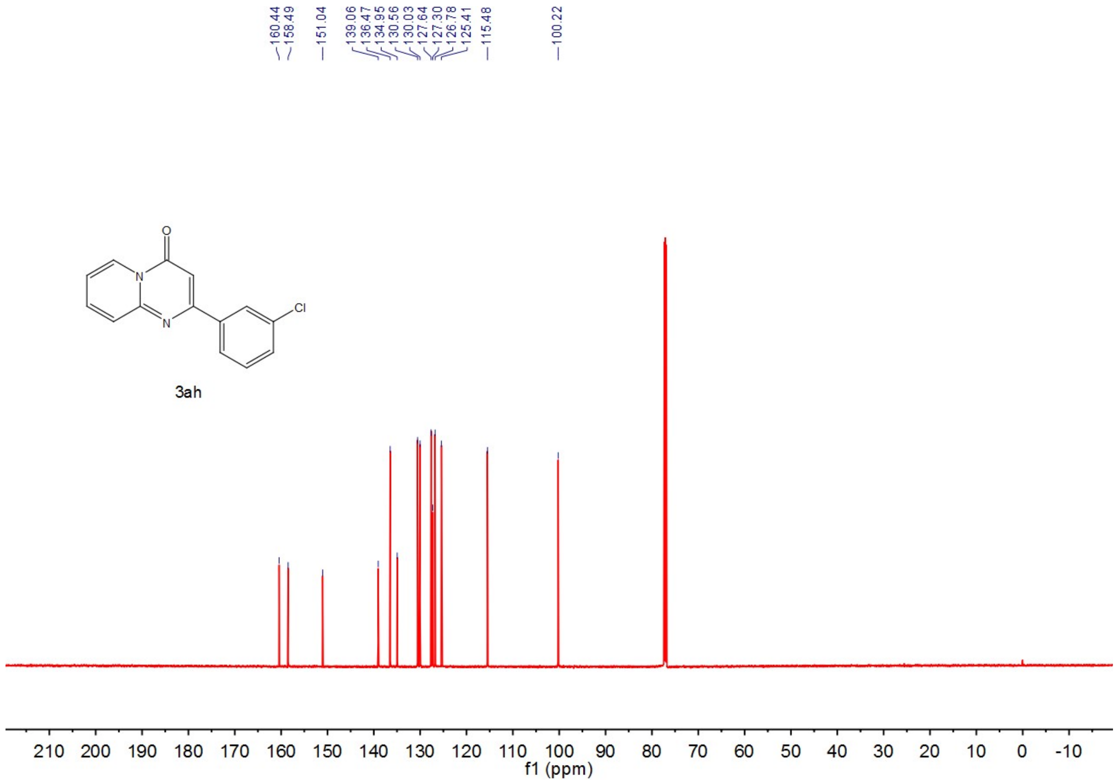

*2-(2-Chlorophenyl)-4H-pyrido[1,2-a]pyrimidin-4-one***(3ai)**

^{1}H spectrum of **3ai**

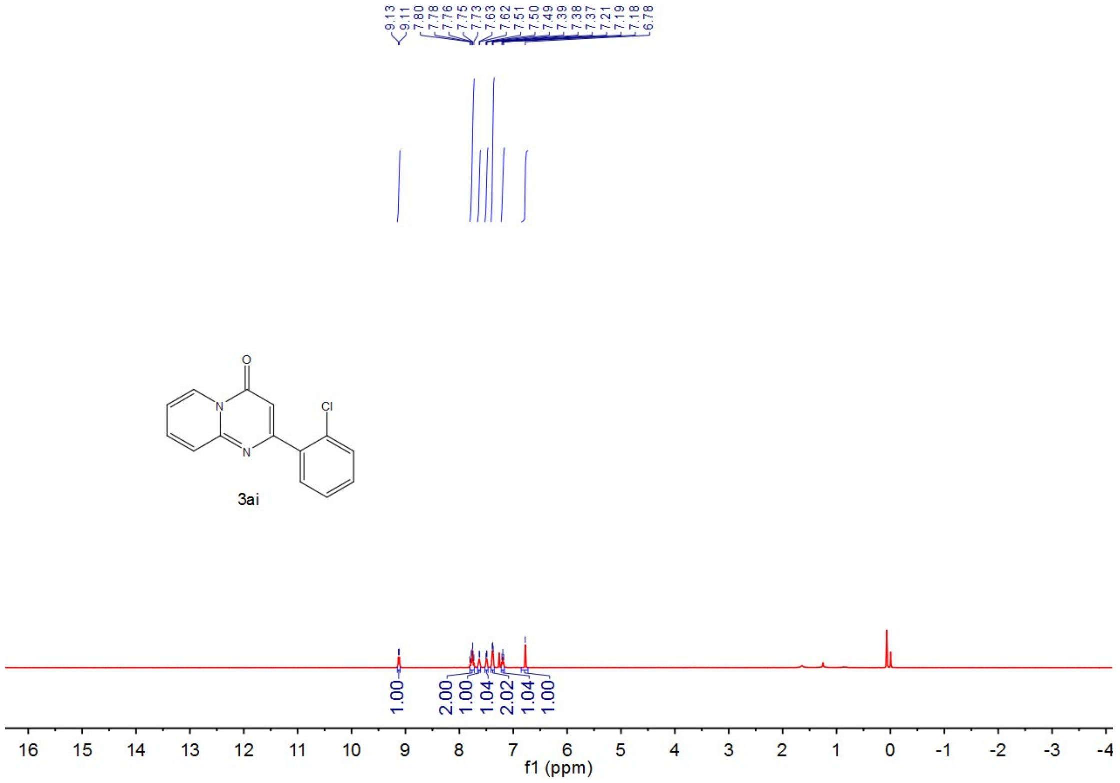

^{13}C spectrum of **3ai**

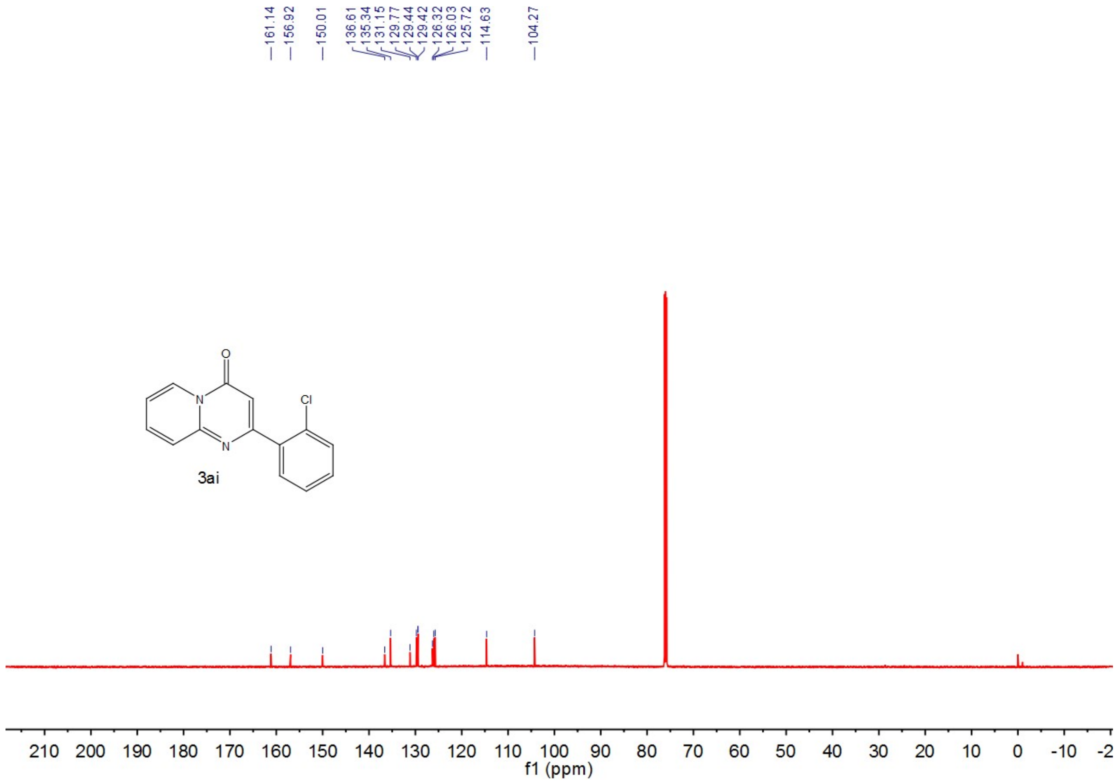

*2-(4-Fluorophenyl)-4H-pyrido[1,2-a]pyrimidin-4-one***(3aj)**

^{1}H spectrum of **3aj**

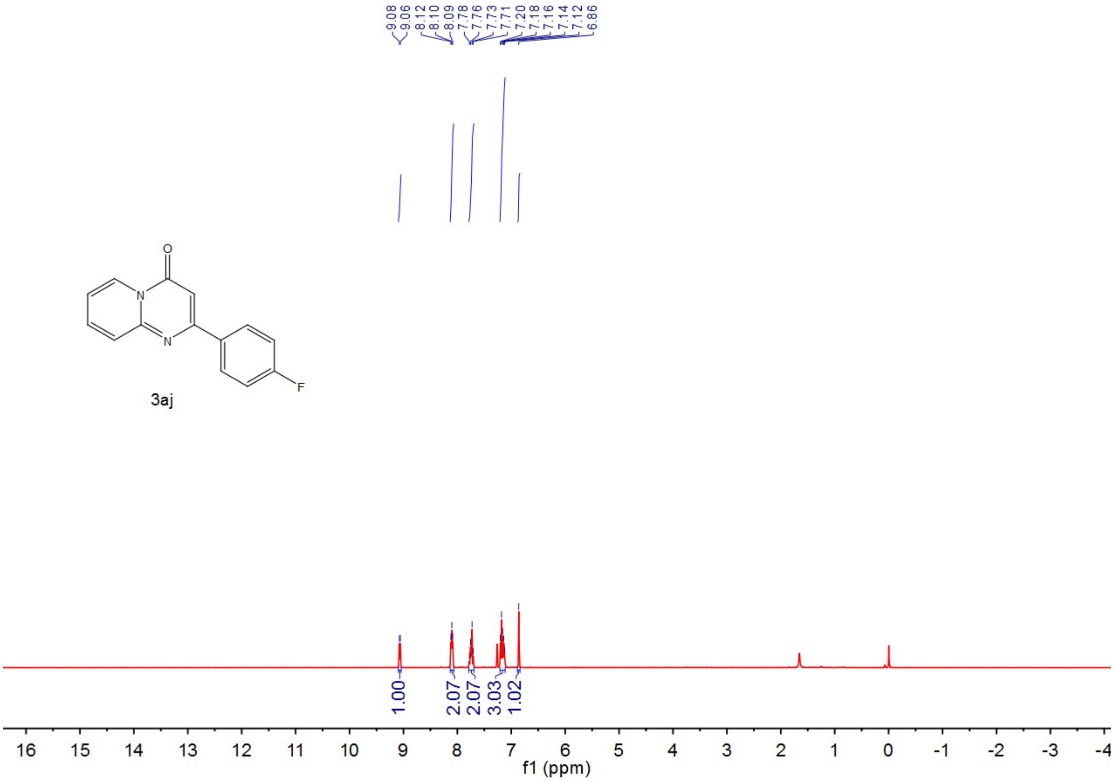

^{13}C spectrum of **3aj**

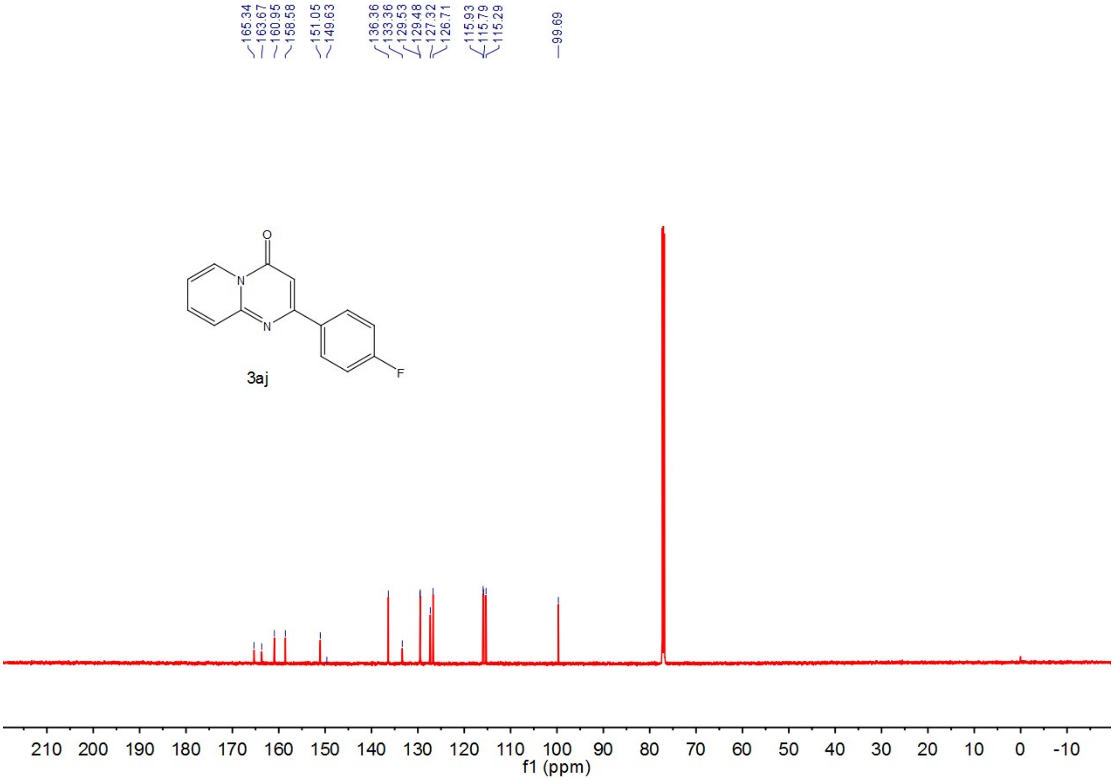

2-(3-Fluorophenyl)-4H-pyrido[1,2-a]pyrimidin-4-one(**3ak**)

^{1}H spectrum of **3ak**

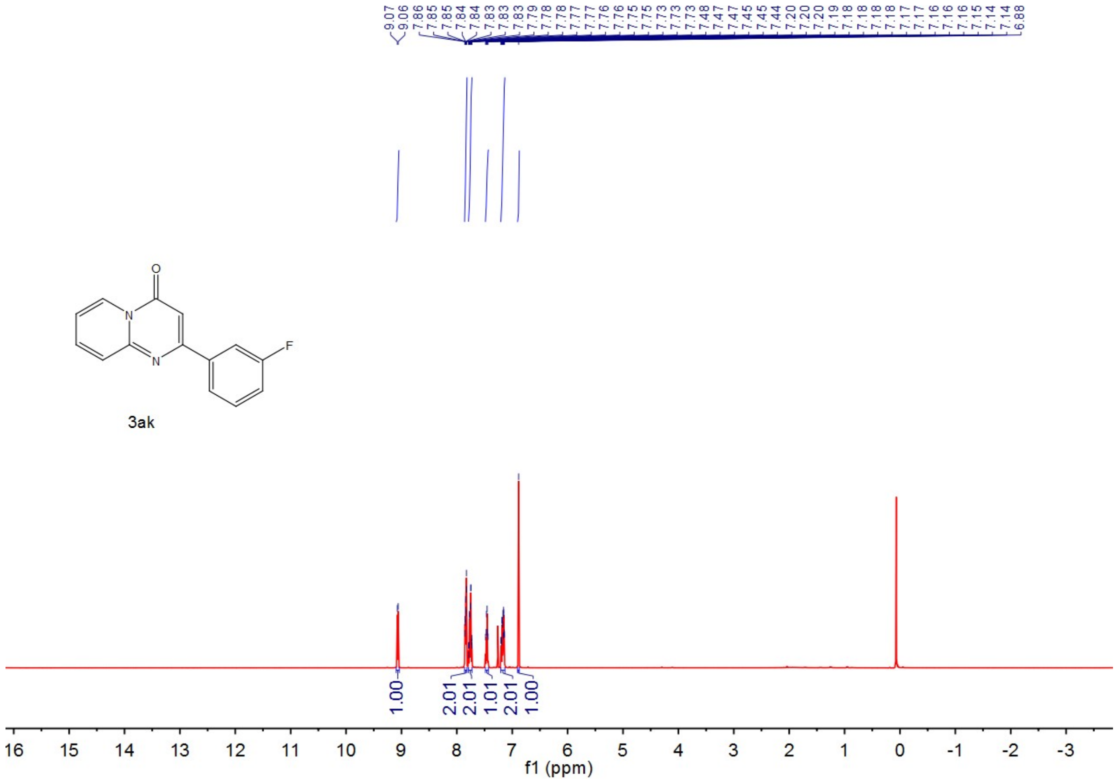

^{13}C spectrum of **3ak**

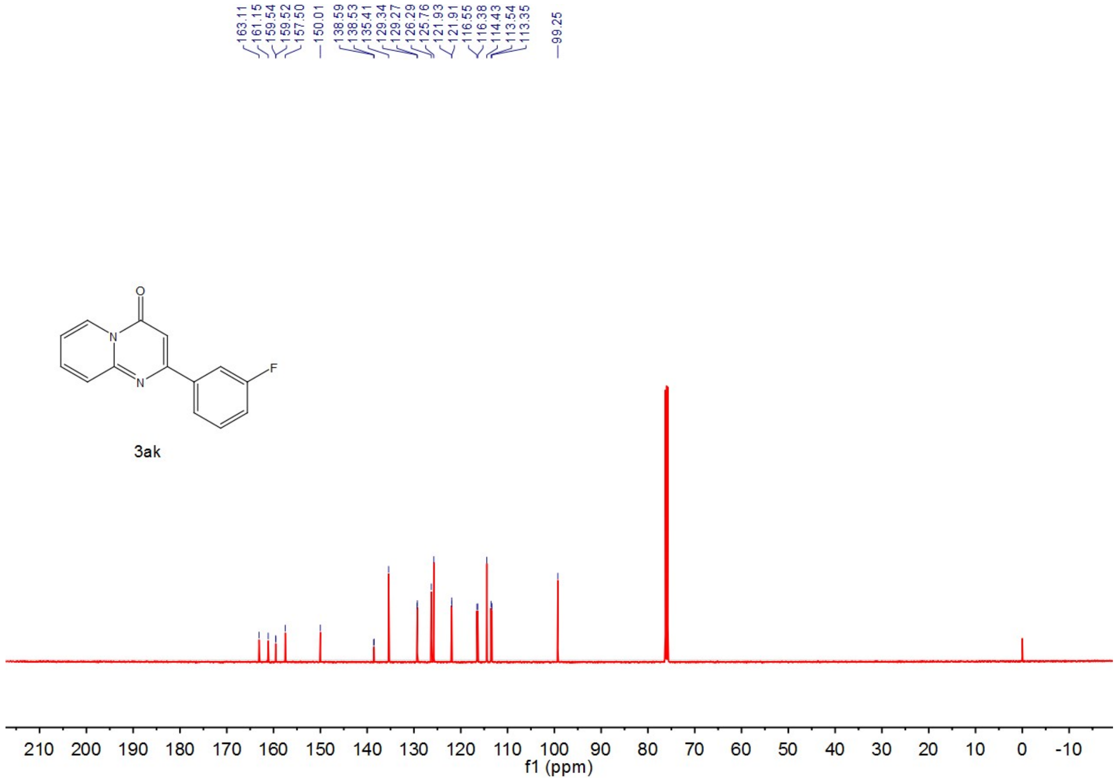

2-(2-Fluorophenyl)-4H-pyrido[1,2-a]pyrimidin-4-one(**3al)**

^{1}H spectrum of **3al**

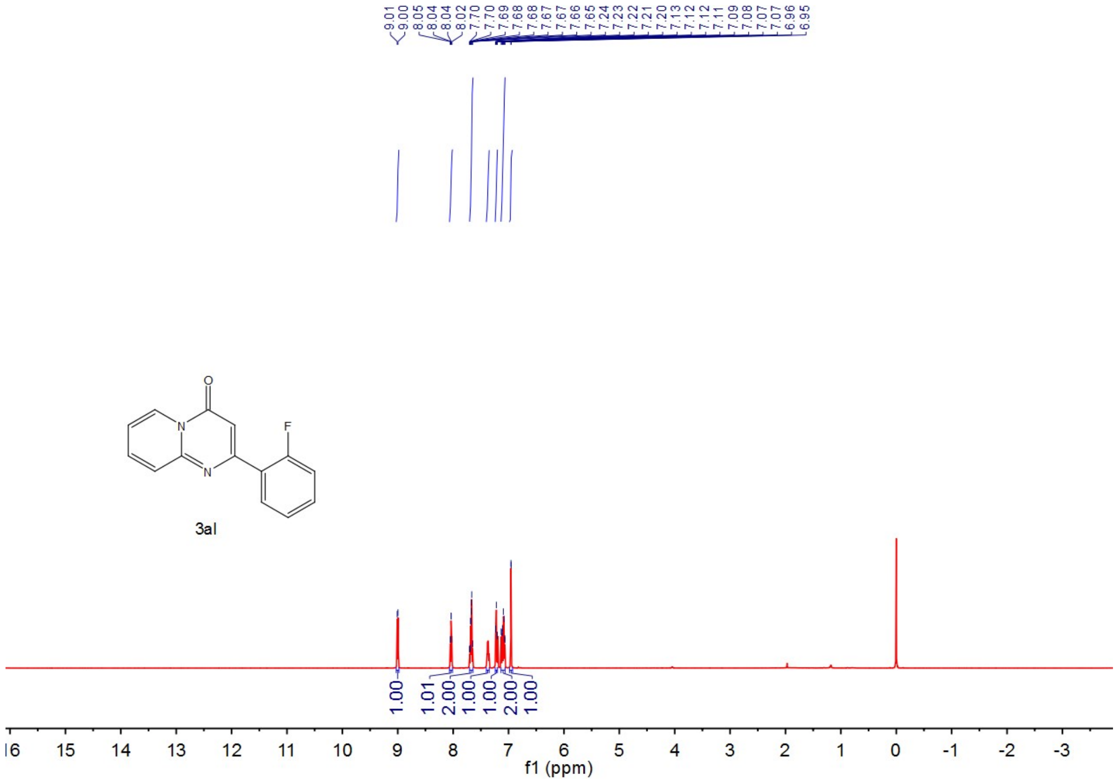

^{13}C spectrum of **3al**

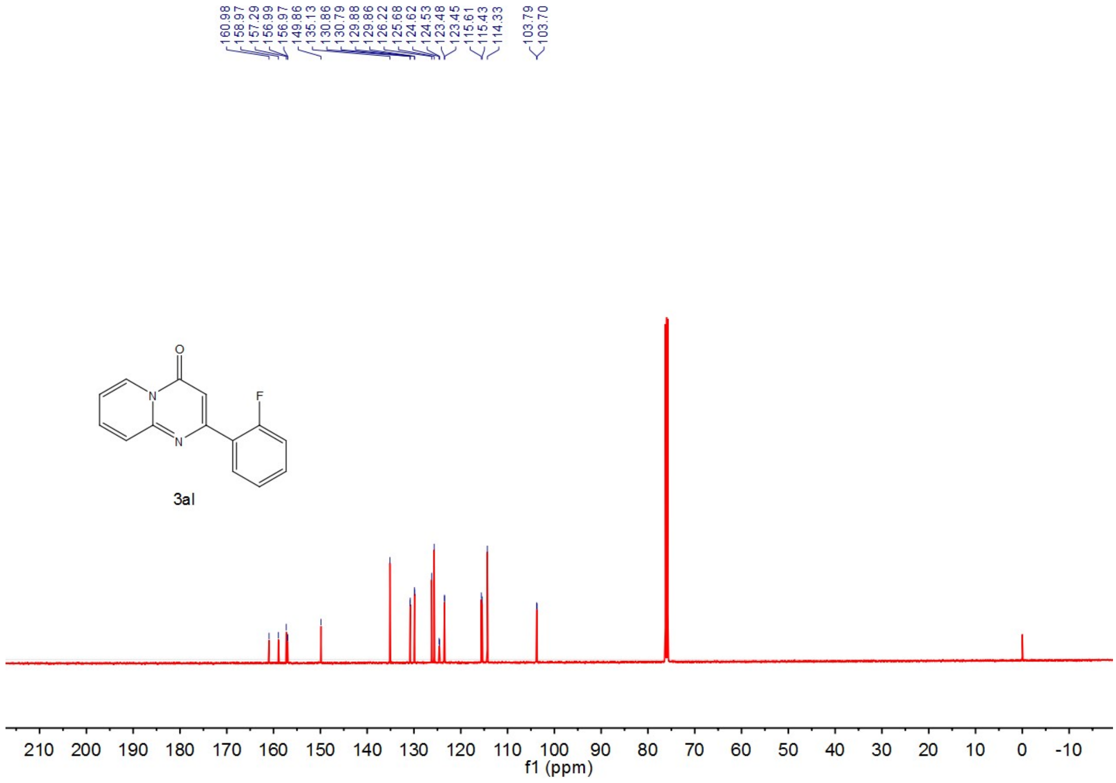

*2-(4-Bromophenyl)-4H-pyrido[1,2-a]pyrimidin-4-one***(3am)**

^{1}H spectrum of **3am**

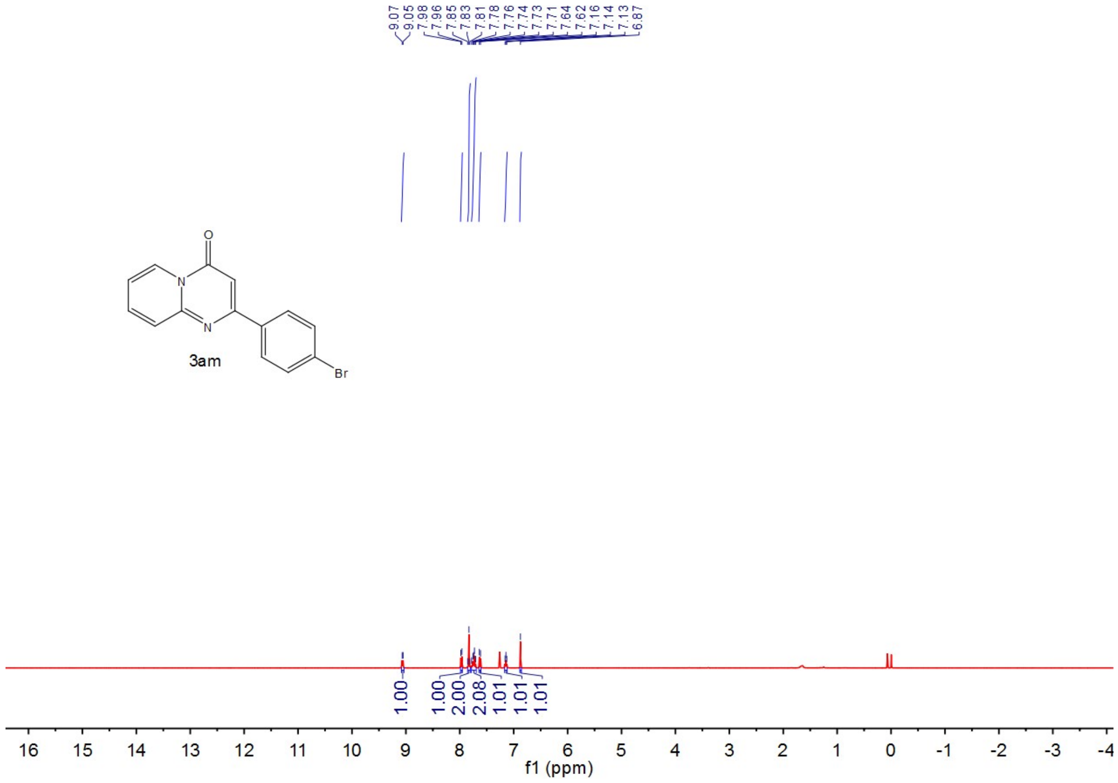

^{13}C spectrum of **3am**

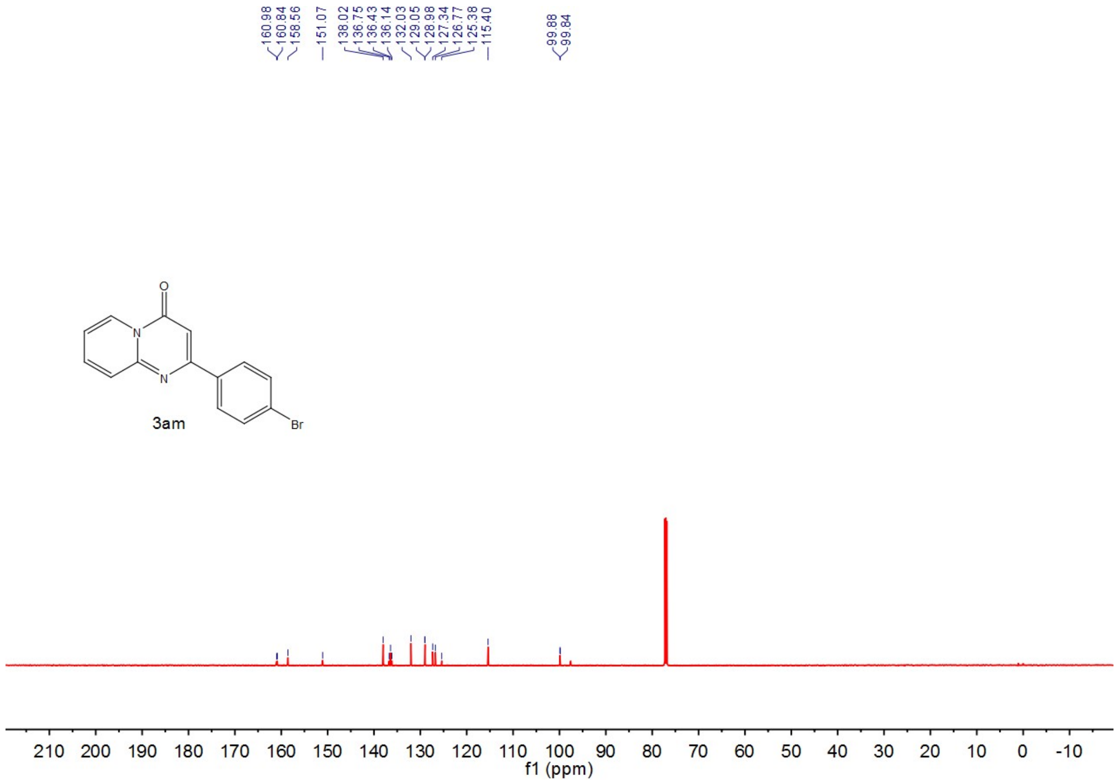

*2-(4-(Trifluoromethyl)phenyl)-4H-pyrido[1,2-a]pyrimidin-4-one***(3an)**

^{1}H spectrum of **3an**

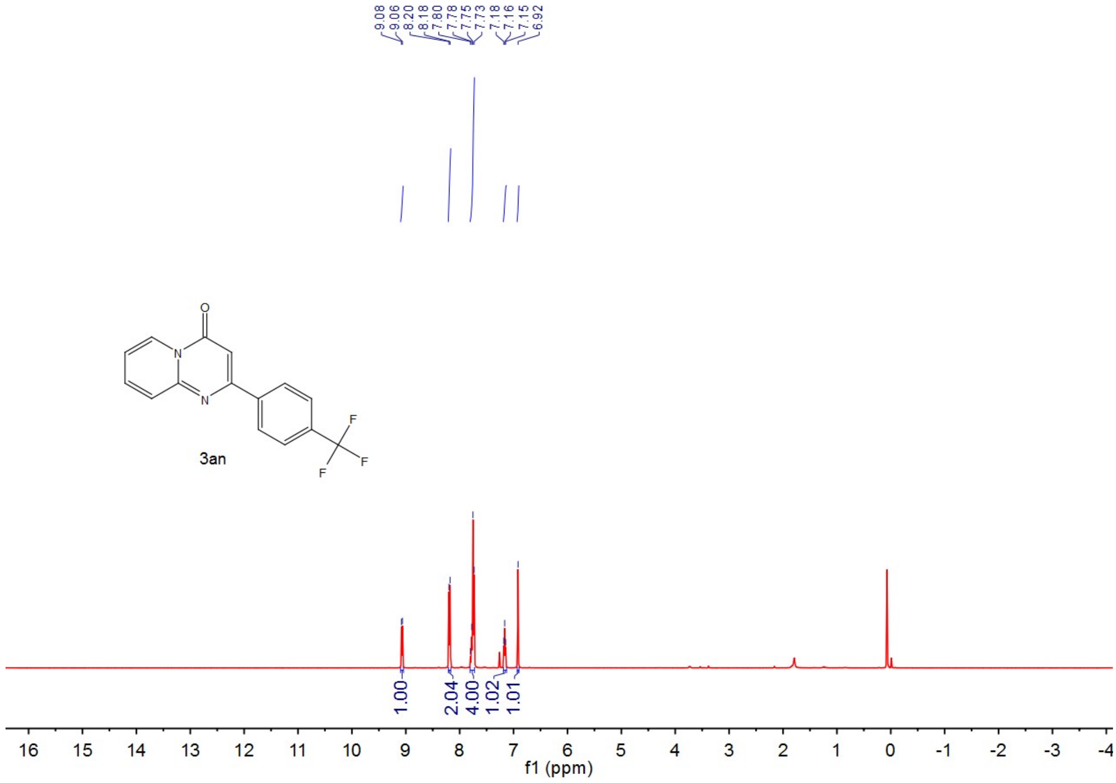

^{13}C spectrum of **3an**

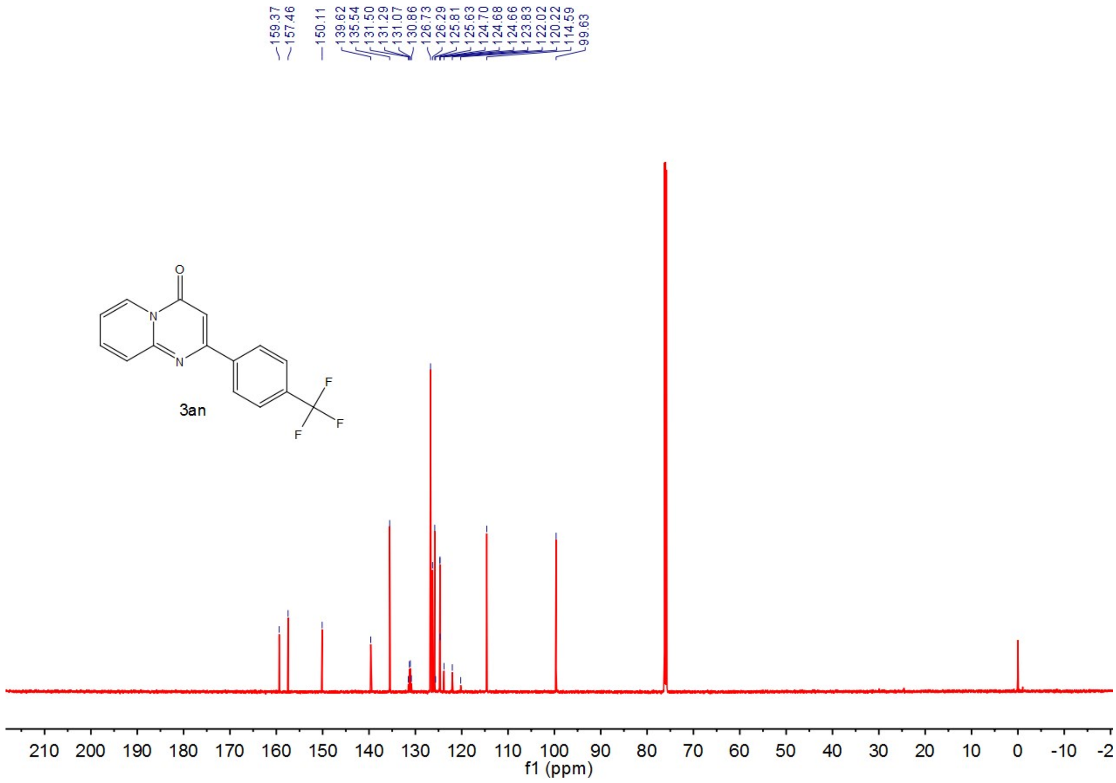

*2-(3-(Trifluoromethyl)phenyl)-4H-pyrido[1,2-a]pyrimidin-4-one***(3ao)**

^{1}H spectrum of **3ao**

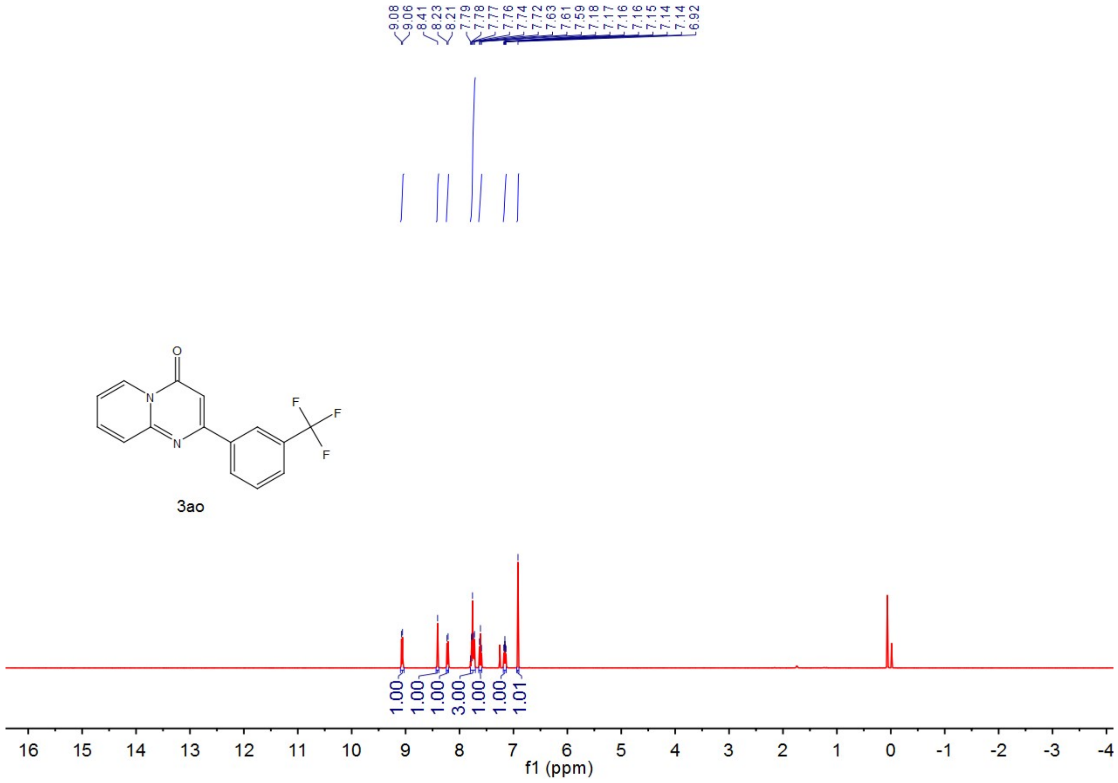

^{13}C spectrum of **3ao**

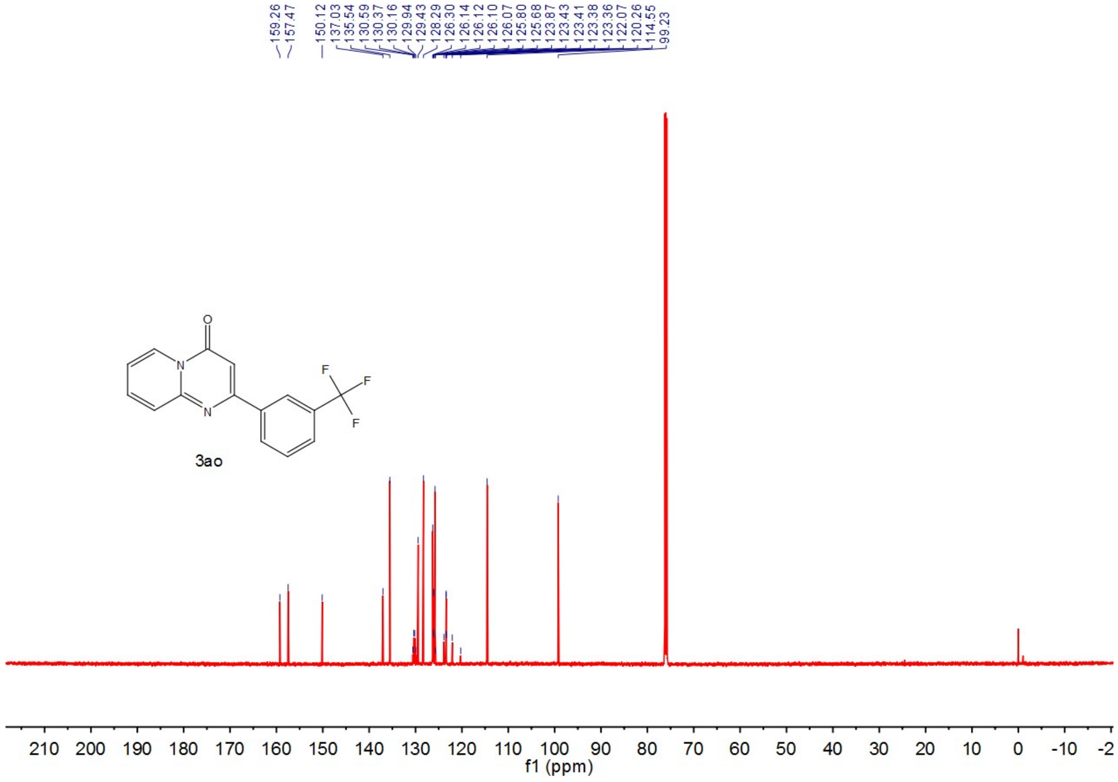

2-Methyl-4H-pyrido[1,2-a]pyrimidin-4-one(**3ap)**

^{1}H spectrum of **3ap**

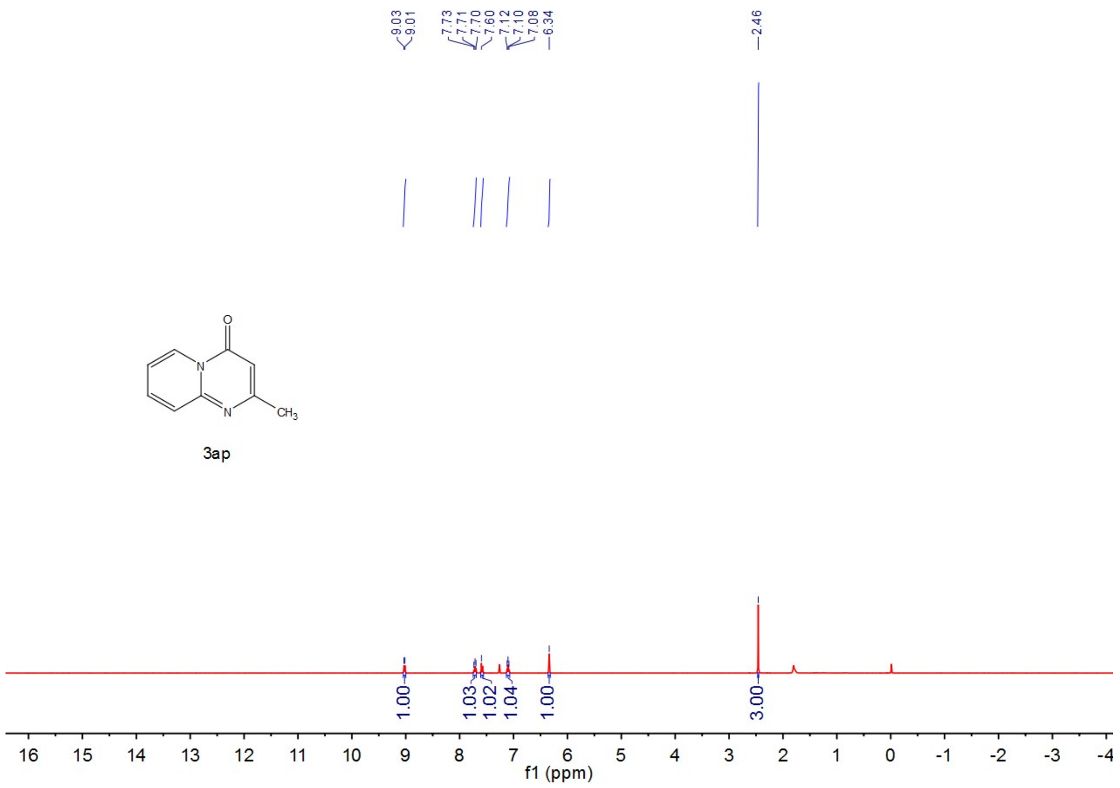

^{13}C spectrum of **3ap**

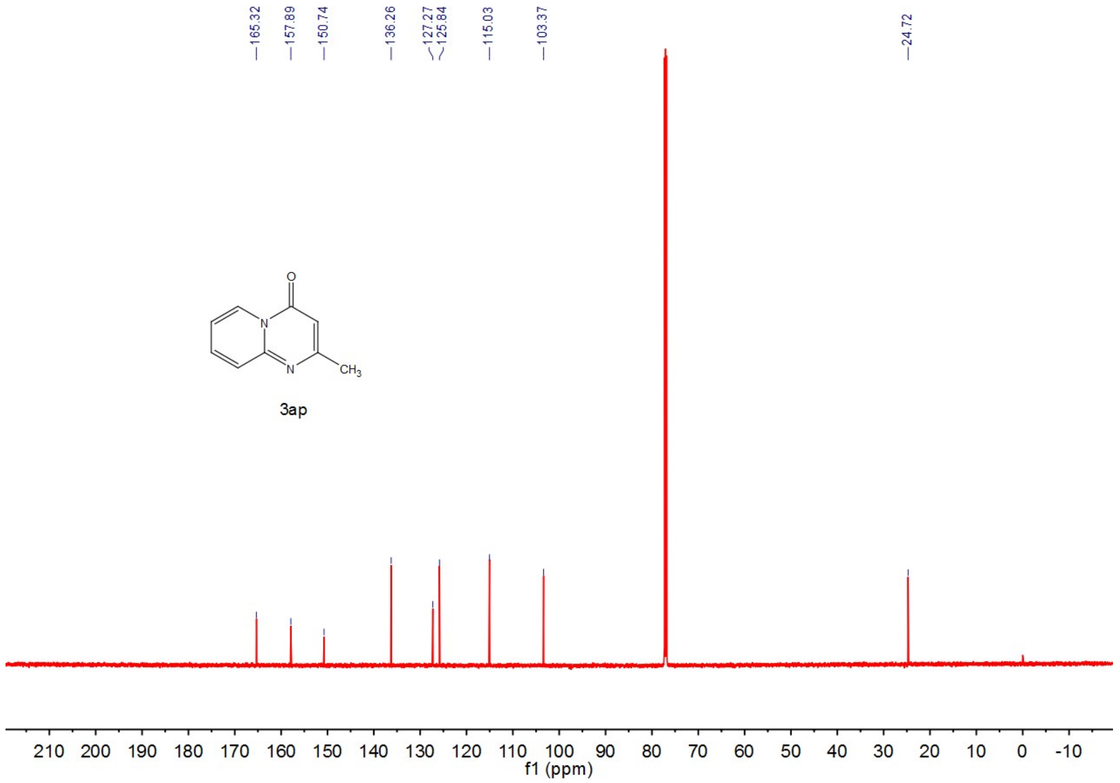

2-Phenyl-4H-pyrido[1,2-a]pyrimidin-4-one(**3ba**)

^{1}H spectrum of **3ba**

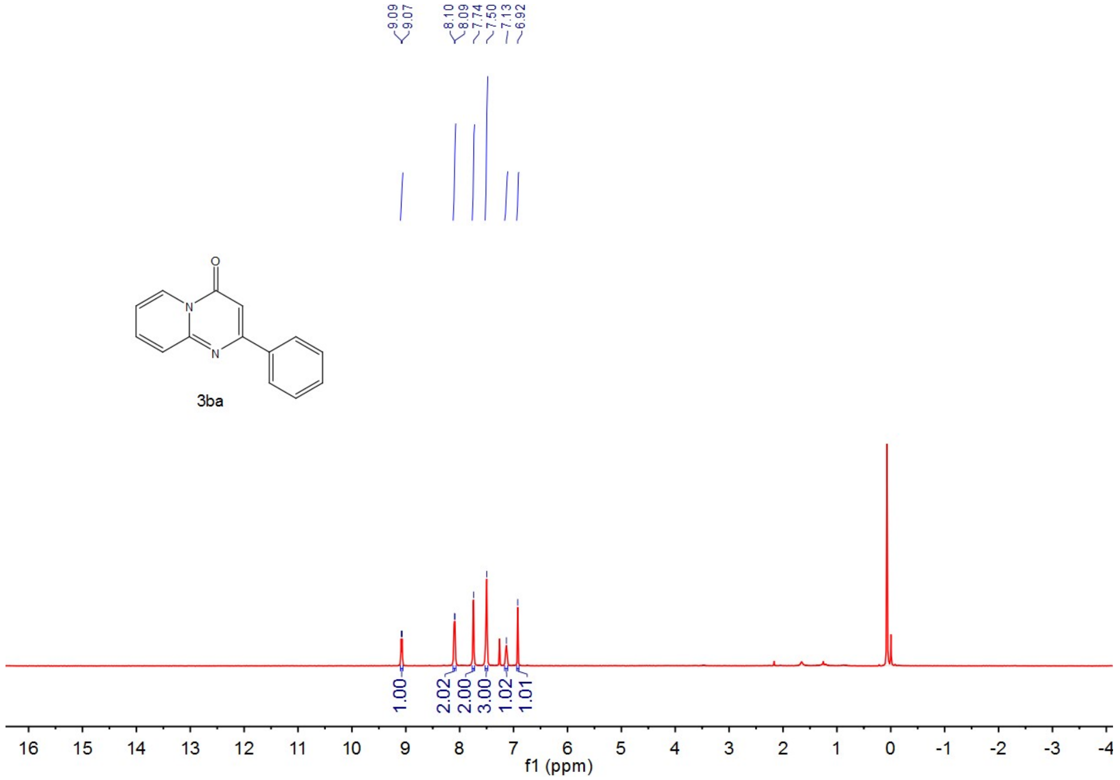

^{13}C spectrum of **3ba**

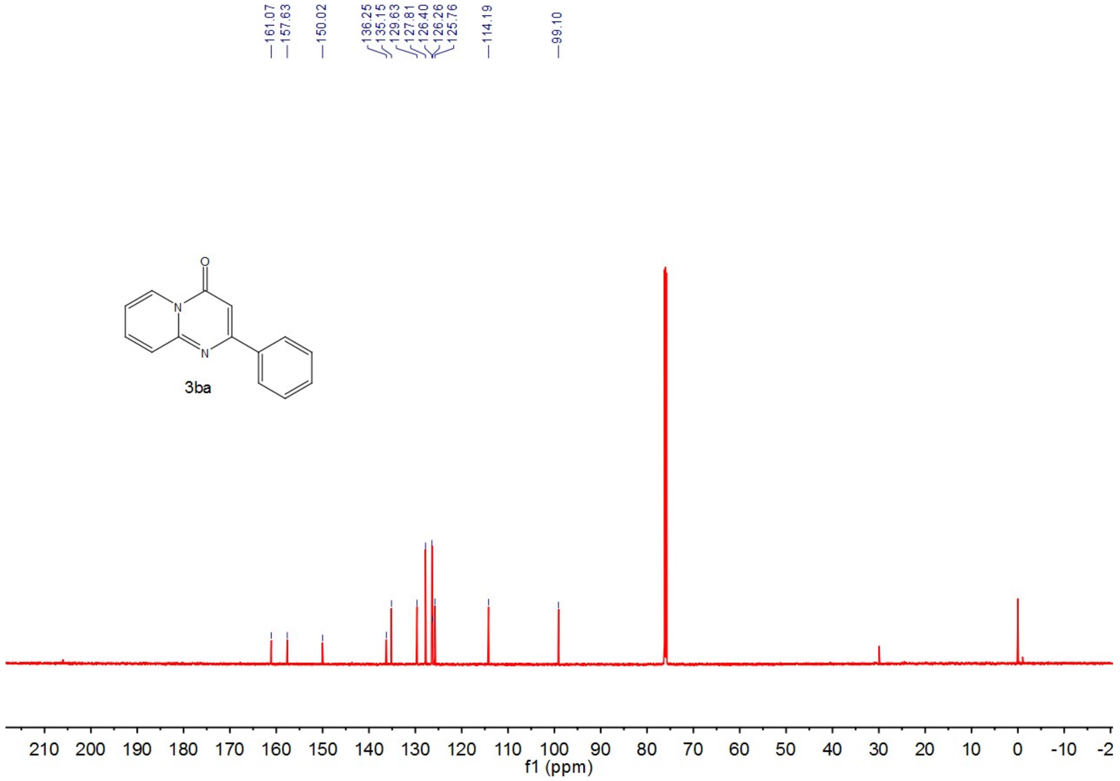

9-Methyl-2-phenyl-4H-pyrido[1,2-a]pyrimidin-4-one(**3ca**)

^{1}H spectrum of **3ca**

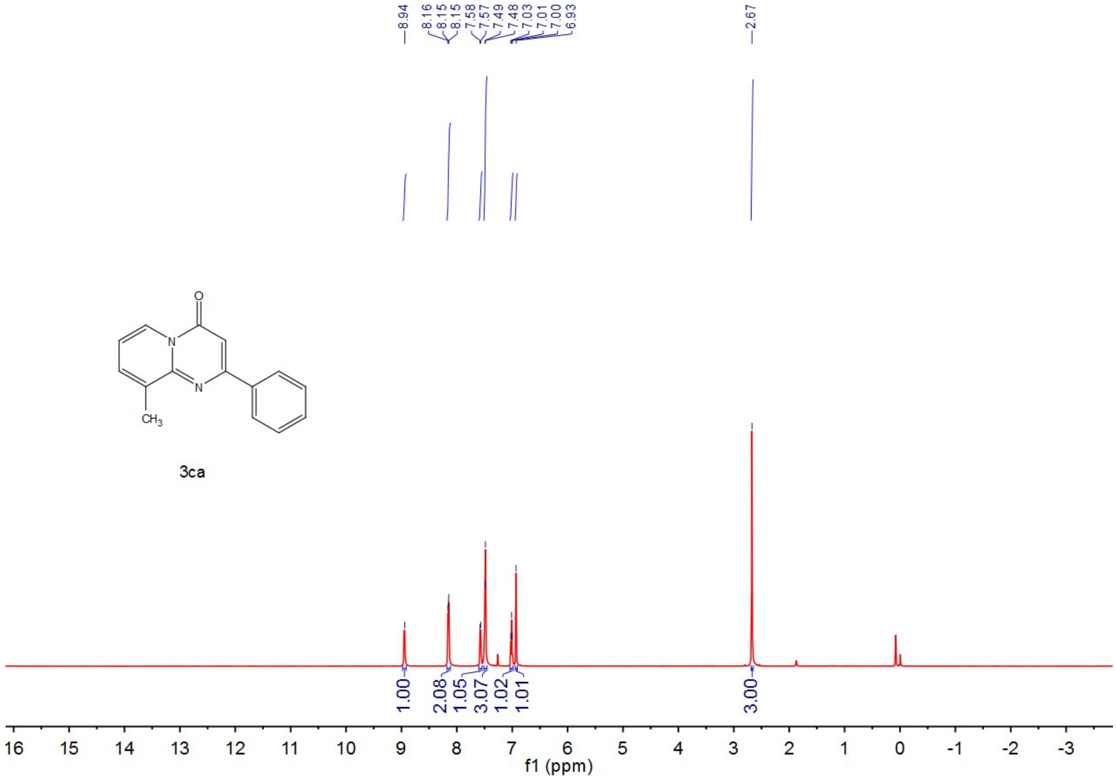

^{13}C spectrum of **3ca**

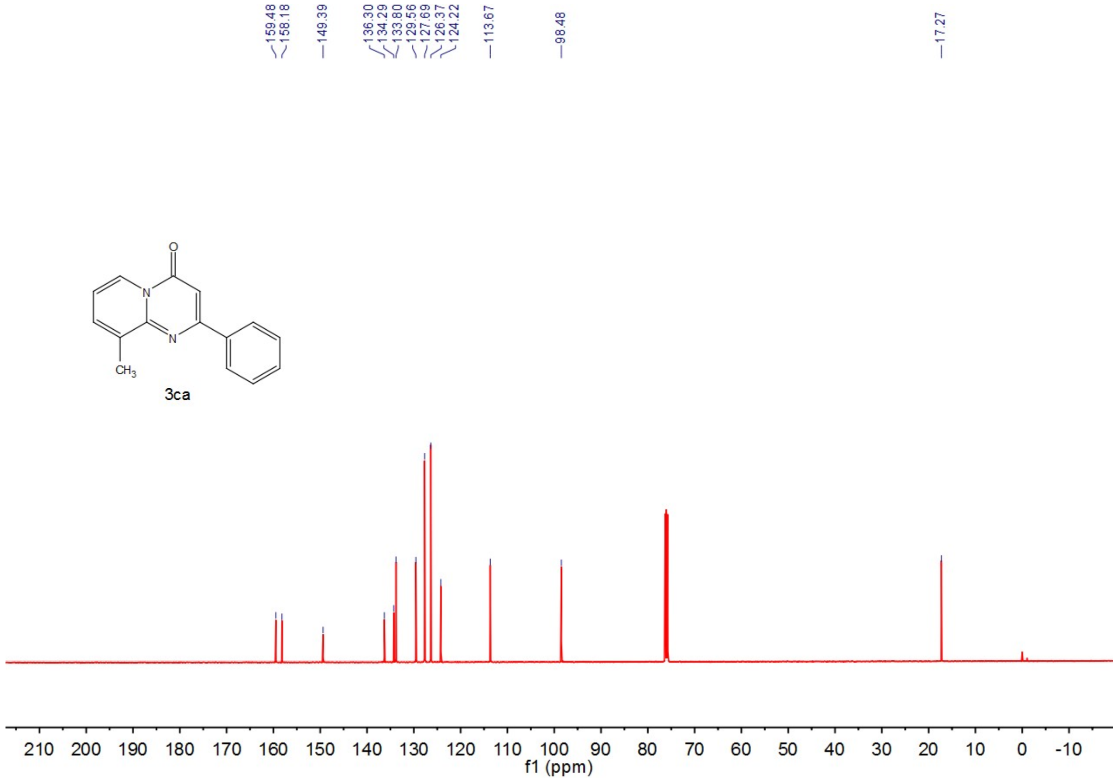

*8-Methyl-2-phenyl-4H-pyrido[1,2-a]pyrimidin-4-one***(3da)**

^{1}H spectrum of **3da**

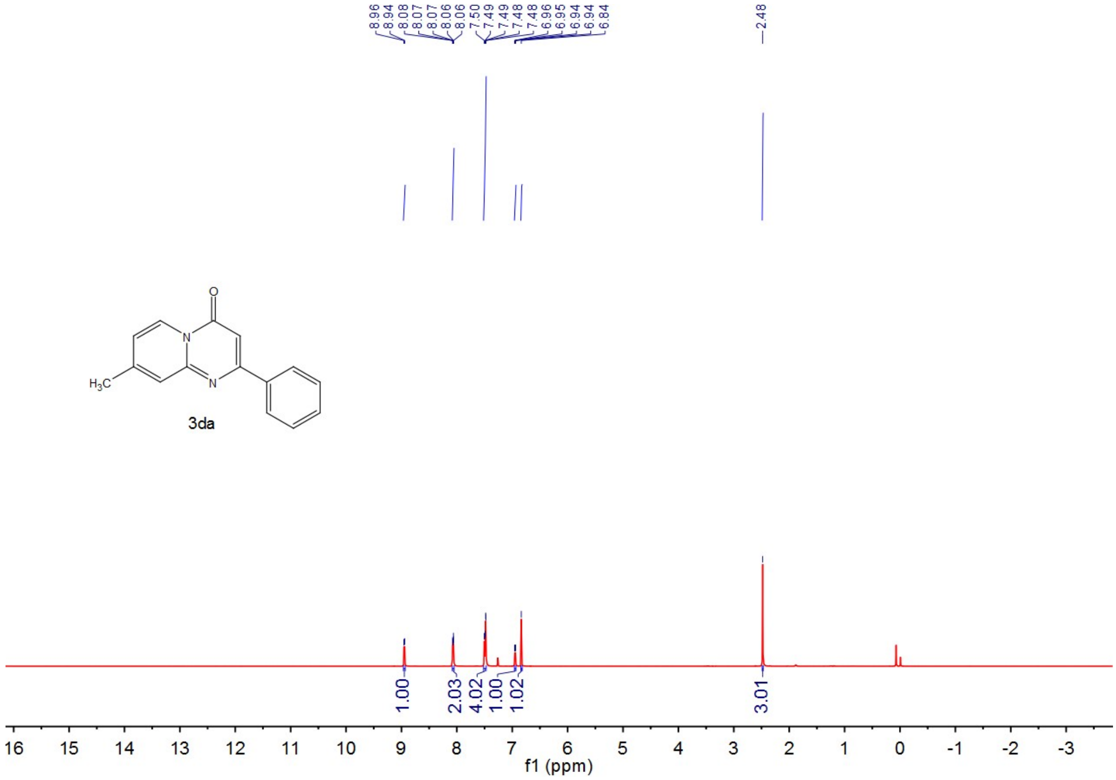

^{13}C spectrum of **3da**

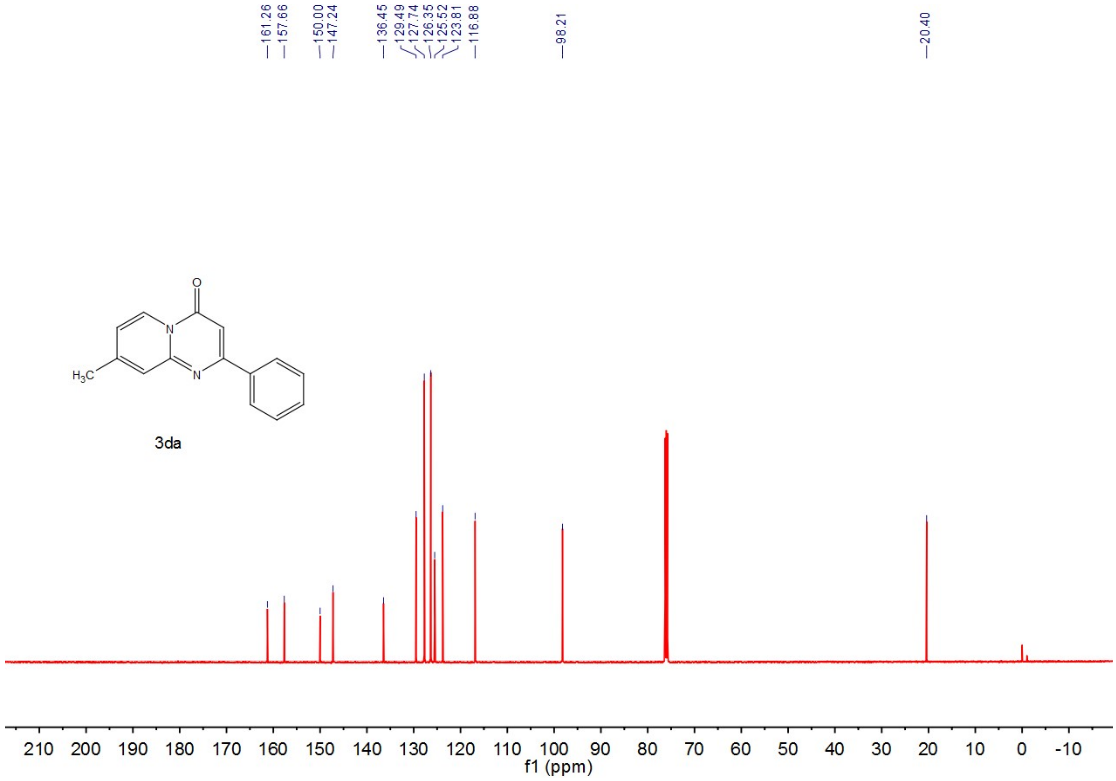

*7-Methyl-2-phenyl-4H-pyrido[1,2-a]pyrimidin-4-one***(3ea)**

^{1}H spectrum of **3ea**

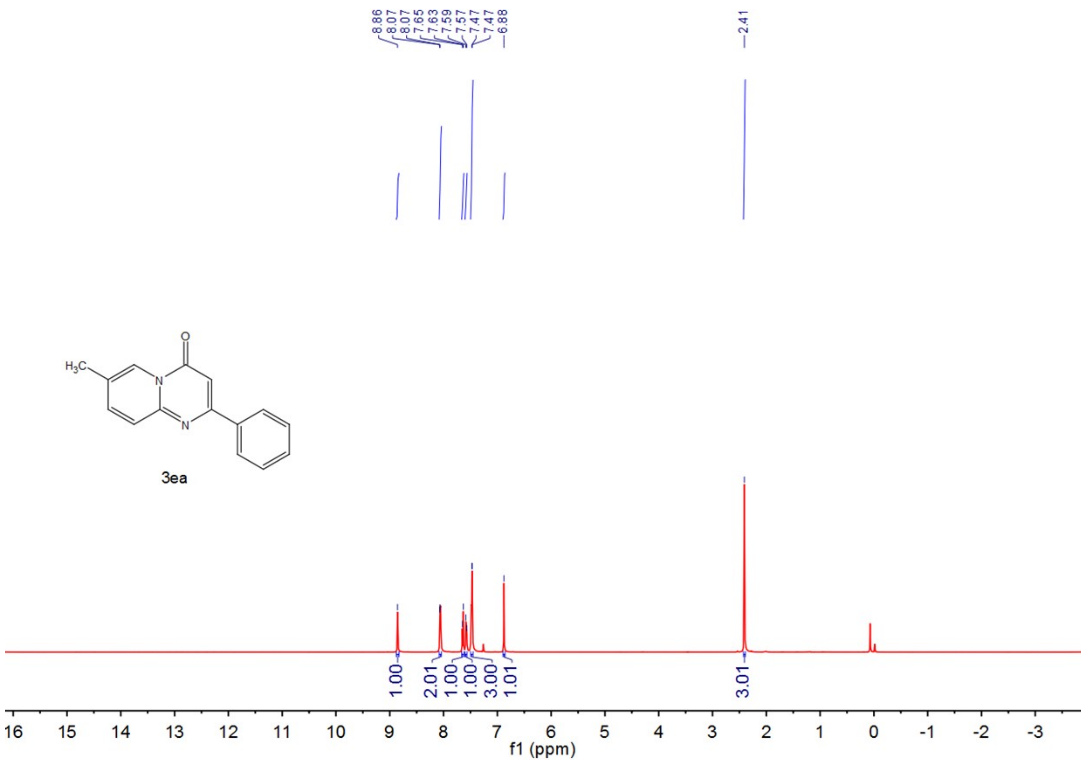

^{13}C spectrum of **3ea**

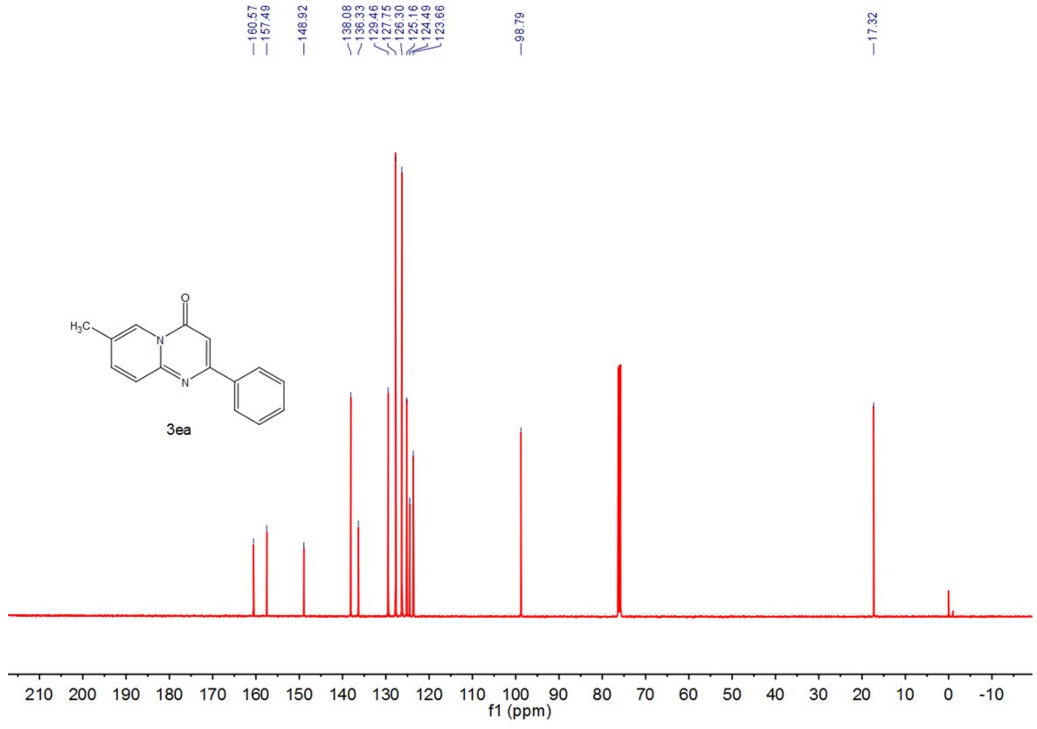

*8-Methoxy-2-phenyl-4H-pyrido[1,2-a]pyrimidin-4-one***(3fa)**

^{1}H spectrum of **3fa**

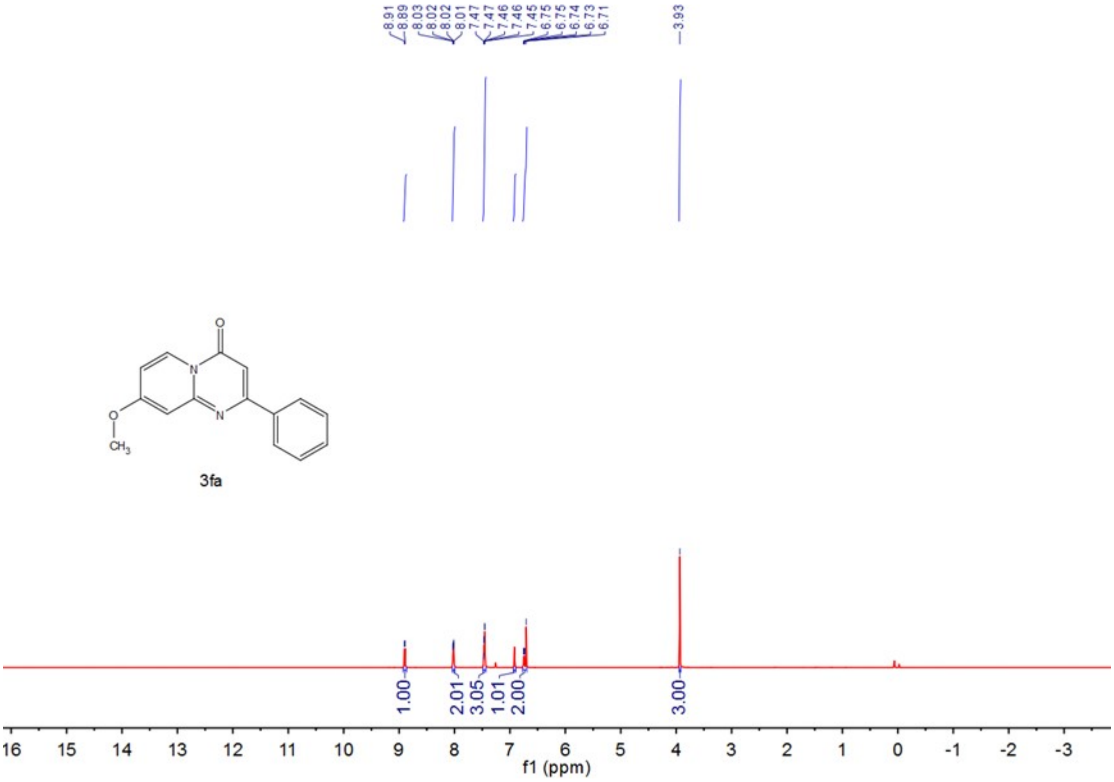

^{13}C spectrum of **3fa**

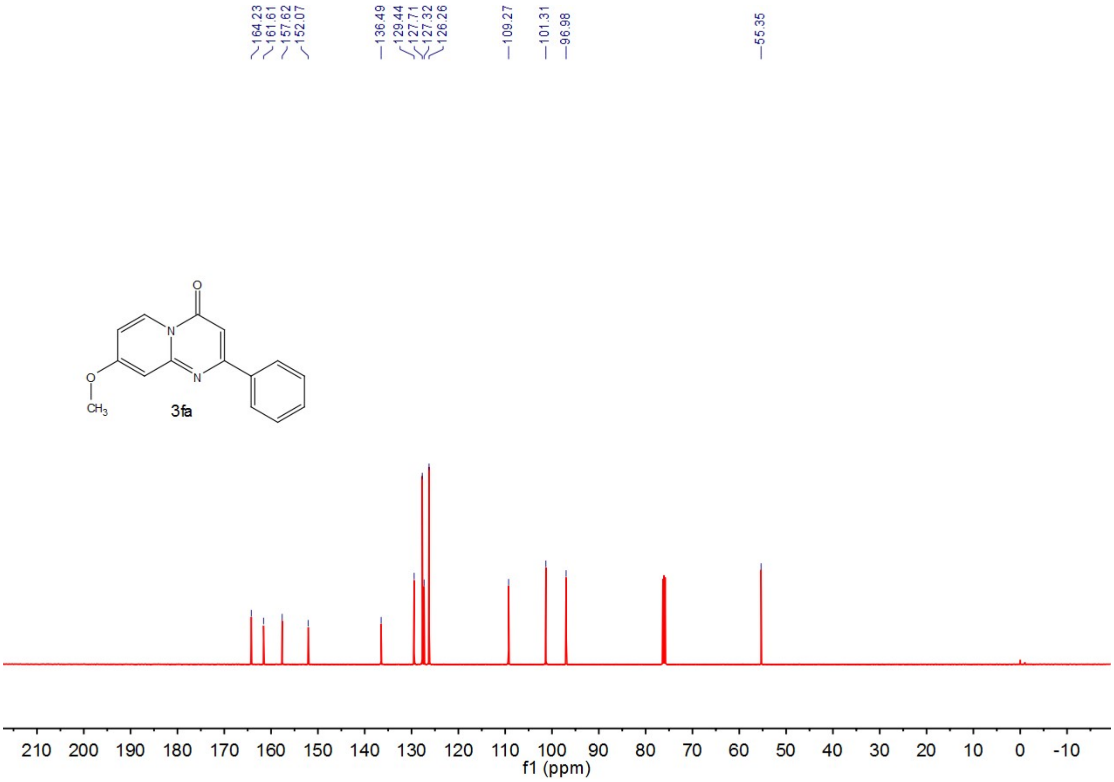

*9-Methoxy-2-phenyl-4H-pyrido[1,2-a]pyrimidin-4-one***(3ga)**

^{1}H spectrum of **3ga**

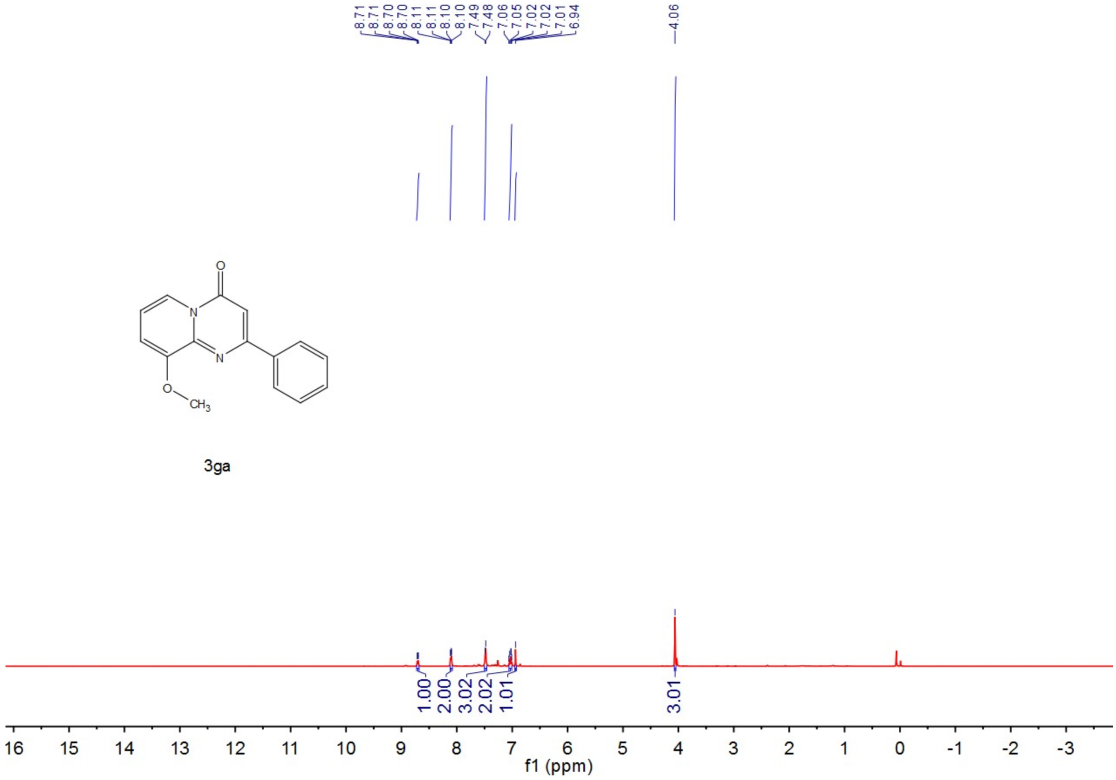

^{13}C spectrum of **3ga**

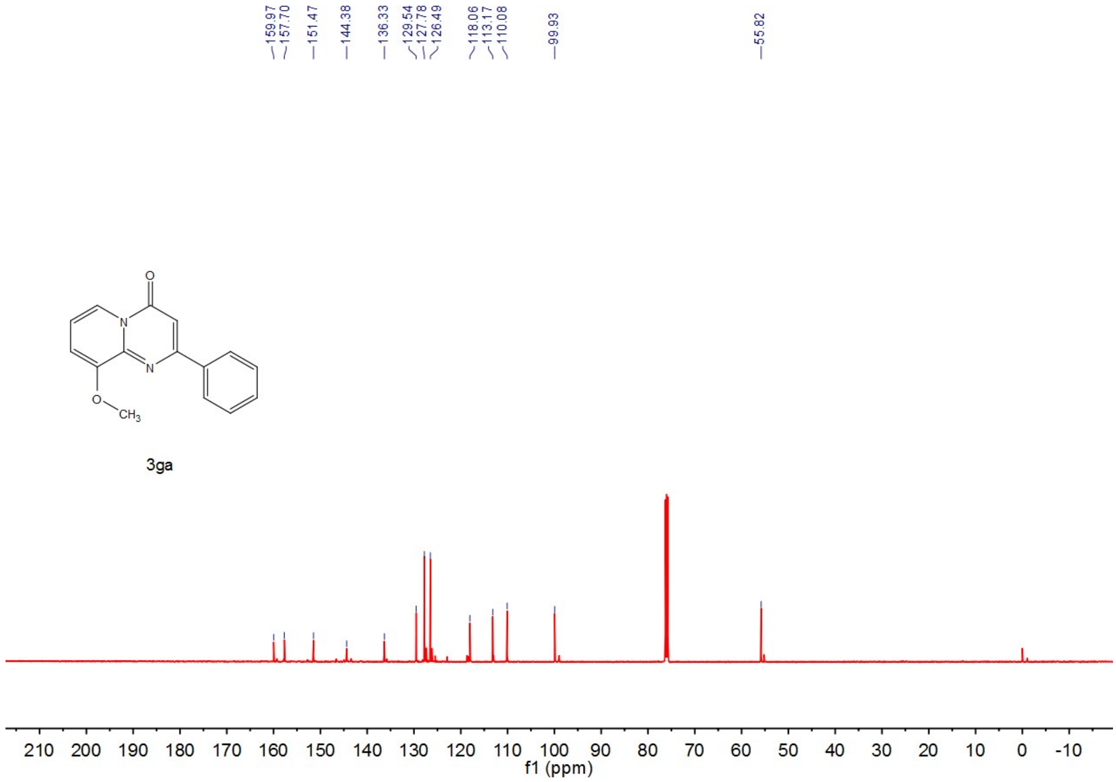

*7-Methoxy-2-phenyl-4H-pyrido[1,2-a]pyrimidin-4-one***(3ha)**

^{1}H spectrum of **3ha**

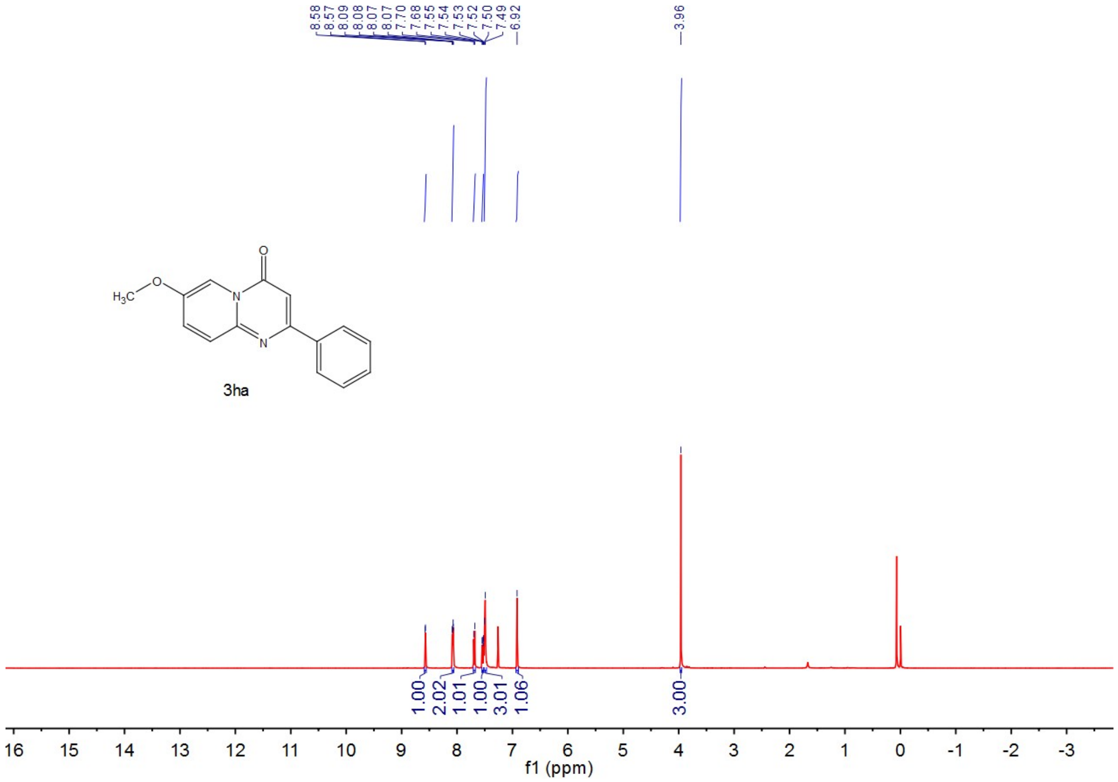

^{13}C spectrum of **3ha**

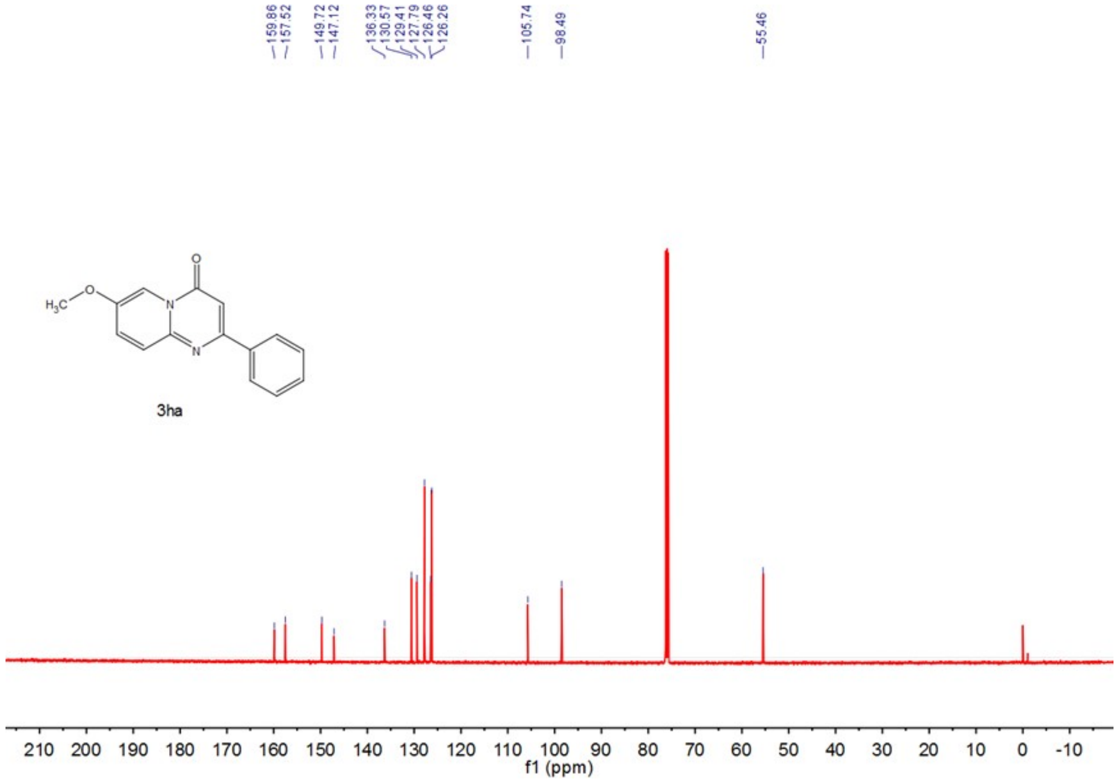

7-Chloro-2-phenyl-4H-pyrido[1,2-a]pyrimidin-4-one(**3ia**)

^{1}H spectrum of **3ia**

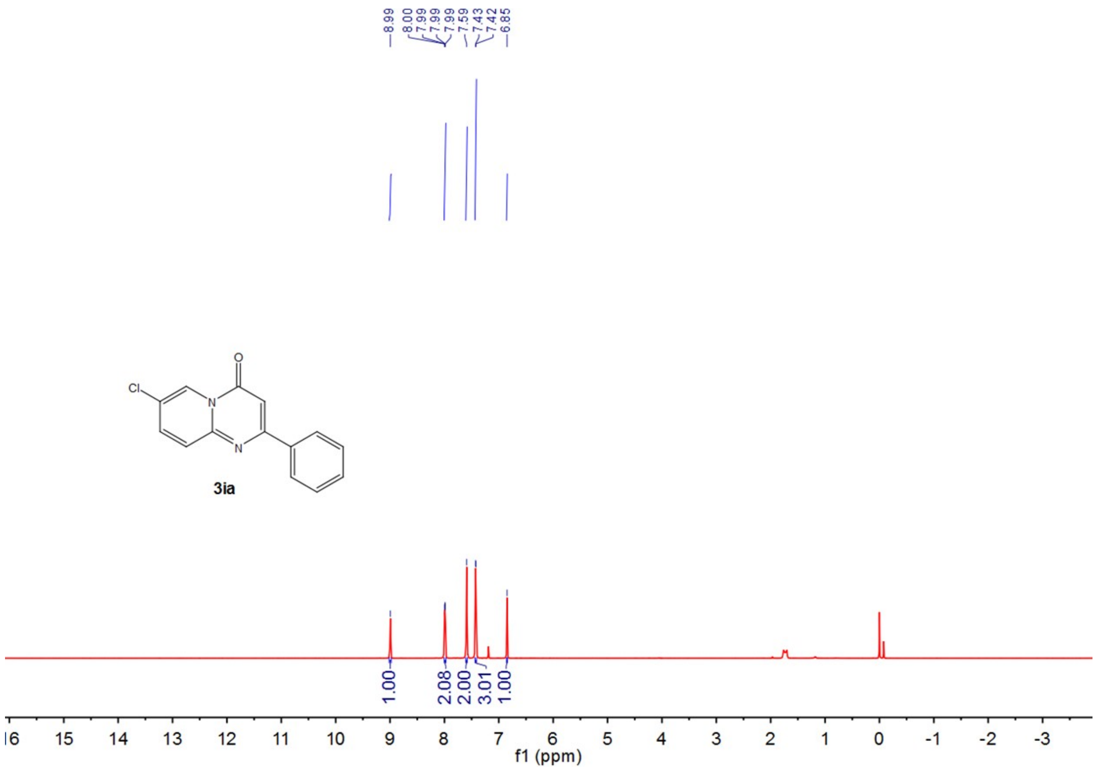

^{13}C spectrum of **3ia**

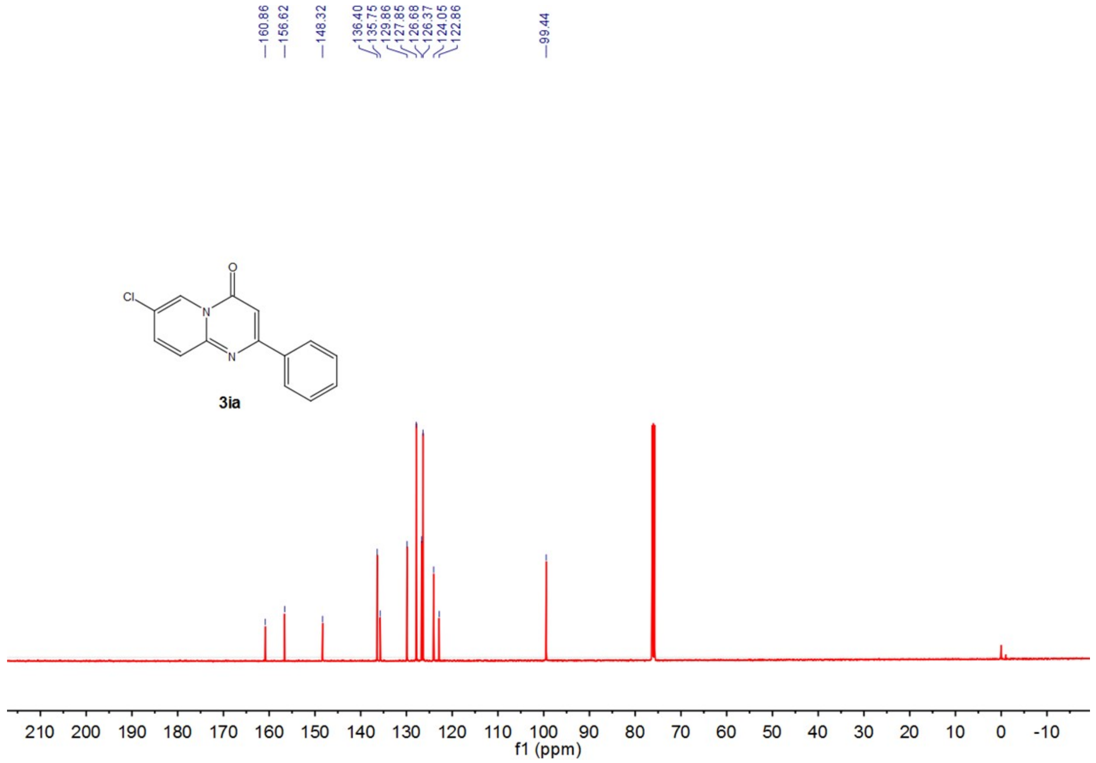

3-Phenyl-1H-pyrimido[1,2-a]quinolin-1-one(**3ja**)

^{1}H spectrum of **3ja**

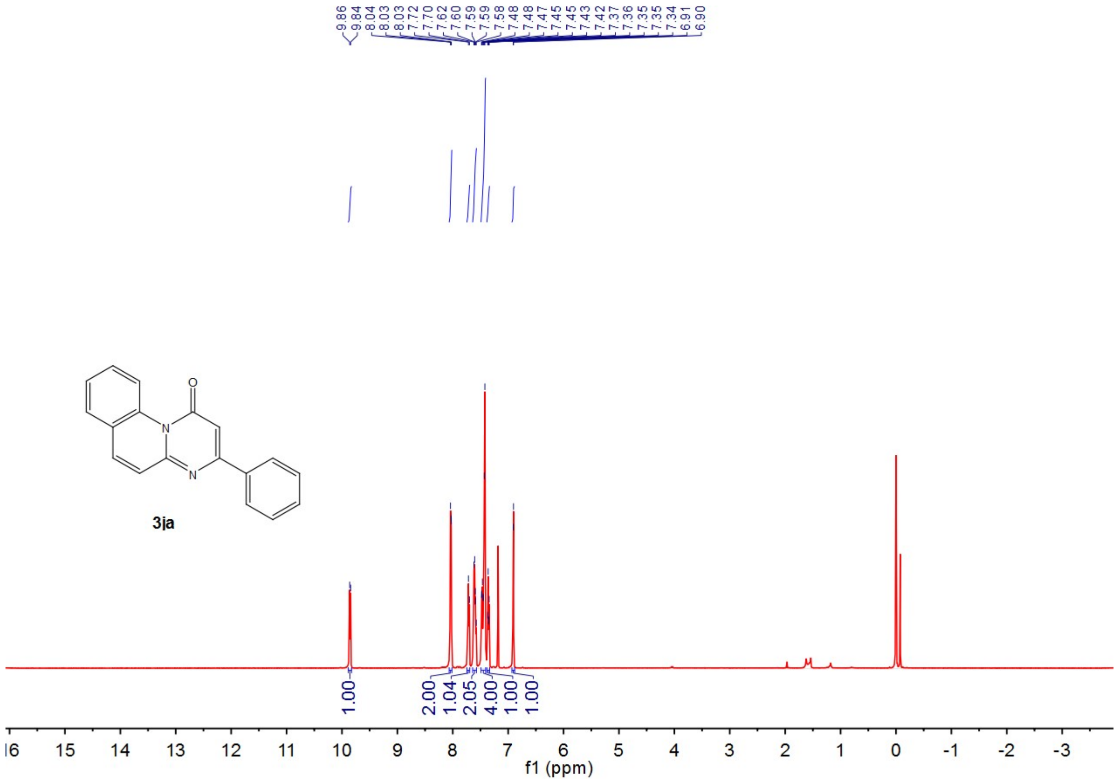

^{13}C spectrum of **3ja**

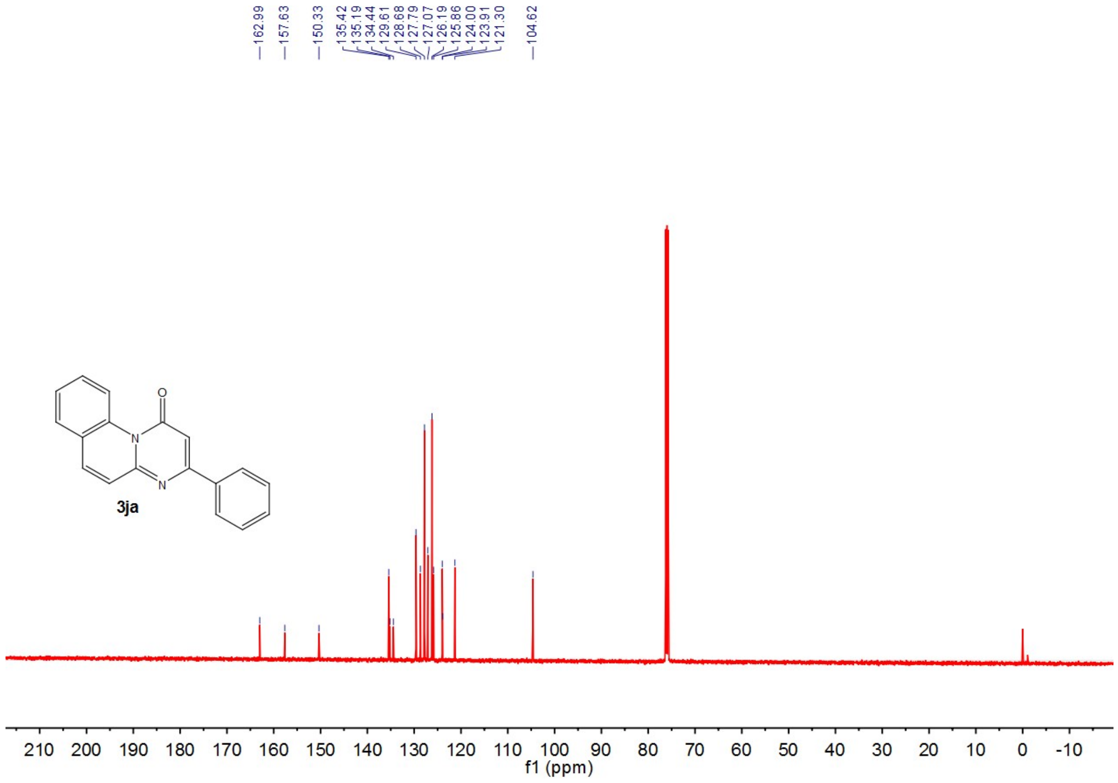

2. High-resolution mass spectra of new compounds

*2-(3-Fluorophenyl)-4H-pyrido[1,2-a]pyrimidin-4-one***(3ak)**

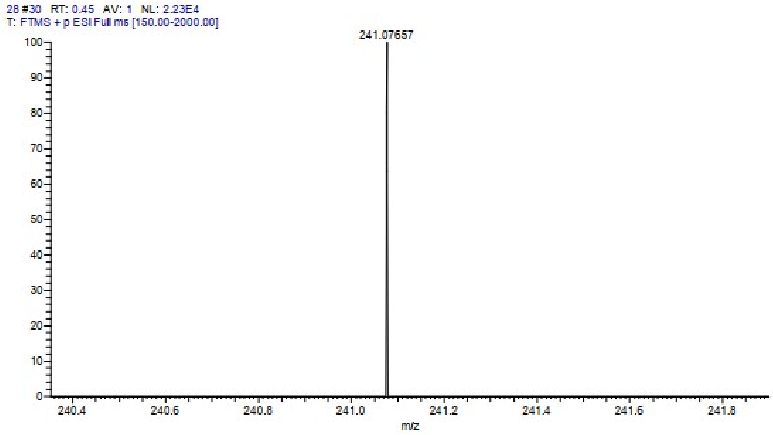

*2-(2-Fluorophenyl)-4H-pyrido[1,2-a]pyrimidin-4-one***(3al)**

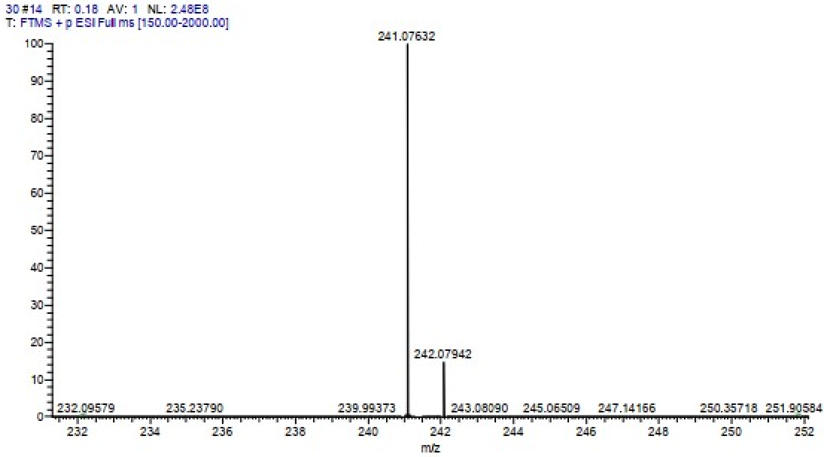

*2-(3-(Trifluoromethyl)phenyl)-4H-pyrido[1,2-a]pyrimidin-4-one***(3ao)**

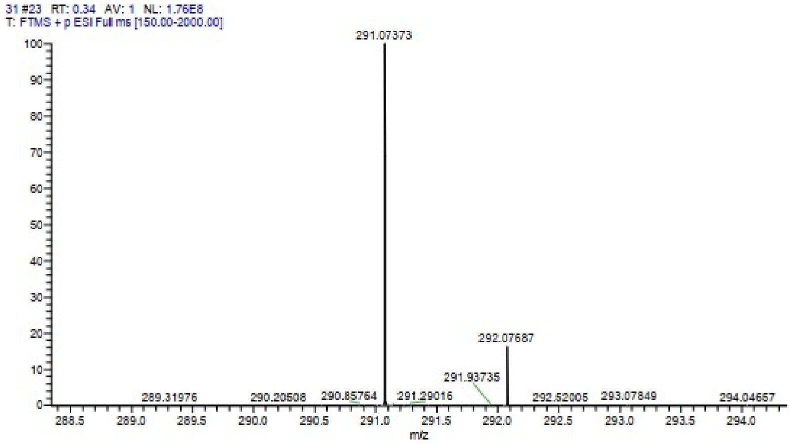

8-Methoxy-2-phenyl-4H-pyrido[1,2-a]pyrimidin-4-one(**3fa**)

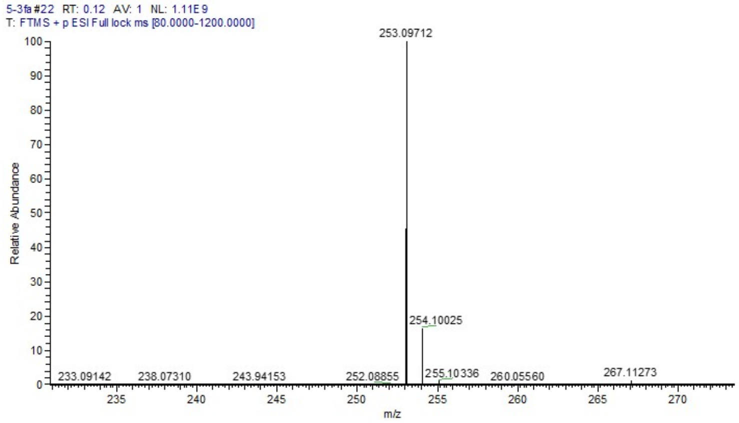

9-Methoxy-2-phenyl-4H-pyrido[1,2-a]pyrimidin-4-one(**3ga**)

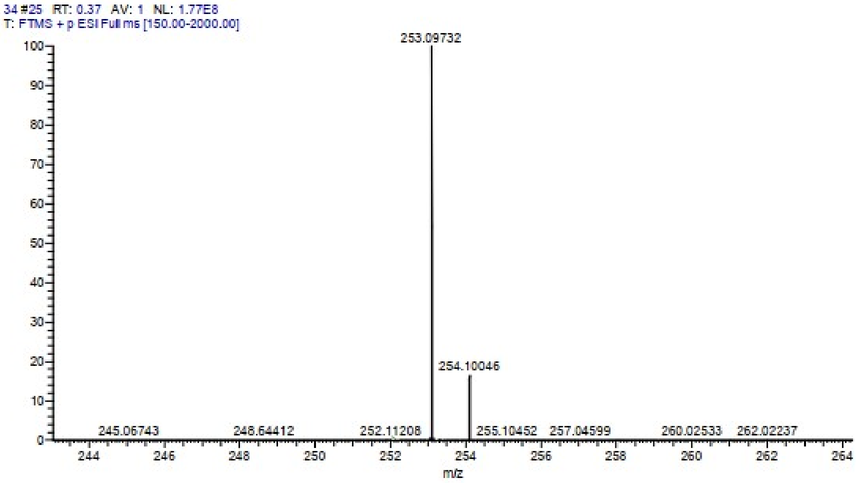

7-Methoxy-2-phenyl-4H-pyrido[1,2-a]pyrimidin-4-one(**3ha**)

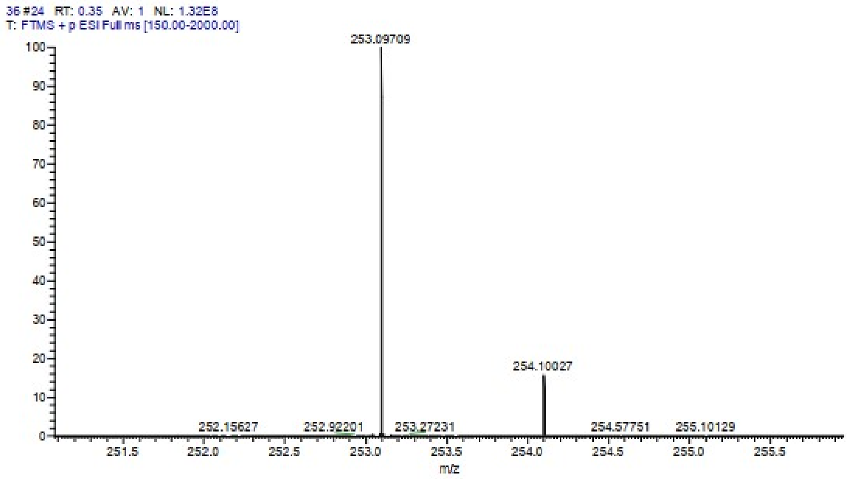

3. IR spectra of new compounds

2-(3-Fluorophenyl)-4H-pyrido[1,2-a]pyrimidin-4-one(**3ak**)

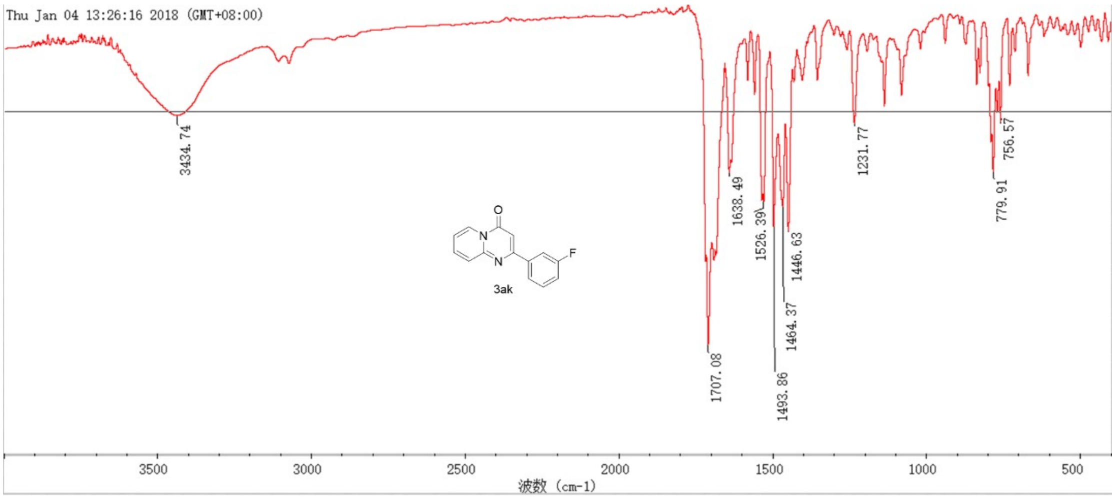

2-(2-Fluorophenyl)-4H-pyrido[1,2-a]pyrimidin-4-one(**3al**)

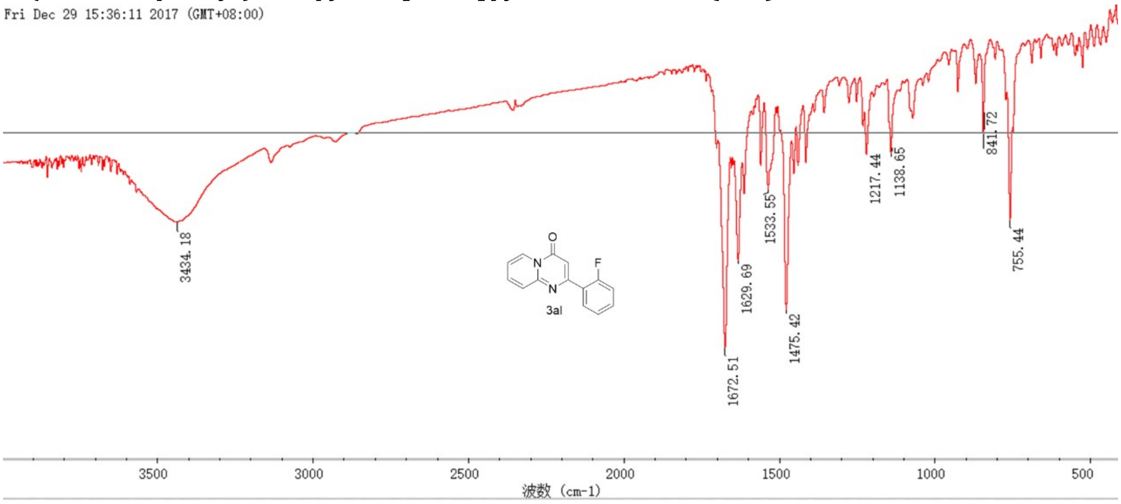

2-(3-(Trifluoromethyl)phenyl)-4H-pyrido[1,2-a]pyrimidin-4-one(**3ao**)

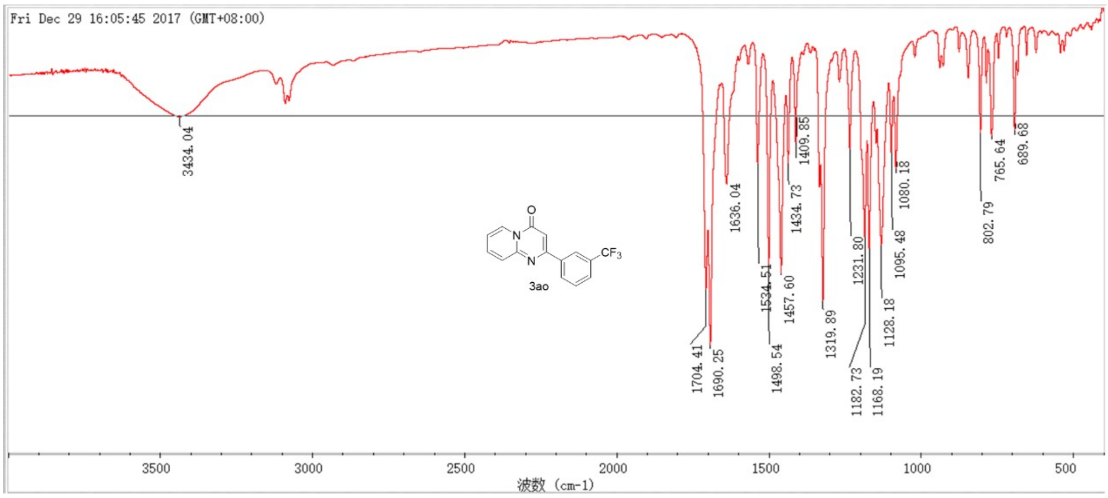

*8-Methoxy-2-phenyl-4H-pyrido[1,2-a]pyrimidin-4-one***(3fa)**

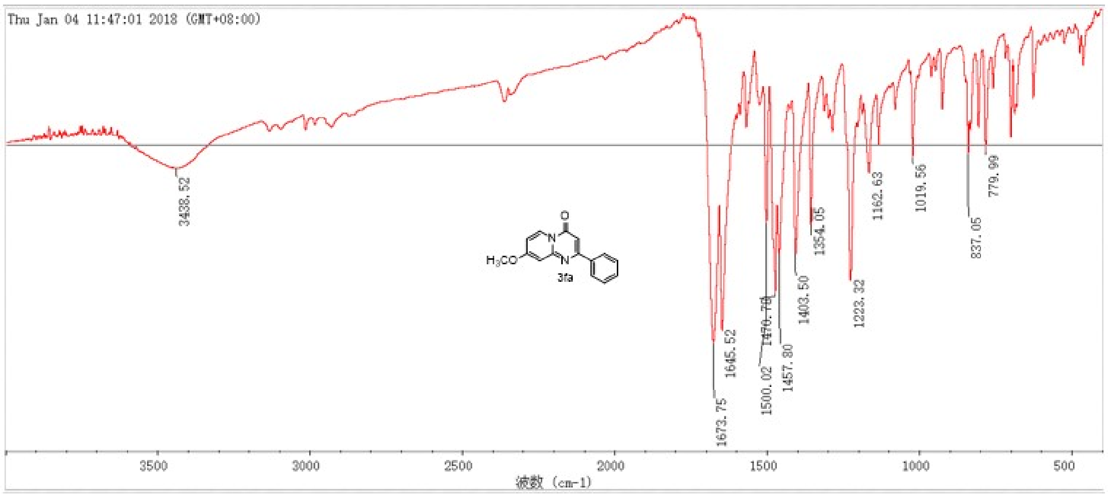

*9-Methoxy-2-phenyl-4H-pyrido[1,2-a]pyrimidin-4-one***(3ga)**

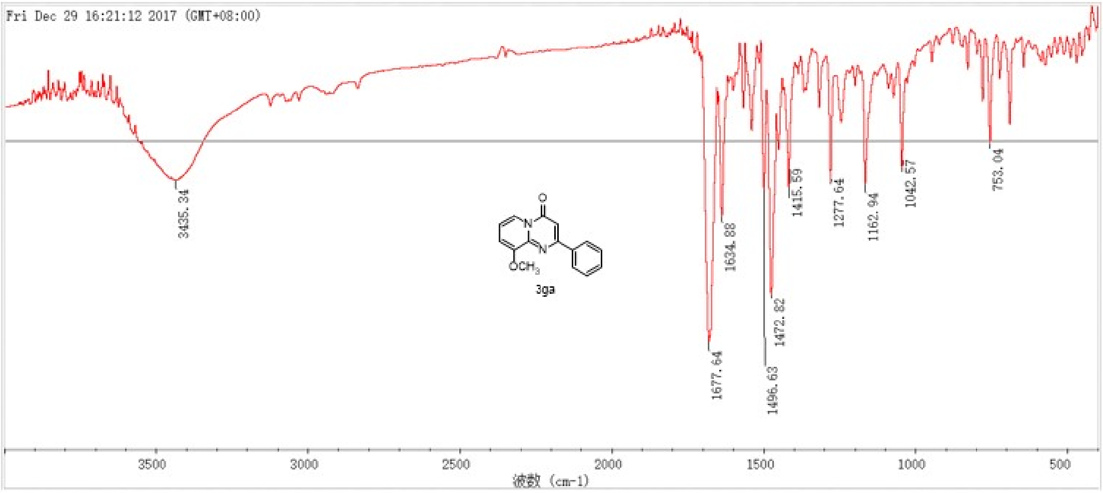

*7-Methoxy-2-phenyl-4H-pyrido[1,2-a]pyrimidin-4-one***(3ha)**

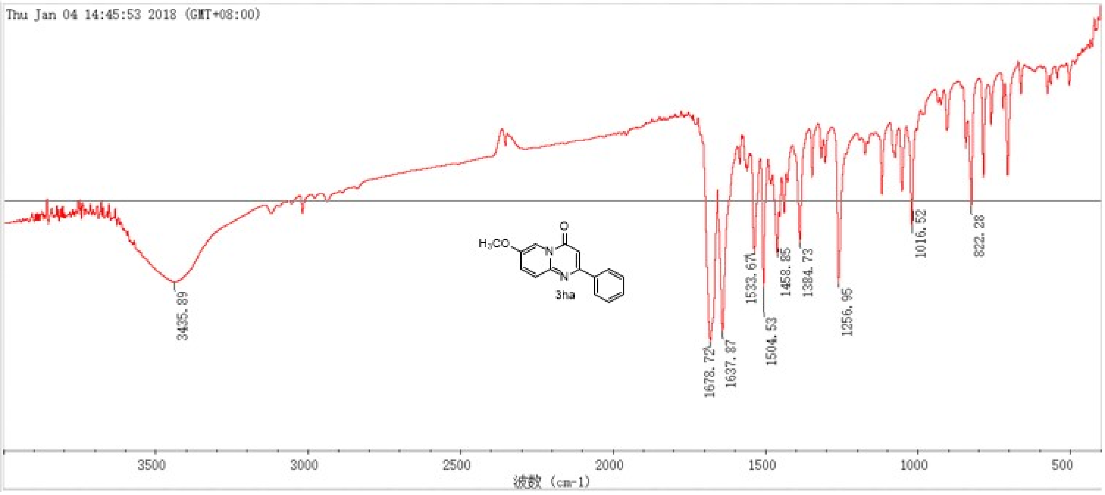

Supplement: RA-013-D3RA04454H-s001 [file RA-013-D3RA04454H-s001.pdf]
